# A thioether-directed palladium-cleavable linker for targeted bioorthogonal drug decaging†
†Electronic supplementary information (ESI) available: Detailed methods and additional characterisation. See DOI: 10.1039/c8sc00256h


**DOI:** 10.1039/c8sc00256h

**Published:** 2018-04-06

**Authors:** Benjamin J. Stenton, Bruno L. Oliveira, Maria J. Matos, Laura Sinatra, Gonçalo J. L. Bernardes

**Affiliations:** a Department of Chemistry , University of Cambridge , Lensfield Road , CB2 1EW Cambridge , UK . Email: gb453@cam.ac.uk; b Instituto de Medicina Molecular , Faculdade de Medicina da Universidade de Lisboa , Av. Prof. Egas Moniz , 1649-028 Lisboa , Portugal . Email: gbernardes@medicina.ulisboa.pt

## Abstract

We describe the development of a bifunctional linker that simultaneously allows site-specific protein modification and palladium-mediated bioorthogonal decaging. This was enabled by a thioether binding motif in the propargyl carbamate linker and a readily available palladium complex. We demonstrate the efficiency of this reaction by controlled drug release from a PEGylated doxorubicin prodrug in cancer cells. The linker can be easily installed into cysteine bearing proteins which we demonstrated for the construction of an anti-HER2 nanobody–drug conjugate. Targeted delivery of the nanobody drug conjugate showed effective cell killing in HER2+ cells upon palladium-mediated decaging.

## Abstract 2

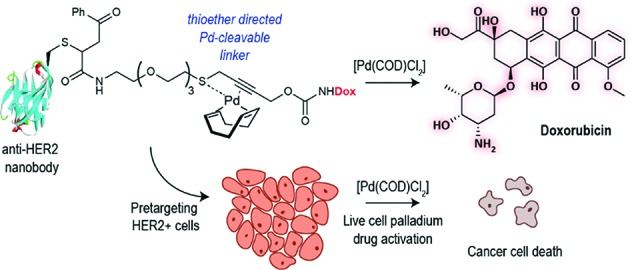
We describe the development of a bifunctional linker that simultaneously allows site-specific protein modification and palladium-mediated bioorthogonal decaging.

## Introduction

**Scheme 1:**
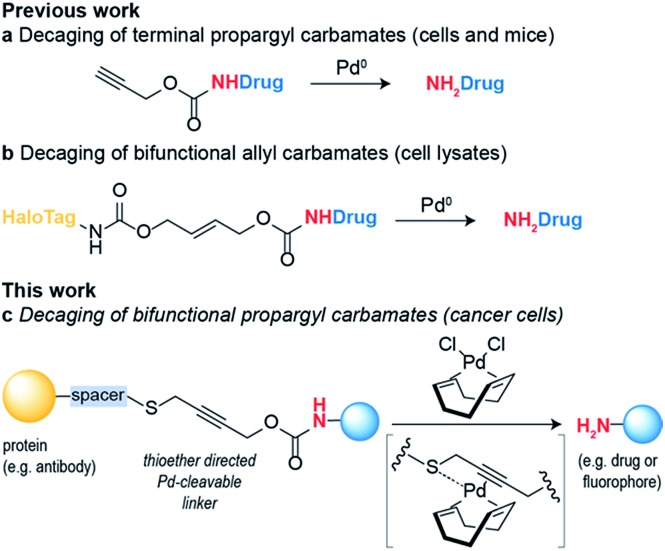
Palladium decaging for chemical biology.

Bioorthogonal reaction development has mainly focused on ligation reactions, but recently there have been many developments in bioorthogonal cleavage or “decaging” reactions.^1^ This has mainly focused on the removal of small caging groups from prodrugs and proteins using light,^2^,^3^ metal^4^,^5^ or chemical^6^,^7^ triggers. Notable among these is the use of propargyl carbamates as protecting groups for palladium-assisted drug release^8^,^9^ and protein activation within living cells^10^,^11^ (Scheme 1a). In these examples, synthetic caged anticancer drugs or genetically encoded lysine analogues are used. Strategies based on bioorthogonal palladium decaging have several advantages including fast reaction kinetics and enhanced biocompatibility of palladium catalysts. Just recently, a nanoencapsulated formulation of palladium complexes were shown to be active catalysts *in vivo* and could effectively treat tumors in mouse models.^12^ This approach has been mostly limited to the removal of monofunctional protecting groups from anticancer prodrugs or genetically encoded amino acid residues. In a single example, a bifunctional cleavable linker consisting of a small-molecule ligand and a reactive capture tag connected *via* a palladium cleavable linkage has been reported.^13^ This bifunctional linker was used in target pull-down assays, where a drug was immobilized on a “HaloTag solid-support” and later cleaved when the drug was bound to its target (Scheme 1b). The apparent versatility of these reactions and their potential for biological applications led us to focus on the development of a bifunctional propargyl carbamate linker that would simultaneously allow site-specific protein modification and palladium triggered decaging. The utility of this approach was demonstrated by building an antibody–drug conjugate (ADC) bearing a palladium-cleavable linker for controlled targeted drug-delivery (Scheme 1c).

## Results and discussion

**Fig. 1:**
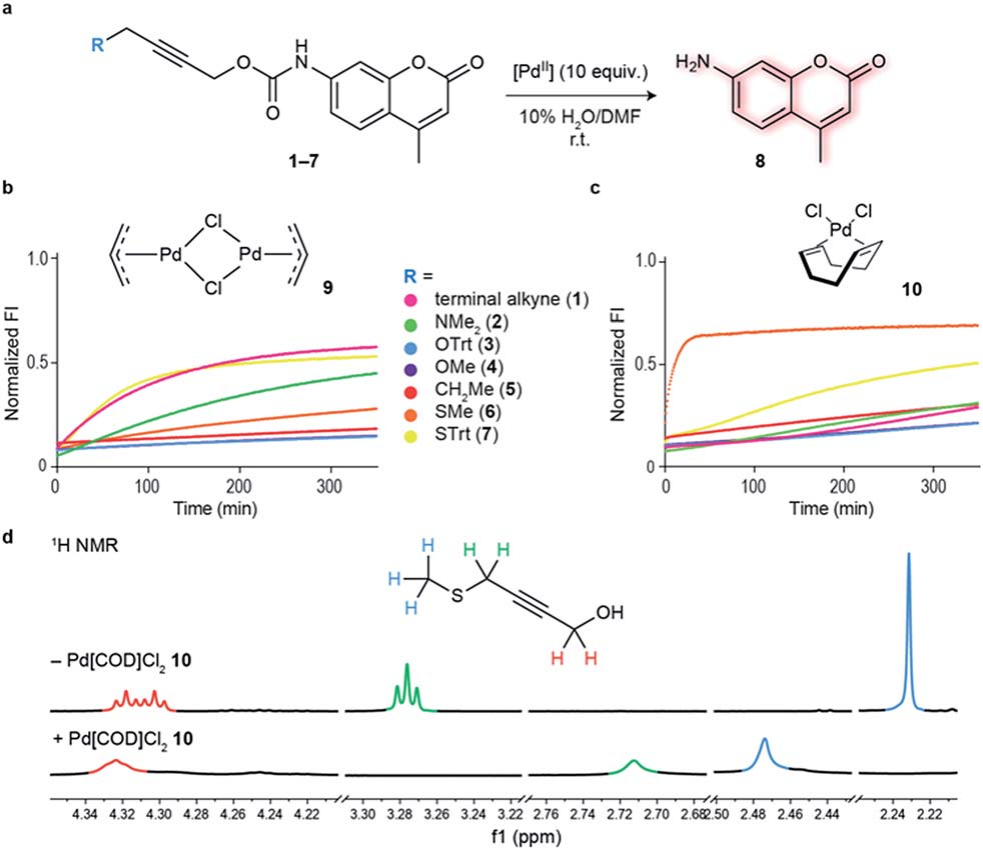
Functional group screening to determine strategy used to bifunctionalize propargyl carbamates. (a) Decaging reaction of substituted propargyl carbamate protected 7-amino-4-methylcoumarin **1–7** through reaction with palladium complexes. (b) Increase in fluorescence of decaging reactions over time. Propargyl carbamate protected fluorophore has quenched fluorescence which is restored upon decaging. The reactions were performed at 100 μM final concentration of the fluorophores, with 5 equiv. of allylpalladium(ii) chloride dimer **9**. (c) Fluorescence screening performed with 10 equiv. Pd(COD)Cl_2_**10**. The data were normalized with respect to 100 μM of free fluorophore (7-amino-4-methyl coumarin **8**) plus the final concentration of the palladium complex (0.5 mM for **9** and 1 mM for **10**). (d) ^1^H NMR data supporting hypothesis of binding of Pd(COD)Cl_2_ with methyl propargyl thioether motif.

Initial studies focused on exploring which functional groups were tolerated when extending the terminal propargyl carbamates to allow the synthesis of a bifunctional linker. We started by synthesizing caged coumarin derivatives **1–7** with different pendant S, N, O and C propargyl groups as a means to assess the efficiency of the palladium-mediated depropargylation reaction. The caged coumarin derivatives **1–7** have a quenched fluorescence which results in the formation of 7-amino-4-methyl coumarin **8** and a turn-on of fluorescence upon reaction with palladium complexes (Fig. 1a). Using allyl palladium chloride complex **9**,^11^ we found that amine **2**, ethers **3–4** and methylene **5** were all disfavored in this position (Fig. 1b). In contrast, thioethers **6–7** seemed favorable when used in conjunction with large appended groups such as trityl **7** (Fig. 1b). However the necessity of such large, lipophilic groups for efficient palladium decaging was considered a limitation for potential applications in chemical biology due to low aqueous solubility of such derivatives. We hypothesized that this issue could potentially be solved by using different palladium complexes bearing bulkier ligands as compared to the initially used allylpalladium complex **9** and allow decaging of thioether propargyl linkers bearing smaller, biocompatible pendant groups.

Allylpalladium(ii) chloride **9** is known to be reduced to palladium(0) in the presence of nucleophiles.^14^ Therefore, a nucleophilically activated palladium complex – Pd(COD)Cl_2_**10** (COD = 1,5-cyclooctadiene)^15^ was trialed in air and found to be most reactive with thioethers with small substituents appended to the propargyl carbamate (Fig. 1c). We were pleased to observe that the reaction of Pd(COD)Cl_2_**10** with methyl thioether derivative **6** proceeded faster and gave higher conversion when compared with the previously reported of a terminal propargyl carbamate **1** with allylpalladium(ii) chloride **9** under identical conditions.^11^ As palladium is thiophilic, it was thought that this may be due to a thioether–palladium–propargyl binding interaction (Scheme 1c). Directing groups have seen much use in transition metal mediated catalysis,^16^ and thioethers are often employed to guide palladium catalysts.^17^,^18^ In another example, thioethers were employed in bioconjugation reactions to direct a ruthenium cross-metathesis catalyst.^19^ This hypothesis was verified using ^1^H NMR studies with a truncated derivative of the propargyl methyl thioether derivative (Fig. 1d) which confirmed binding by complete shift of the thioether protons. As expected, the reactivity is diminished by replacement of a chloride with a methyl ligand on the palladium complex, Pd(COD)MeCl, as methyl would be a much less labile ligand than chloride (Fig. S2†). In addition, a palladium complex bearing a strained ligand – Pd(NBD)Cl_2_ (NBD = norbornadiene) – also led to lower decaging efficiency likely due to the slower reduction step (Fig. S2†). Other palladium precursors such as Pd(OAc)_2_ and Pd (MeCN)_2_Cl_2_ showed no reactivity under similar conditions when reacted with **6** (Fig. S3†).

**Fig. 2:**
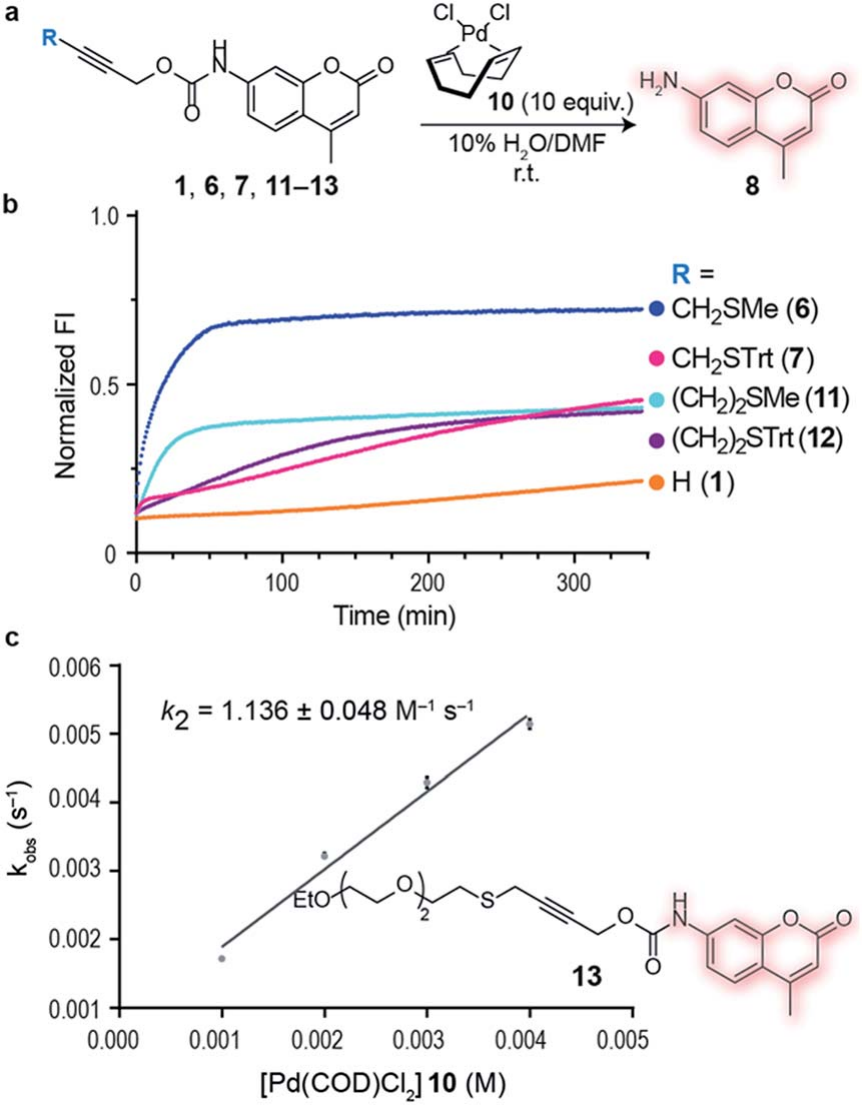
Alkyne–sulfur distance screening and kinetics of palladium-mediated decaging. (a) Decaging reaction of substituted thioether propargyl carbamate protected 7-amino-4-methyl coumarin **1**, **6**, **7**, **11–13** using Pd(COD)Cl_2_**10**. (b) Increase in fluorescence of decaging reactions over time. Propargyl carbamate protected fluorophore has quenched fluorescence which is restored upon decaging. The reactions were performed at 100 μM final concentration of the fluorophores, with 10 equiv. of Pd(COD)Cl_2_**10**. Data was normalized with respect to 100 μM of free fluorophore plus the final concentration of the palladium complex **10** (1 mM). (c) Kinetics of decaging of a PEGylated fluorophore. This was determined under pseudo-first order conditions using a 100 μM final concentration of the fluorophore and 10–40 equiv. of Pd(COD)Cl_2_**10**.

Having an efficient palladium complex in hand that allows fast decaging of substituted thioether propargyl carbarmates, we turned our attention to verify whether the distance between the alkyne and the sulfur atom could affect the transformation. For this, we synthesized a number of propargyl carbamate derivatives **11–13** bearing an extra methylene inserted between the alkyne and the thioether (see ESI† for synthetic details). We found that a single methylene **6–7** between the alkyne and sulfur gave the best conversion to the corresponding decaged product. It should be noted that the reaction still proceeds when two methylene substituents **11–12** separate the alkyne and sulfur atom providing further support of the proposed thioether–palladium–alkyne binding motif (Fig. 2a and b). Because smaller substituents about the alkyne seemed to promote a more efficient reaction with Pd(COD)Cl_2_, we designed and synthesized a polyethylene glycol (PEG) derivative **13** with increased hydrosolubility for kinetic studies. Under pseudo-first order conditions, the reaction was found to have a second order rate constant of *k*_2_ = 1.136 ± 0.048 M^–1^ s^–1^. This reaction rate is similar to those reported for the tetrazine:*trans*-cyclooctene (TCO) decaging reaction which varies between 0.54 ± 0.06 and 57.7 ± 5.0 M^–1^ s^–1^ depending on the tetrazine and solvent system used.^20^ One advantage of our system is the relative ease of synthesis of our bifunctional linker and availability of palladium complexes when compared to TCO and tetrazine derivatives. In addition, the use of PEG should form straight, soluble chains in water which would later allow synthesis of hydrosoluble prodrugs as well as extension of the palladium binding site from the surface of the protein of interest. It should be noted that the rate of our reaction while perhaps not fast enough for imaging biological processes, it is suitable for drug activation.

This newly developed bifunctional linker bearing a propargyl carbamate and a substituted thioether for appendage of higher order functionality was then applied to the decaging of a prodrug in cell culture. Doxorubicin (Dox) **14** was selected as a widely available chemotherapeutic agent with the necessary reactive amine, and the corresponding PEGylated prodrug (cDox) **15** was synthesized (see ESI† for synthetic details). Carbamates of Dox are known to be less toxic than the drug itself,^12^,^20^ possibly due to poorer cell membrane penetrating properties and also the charged ammonium in the free drug is responsible for forming strong ionic bond with phosphate in the DNA backbone which is no longer possible in the prodrug.^21^ We started by demonstrating the stability of cDox **15** in cellular media at 37 °C for 24 h using HPLC (Fig. S12†). When Pd(COD)Cl_2_**10** was added to cDox **15**, successful decaging and formation of Dox **14** was achieved after 24 h (Fig. S12†). Having shown stability of the prodrug **15** and subsequent palladium-mediated decaging in cellular media, we then turned our attention to cellular studies.

**Fig. 3:**
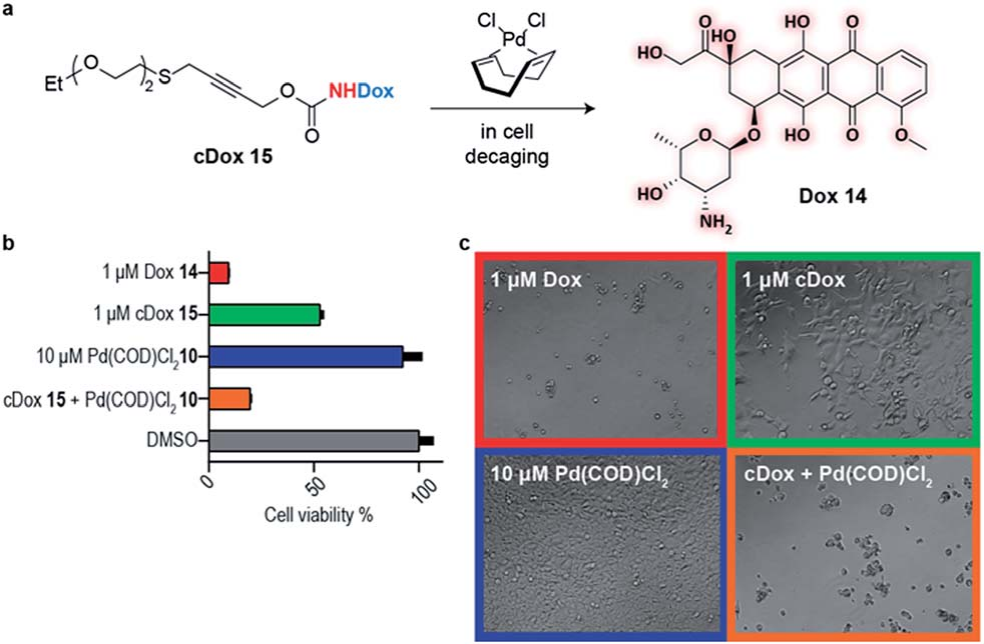
Live cell decaging of a palladium cleavable PEGylated doxorubicin prodrug (cDox) **15**. (a) General scheme for palladium directed decaging of cDox **15**. (b) Determination of cell viability of HEK 293 cells using the resazurin reduction assay (*i.e.* alamarBlue® cell viability assay protocol). Cell viability is determined based on cells' ability to reduce a bioprobe resazurin relative to the control with 1% DMSO. (c) Bright field microscopy showing cells after 96 h in various states of viability.

At the optimal concentration (1 μM) found during a toxicity screen for human embryonic kidney (HEK) 293 cells (Fig. S10†), the prodrug cDox **15** was approximately 10 times less toxic than the parent drug. Similarly, we screened Pd(COD)Cl_2_**10** toxicity and found that concentrations up to 10 μM added each 24 h did not influence cell viability up to 96 h in total as assessed using alamarBlue® cell viability assay protocol (Fig. S10†). We found both cDox cell viability window and Pd(COD)Cl_2_**10** biocompatibility to be sufficient to demonstrate the feasibility of our bioorthogonal drug-delivery approach. Notably, when the prodrug cDox **15** (1 μM) was treated with a non-toxic concentration of Pd(COD)Cl_2_**10** (10 μM each 24 h), a significant increase in toxicity was observed reaching similar cell killing efficiency as Dox **14** (Fig. 3a and b). These data were qualitatively corroborated by microscopy (Fig. 3c). This successful decaging demonstrates the applicability of the bifunctional thioether propargyl carbamate linker for palladium-directed activation of prodrugs in cell culture.

**Fig. 4:**
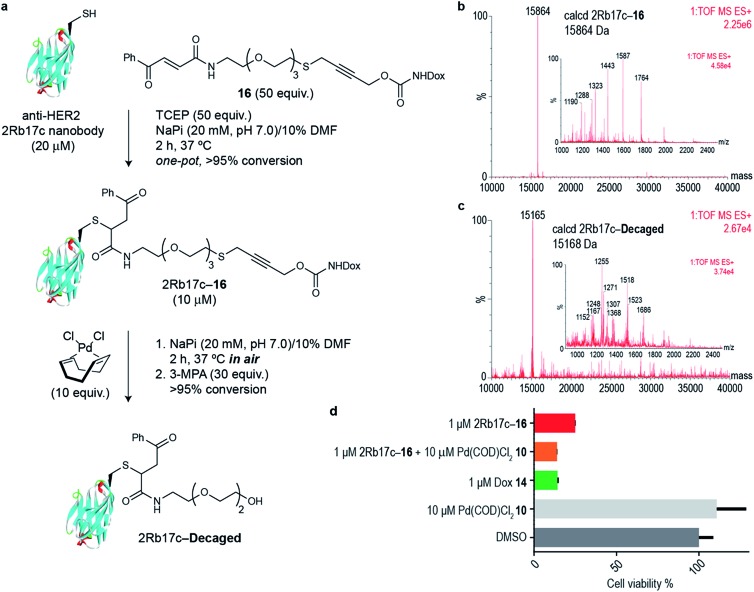
(a) *In vitro* decaging of the ADC characterized by LC-MS. (b) LC-MS of nanobody–drug conjugate 2Rb17c–**16**. (c) LC-MS of 2Rb17c–**16** after reaction with Pd(COD)Cl_2_**10**. (d) Cell viability of MCF-7 cells (HER2+) when treated with 2Rb17c–**16** and Pd(COD)Cl_2_**10**.

Next we decided to use the bifunctional linker we developed towards the design of palladium cleavable ADC. As an antibody, we chose a smaller antibody fragment that targets the HER2 antigen. The HER2 antigen has been validated in the clinic and is the target of the marketed ADC trastuzumab emtansine.^22^ In addition, the use of smaller antibody fragments such as the nanobody used will facilitate future *in vivo* pretargeting applications of this metal decaging method since their smaller size allows for rapid tumor accumulation while also offering superior tissue penetration.^23^,^24^ The anti-HER2 nanobody 2Rb17c displays a reactive, engineered cysteine in a flexible chain at C-terminus that is ideal to achieve site-selective bioconjugation.^25^ For bioconjugation, we chose a method based on carbonylacrylic reagents developed by our group that enables efficient and irreversible cysteine modification.^26^ We started by introducing a PEG spacer in the linker to increase aqueous solubility and allow sufficient distance between the antibody surface and the cleavage site in order to avoid nonproductive chelation of the palladium catalyst by the cysteine adjacent amino acid side chains (Fig. 4a). The PEGylated thioether propargyl carbamate Dox derivative **16** equipped with a carbonylacrylic moiety for cysteine selective conjugation was prepared (see ESI† for synthetic details) and tested in bioconjugation reactions with the 2Rb17c nanobody. Optimal conditions were found to include a reducing agent, tris(2-carboxyethyl)phosphine (TCEP), in the presence of **16** to avoid disulfide formation. Complete conversion to a chemically-defined ADC 2Rb17c–**16** was achieved after 2 h at 37 °C in sodium phosphate buffer pH 7.0 as assessed by liquid chromatography-mass spectrometry (LC-MS) (Fig. 4a and b). Next we tested the decaging hypothesis of an ADC featuring a bifunctional thioether propargyl carbamate linker. Reactions were performed in sodium phosphate buffer pH 7.0 at 37 °C and the crude reaction analysed using LC-MS. Using only 10 equiv. of Pd(COD)Cl_2_**10** (similar amount of palladium needed for the decaging of terminal propargyl carbamates)^11^ in air and after 2 h, complete consumption of the 2Rb17c–**16** and formation of the decaged product was observed (Fig. 4a and c). The product features a short PEG chain that can be explained by reaction of a Pd(0) species with the propargyl carbamate to give a Pd(ii)–allene species. The Pd(ii)–allene species could then undergo an elimination to give a vinyl ether which subsequently decomposes or hydrolyzes (see Fig. S9† for proposed mechanism). In order to improve resolution of the LC-MS, a palladium scavenger, 3-MPA, was required,^27^,^28^ to which the ADC was verified as stable (Fig. S8†). This experiment also demonstrated the stability of the ADC in the presence of thiols, an important feature for ADC development. Finally, we assessed whether this transformation could be performed in cell culture to release a drug from an ADC. Using the HER2 positive cell line MCF-7, we found the ADC 2Rb17c–**16** to be less toxic to cells than the free drug Dox **14** (Fig. 4d). It should be noted that we are using an internalizing antibody that undergoes endosomal and lysosomal processing after internalization which can lead to the release of the drug or a toxic derivative of the drug. Remarkably, we found that in the presence of Pd(COD)Cl_2_**10**, the ADC 2Rb17c–**16** becomes as toxic as the free drug Dox **14** and twice as toxic as the ADC alone at the same concentration (1 μM). One advantage of using Pd(COD)Cl_2_ is its lipophilicity, which potentially increases its membrane permeability as suggested by the calculated log *P* (0.95 ± 0.05) for this precursor using a shake-flask method for determining the octanol/water partition coefficient (details in the ESI†).^29^ Additionally, the ability of other palladium precursors (*e.g.* allyl_2_Pd_2_Cl_2_) to cross membranes and accumulate inside cells has been reported previously.^11^ Our data shows that successful and efficient metal mediated decaging from an ADC bearing the thioether propargyl carbamate bifunctional linker we developed can be achieved with non-toxic concentrations of palladium in cellular settings.

## Conclusions

In summary, we present a method that enables the construction of bifunctional propargyl carbamate conjugates and efficient palladium decaging. This is enabled by a thioether-directed palladium mechanism and it was demonstrated by the construction of a stable ADC using site-selective cysteine bioconjugation of a thioether propargyl carbamate linker bearing the anti-cancer drug Dox **14** to a nanobody against the HER2 antigen. Drug release occurs using non-toxic amounts of palladium under mild conditions and using identical or fewer equivalents of palladium that is required for bioorthogonal decaging of propargyl carbamate protected lysine^11^ or tyrosine^10^ residues on proteins. The directness of the thioether propargyl motif for palladium binding, the non-toxicity of palladium catalysts and facile synthetic access to bifunctional conjugates makes this a useful strategy for controlled metal mediated small molecule activation. Although we have demonstrated that the reaction rate is suitable for drug activation on cells, for *in vivo* applications the development of new palladium compounds is still needed to circumvent the lack of cell selectivity of the catalyst and the toxicity of the metal when used in non-complexed^30^ or nanoparticle-functionalised form.^31^ We are also exploring extensions of this strategy by using non-internalizing antibodies to allow exclusive extracellular decaging within the tumor microenvironment.

## Conflicts of interest

There are no conflicts to declare.

## Supplementary Material

Supplementary information
