# Supplementary material for: A thioether-directed palladium-cleavable linker for targeted bioorthogonal drug decaging
Source: Chem Sci. 2018 Apr 6;9(17):4185–9. doi: 10.1039/c8sc00256h (PMC5941270; doi:10.1039/c8sc00256h)
Supplement: Supplementary file 1 [file SC-009-C8SC00256H-s001.zip › SI_revised_to publish.pdf]

Table of Contents

1 General Information 2

2 Synthesis 3

3 Fluorescence Studies 30

4 Antibody Modification 33

4.1 Proposed Mechanism of Antibody Decaging 36

5 Cell Studies 37

6 NMR Data 40

7 References 99

1. General Information

All reactions were carried out under a positive pressure of argon dried over $CaCl_2$ unless otherwise stated. All reagents were purchased from commercial sources and used as received. Distilled ACN, DCM, MeOH, Et_2O and toluene were freshly distilled over CaH_2 under an atmosphere of argon. Distilled THF was pre-dried over CaH_2 and then distilled over $LiAlH_4/Ph_3CH$. Flash column chromatography was performed on Material Harvest 60 Å silica and preparative TLC was performed on Merck PLC 60 F_{254} plates. Analytical TLC was visualized by UV at 254 nm, ninhydrin or $KMnO_4$ stain as appropriate.

Characterisation

IR spectrometry was carried out on a Perkin Elmer Spectrum One ATR FT-IR, melting points on a Stanford Research Systems OptiMelt. NMR spectra were recorded on Bruker 400-AVIII, DPX-400 or 500-AVIII HD Smart Probe as appropriate. The residual solvent peaks were used as an internal reference for chemical shift (^{1}H NMR $CHCl_3$ δ 7.27 ppm, DMSO δ 2.50 ppm; ^{13}C NMR $CDCl_3$ δ 77.0 ppm DMSO δ 39.5 ppm). Data are presented as follows: chemical shift (ppm), multiplicity (s = singlet, d = doublet, t = triplet, q = quartet, m = multiplet, br = broad), coupling constant *J*, and integration. High resolution mass spectra were obtained with a Thermo Fischer Orbitrap or Waters Xevo LC-MS and ionized by electrospray (ESI). Absorbance and fluorescence spectra or endpoints were measured in a BMG Clariostar in Greiner 300 µL 96-well, U-bottom, clear polypropylene plates.

General method of fluorescence assay for decaging optimization

200 µM solutions of 7-amino-4-methyl coumarin (fluorophore) and caged-fluorophore derivatives were prepared in 10% deionized H_2O/DMF (v/v). Solutions of palladium catalysts were prepared to give twice their desired final concentration [Pd] in 10% deionized H_2O/DMF (v/v). 50 µL aliquots of caged fluorophore solutions were pipetted into a 96-well plate, and diluted with either 50 µL solvent or 50 µL palladium catalyst solution. Reaction progress was monitored at 23 °C by the fluorescence of 7-amino-4-methyl coumarin: λ_{abs} (max) 352 nm, λ_{em} (max) 415 nm, bandwidth 8 nm. The gain and focus of the plate reader were automatically adjusted before each assay based on 100 µM 7-amino-4-methyl coumarin. All reactions were performed in triplicate.

Partition coefficient (log P)

Octanol–saturated water and water–saturated octanol were prepared by stirring the mixture for 24 h. Aliquots of stock solutions of the Pd precursor in octanol–saturated water were added to the same volumes of water–saturated octanol. The samples were vortexed for 5 min. After partition by centrifugation the aqueous and octanol layers were transferred into cuvettes for concentration analysis by UV spectroscopy. Log P values (0.95 ± 0.05, mean of three separate determinations, two repeats with similar results) of the $Pd(COD)Cl_2$ complex were calculated using the equation $\log P_{o/w} = \log([Pd(COD)Cl_2]_{oct}/[Pd(COD)Cl_2]_{aq})$.

Protein characterisation by LC-MS

Liquid chromatography-mass spectrometry (LC-MS) was performed on a Xevo G2-S TOF mass spectrometer coupled to an Acquity UPLC system using an Acquity UPLC BEH300 C4 column (1.7 µm, 2.1% 50 mm). Solvents A (water with 0.1% formic acid) and B (71% acetonitrile, 29% water, and 0.1%

formic acid) were used as the mobile phase at a flow rate of 0.2 mL·min^{-1}. The gradient was programmed as follows: 72% A to 100% B for 25 min; then 100% B for 2 min and after that 72% A for 18 min. The electrospray source was operated with a capillary voltage of 2.0 kV and a cone voltage of 40 V. Nitrogen was used as the desolvation gas at a total flow of 850 L·h^{-1}. Total mass spectra were reconstructed from the ion series using the MaxEnt algorithm preinstalled on MassLynx software (v. 4.1 from Waters) according to the manufacturer's instructions. To obtain the ion series described, the major peak(s) of the chromatogram were selected for integration and further analysis.

Cell Studies

HEK (ATCC® CRL-1573™) and MCF-7 (ATCC® HTB-22™) cells, kindly provided by the David Klenerman (Department of Chemistry, University of Cambridge, UK) and Maria Mota (Instituto de Medicina Molecular, João Lobo Antunes, Portugal) groups respectively, were grown in a humidified incubator at 37 °C under 5% CO_2 with 90% humidity, and split before reaching confluence using Trypsin-EDTA solution 0.25%. HEK cells were grown in DMEM supplemented with heat-inactivated FBS, 100 units/mL penicillin, 100 µg/mL streptomycin and 0.25 µg/mL amphotericin B. MCF-7 cells were grown in high glucose DMEM (+ pyruvate) supplemented with 10% heat-inactivated FBS, 100 units/mL penicillin and 100 µg/mL streptomycin, 1% non-essential amino acids, 2 mM GlutaMax™ and 10 mM HEPES. Cell viability experiments with MCF-7 cells were performed without the presence of HEPES. Both cell lines were grown to 80% confluency before seeding in 200 µL at 5000 (HEK) and 3000 (MCF-7) cells/well into a Corning Costar 96-well clear, flat bottom plate. AlamarBlue assays to determine toxicity were performed by adding 20 µL AlamarBlue solution to each well and incubating: 4 h for HEK cells, 6 h for MCF-7. Fluorescence was then measured at: λ_{ex} 530-560 nm, λ_{em} 590 nm. Cell viability was calculated as per manufacturer guidelines. All cell culture reagents were purchased from Gibco Life Technologies (USA), unless otherwise stated.

2. Synthesis

Propargyl-7-amino-4-methylcoumarincarbamate (1)

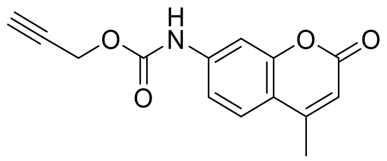

7-amino-4-methylcoumarin (50 mg, 1 eq., 0.285 mmol) was dissolved in dry DMF (1.5 mL) in an oven dried flask. K_2CO_3 (118 mg, 1.5 eq., 0.856 mmol) was added and the solution chilled to 0 ˚C. Propargyl chloroformate (0.04 mL, 1.5 eq., 0.856 mmol) was then added dropwise and the reaction stirred for 30 min before pouring over icewater (25 mL). The precipitate was filtered and washed with icewater (4 x 3 mL) then EtOH (3 x 3 mL) to give the desired product a yellow solid (22 mg, 0.0855 mmol, 30%). The product was used without further purification. The data were in accordance with literature.[1]

^{1}H NMR (400 MHz, DMSO-d$_6$) δ 10.36 (br. s, 1H; N*H*), 7.71 (d, J = 8.56 Hz, 1H; $H_{aromatic(5)}$), 7.49 - 7.57 (m, 1H; $H_{aromatic(8)}$), 7.41 (d, J = 8.56 Hz, 1H; $H_{aromatic(6)}$), 6.25 (s, 1H; $H_{aromatic(3)}$), 4.82 (s, 2H; OCH_2), 3.57 - 3.64 (m, 1H; C≡C*H*), 2.39 (s, 3H; CH_3)

4-(dimethylamino)but-2-yn-1-ol (2a)

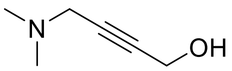

A solution of 2.5 M ^{n}BuLi in hexanes (1.17 mL, 1.2 eq., 2.92 mmol) in freshly distilled THF (12 mL) was chilled to -78 ˚C in an oven dried flask, and 3-dimethylamino-1-propyne (0.26 mL, 1 eq., 2.44 mmol) added dropwise. After 1 h, $(CH_2O)_n$ (146 mg, 2 eq., 4.88 mmol) was added in one portion and the reaction allowed to warm to room temperature. After 1 h, the reaction was quenched with brine (20 mL) and extracted with EtOAc (3 x 20 mL). The organics were dried over $MgSO_4$ and evaporated *in vacuo* to give the desired product as a yellow oil (219 mg, 1.93 mmol, 80%). The data were in accordance with the literature.[2]

^{1}H NMR (400 MHz, $CDCl_3$) δ 4.27 (t, *J* = 2.15 Hz, 2H; OCH_2), 3.25 (t, *J* = 2.15 Hz, 2H; NCH_2), 2.29 (s, 6H; CH_3)

4-(dimethylamino)but-2-ynl (7-amino-4-methylcoumarin)carbamate (2)

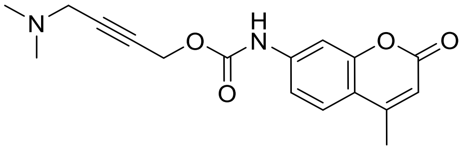

7-amino-4-methylcoumarin (60 mg, 1 eq., 0.339 mmol) and triphosgene (50 mg, 0.5 eq., 0.169 mmol) were dissolved in freshly distilled toluene (10 mL) and refluxed for 0.5 h in oven dried glassware before evaporating *in vacuo*. The crude isocyanate was then suspended in freshly distilled THF (5 mL) at room temperature and alcohol **2a** (57 mg, 1.5 eq., 0.508 mmol) added followed by dibutyltin dilaurate (0.01 mL, 0.06 eq., 20.4 μmol). After 1 h, the product was precipitated by dropwise addition of water (10 mL), chilling to 0 ˚C, and dropwise addition of Na_2CO_3 until pH 10. Futher water (5 mL) was added dropwise and the suspension stirred for 10 min. The precipitate was filtered and washed with MeOH (3 mL), Et_2O (2 x 3 mL) and petrol (3 mL), then dried *in vacuo* to give the desired product as an off-white powder (37 mg, 0.117 mmol, 35%).

Decomp. 178 ˚C

IR (ATR) / cm^{-1}: 3277, 2954, 2931, 2856, 1736,, 1687, 1624, 1591, 1530

^{1}H NMR (500 MHz, DMSO-d$_6$) δ 7.71 (d, *J* = 8.78 Hz, 1H; H_5), 7.54 (d, *J* = 1.91 Hz, 1H; H_8), 7.41 (dd, *J* = 1.91, 8.78 Hz, 1H; H_6), 6.25 (s, 1H; H_3), 4.85 - 4.88 (m, 2H; OCH_2), 3.25 - 3.30 (m, 2H; NCH_2), 2.39 (s, 3H; CH_3), 2.17 (s, 6H; N(CH_3)$_2$)

^{13}C NMR (126 MHz, DMSO-d$_6$) δ 160.5, 154.3, 153.6, 153.0, 143.0, 126.6, 115.0, 114.8, 112.5, 105.0, 82.9, 80.1, 53.2, 47.6, 44.1, 18.5

MS(ESI): calcd. for $C_{15}H_{20}N_2O_4Na$ $[M+Na]^+$ 315.1215, found 315.1322

Propargyl trityl ether (3a)

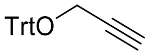

Propargyl alcohol (1.75 mL, 1 eq., 30.0 mmol) was dissolved in distilled DCM (15 mL) with 4 Å mol. sieves. DMAP (73 mg, 0.02 eq., 0.600 mmol) and pyridine (2.45 mL, 1.01 eq., 30.0 mmol) were added, followed by triphenylmethyl chloride (9.2 g, 1.1 eq., 33.0 mmol) in one portion and the reaction stirred at room temperature for 18 h. The reaction was then poured onto ice (200 mL) and then acidified to < pH 2 with 3 M HCl. The aqueous mixture was then extracted with EtOAc (3 x 150 mL). The organics were washed with icewater/brine (200 mL), dried over $MgSO_4$ and evaporated *in vacuo*. The crude material was dry-loaded onto silica (30 g) with EtOAc, and purified by flash chromatography with a gradient of 1-5% Et_2O/petrol, on silica (188 g) to give the product as a white powder (4.04 g, 13.5 mmol, 45%). The data were in accordance with literature.[3]

$\mathbf{R_f}$ (10% Et_2O/petrol) 0.40

^{1}H NMR (400 MHz, $CDCl_3$) δ 7.51 (d, *J* = 7.58 Hz, 6H; $H_{aromatic}$), 7.34 (t, *J* = 7.58 Hz, 6H; $H_{aromatic}$), 7.28 (t, *J* = 7.09 Hz, 3H; $H_{aromatic}$), 3.80 (d, *J* = 1.96 Hz, 2H; CH_2), 2.40 - 2.44 (m, 1H; C≡C*H*)

4-(trityloxy)but-2-yn-1-ol (3b)

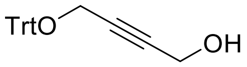

Propargyl trityl ether **3a** (2 g, 1 eq., 6.70 mmol) and $(CH_2O)_n$ (604 mg, 3 eq., 20.1 mmol) were dried separately under high vacuum overnight, before dissolving **3a** in distilled THF (40 mL) and chilling the solution to -78 ˚C in an oven dried flask. 2.5 M ^{n}BuLi in hexanes (2.95 mL, 1.1 eq., 7.37 mmol) was then added dropwise, and the reaction stirred at -78 ˚C for 10 mins. $(CH_2O)_n$ was then added in one portion, and the reaction stirred for 2 h before warming to room temperature and stirring for a further 1 h. The reaction was poured over ice (100 mL) and extracted with EtOAc (3 x 100 mL). The organics were washed with icewater/brine (50 mL), dried over $MgSO_4$ and evaporated *in vacuo*. The crude material was dry-loaded onto silica (10 g) and purified by flash chromatography with 30% EtOAc/petrol on silica (193 g) to give product as a white powder (1.71 g, 5.23 mmol, 78%). The data were in accordance with literature.[4]

^{1}H NMR (400 MHz, $CDCl_3$) δ 7.51 (d, *J* = 7.5 Hz, 6H; $H_{aromatic}$), 7.35 (t, *J* = 7.5 Hz, 6H; $H_{aromatic}$), 7.28 (t, *J* = 7.09 Hz, 3H; $H_{aromatic}$), 4.29 (s, 2H; CH_2), 3.87 (s, 2H; CH_2)

7-isocyanato-4-methylcoumarin (3c)

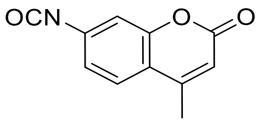

7-amino-4-methylcoumarin (100 mg, 1 eq., 0.570 mmol) and triphosgene (85 mg, 0.5 eq., 0.285 mmol) were dissolved in freshly distilled toluene (20 mL) and refluxed for 0.5 h in oven dried glassware. The

reaction mixture was then evaporated *in vacuo* to give the desired product which was used without further purification.

^{1}H NMR (400 MHz, DMSO-d_6) δ 7.41 (d, *J* = 8.56 Hz, 1H; H_5), 6.57 (dd, *J* = 1.47, 8.56 Hz, 1H; H_6), 6.41 (d, *J* = 1.47 Hz, 1H; H_8), 5.90 (s, 1H; H_3), 2.30 (s, 3H; CH_3)

MS(ESI): calcd. for $C_{11}H_8NO_3Na$ $[M+H]^+$ 202.0499, found 202.0492

4-(trityloxy)but-2-ynl (7-amino-4-methylcoumarin)carbamate (3)

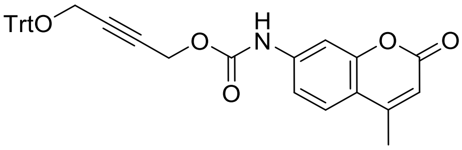

Isocyanate **3c** (40 mg, 1 eq., 0.199 mmol) and alcohol **3b** (72 mg, 1.1 eq., 0.219 mmol) were dissolved in freshly distilled THF (5 mL) at room temperature in an oven dried flask. Dibutyltin dilaurate (0.01 mL, 0.08 eq., 16.9 μmol) was added, and the reaction stirred for 1 h. The product was precipitated by dropwise addition of water (15 mL) and filtered. The precipitate was washed with MeOH (3 x 3 mL), Et_2O (3 x 3 mL) and petrol (3 x 3 mL), then dried *in vacuo* to give the desired product as a white powder (77 mg, 0.145 mmol, 73%).

m.p. 192.1-194.9 ˚C

IR (ATR) / cm^{-1}: 3281, 1734, 1697, 1619, 1582, 1534

^{1}H NMR (400 MHz, DMSO-d_6) δ 10.38 (s, 1H; N*H*), 7.71 (d, *J* = 8.86 Hz, 1H; H_5), 7.55 (d, *J* = 1.70 Hz, 1H; H_8), 7.42 (dd, *J* = 1.70, 8.86 Hz, 1H; H_6), 7.33 - 7.40 (m, 12H; $H_{aromatic}$), 7.24 - 7.32 (m, 3H; $H_{aromatic}$), 6.25 (s, 2H; H_3), 4.84 - 4.87 (m, 2H; OCH_2), 3.77 - 3.80 (m, 2H; OCH_2), 2.39 (s, 3H; CH_3)

^{13}C NMR (101 MHz, DMSO-d_6) δ 160.4, 154.3, 153.6, 152.9, 143.5, 142.9, 128.5, 127.7, 126.6, 115.0, 114.7, 112.5, 105.0, 87.4, 83.6, 80.8, 67.5, 53.0, 25.6, 18.5

MS(ESI): calcd. for $C_{34}H_{27}NO_5Na$ $[M+Na]^+$ 552.1781, found 552.1763

4-methoxybut-2-yn-1-ol (4a)

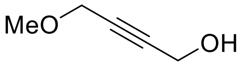

Methyl propargyl ether (0.85 mL, 1 eq., 10.0 mmol) was dissolved in freshly distilled THF (30 mL) in an oven dried flask. The solution was cooled to 0 ˚C, and 2.5 M ^{n}BuLi in hexanes (4.4 mL, 1.1 eq., 11.0 mmol) was added dropwise. The reaction was stirred for 30 min at 0 ˚C before $(CH_2O)_n$ (0.45 g, 1.5 eq., 15.0 mmol) was added in one portion. After 1 h at 0 ˚C, the reaction was diluted with sat. NH_4Cl solution (50 mL) and extracted with EtOAc (3 x 80 mL). The organics were washed with icewater/brine (50 mL), dried over $MgSO_4$ and evaporated *in vacuo* to yield the product as a light yellow liquid (738 mg, 7.37 mmol, 74%). The data were in accordance with the literature.[5]

^{1}H NMR (400 MHz, $CDCl_3$) δ 4.27 - 4.33 (m, 2H; OC*H*$_2$), 4.10 - 4.16 (m, 2H; OC*H*$_2$), 3.38 (s, 3H; OC*H*$_3$), 2.74 (br. s, 1H; O*H*)

4-methoxybut-2-ynl (7-amino-4-methylcoumarin)carbamate (4)

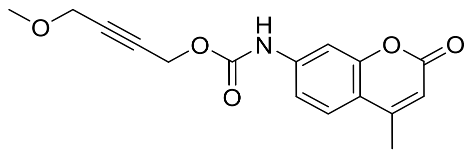

Isocyanate **3c** (40 mg, 1 eq., 0.199 mmol) and alcohol **4a** (25 mg, 1.26 eq., 0.249 mmol) were dissolved in freshly distilled THF (5 mL) at room temperature in an oven dried flask. Dibutyltin dilaurate (0.01 mL, 0.08 eq., 16.9 μmol) was added, and the reaction stirred for 1 h. The precipitate was filtered washed with water (4 x 3 mL), MeOH (4 x 3 mL), Et_2O (3 x 3 mL) and petrol (3 x 3 mL), then dried *in vacuo* to give the desired product as an off-white powder (27 mg, 0.0896 mmol, 45%).

Decomp. 186 ˚C

IR (ATR) / cm^{-1}: 2928, 2323, 1726, 1689, 1618

^{1}H NMR (500 MHz, DMSO-d$_6$) δ 10.37 (br. s, 1H; N*H*), 7.70 (d, *J* = 8.78 Hz, 1H; *H*$_5$), 7.53 (d, *J* = 1.91 Hz, 1H; *H*$_8$), 7.40 (dd, *J* = 1.91, 8.78 Hz, 1H; *H*$_6$), 6.24 (d, *J* = 0.92 Hz, 1H; *H*$_3$), 4.89 (br. t, *J* = 1.80 Hz, 1H; OC*H*$_2$), 4.16 (br. t, *J* = 1.80 Hz, 1H; OC*H*$_2$), 3.27 (s, 3H; OC*H*$_3$), 2.39 (d, *J* = 0.92 Hz, 3H; C*H*$_3$)

^{13}C NMR (126 MHz, DMSO-d$_6$) δ 160.5, 154.2, 153.6, 152.9, 142.8, 126.6, 115.0, 114.8, 112.5, 105.0, 83.6, 81.6, 59.4, 57.4, 53.1, 18.5

MS(ESI): calcd. for $C_{16}H_{15}NO_5Na$ $[M+Na]^+$ 324.0842, found 324.0827

Hex-2-yn-1-yl (7-amino-4-methylcoumarin)carbamate (5)

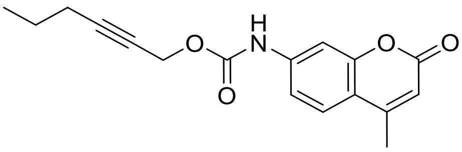

Triphosgene (85 mg, 1 eq., 0.285 mmol) was dissolved in DCM (10 mL) (dried over 4 Å mol. sieves) and then chilled to 0 ˚C. 2-hexyn-1-ol (0.09 mL, 3 eq., 0.856 mmol) was added dropwise followed by pyridine (0.07 mL, 3 eq., 0.856 mmol). The reaction was allowed to warm to room temperature and stirred for 3 h before evaporating *in vacuo*. The crude solids were triturated with freshly distilled Et_2O (3 x 4 mL) and the filtrate evaporated *in vacuo* to give the crude chloroformate. This was added dropwise to a suspension of 7-amino-4-methylcoumarin (50 mg, 1 eq., 0.285 mmol) and K_2CO_3 (59 mg, 1.5 eq., 0.428 mmol) in dry DMF (1 mL). To ensure complete addition, the chloroformate flask was rinsed with further dry DMF (0.5 mL). The reaction was stirred at 0 ˚C for 30 min then icewater (5 mL) was added slowly dropwise before pouring the mixture over icewater (20 mL). The precipitate was filtered and washed with icewater (4 x 3 mL) then EtOH (3 x 3 mL) to give the desired product as an off-white powder (27 mg, 0.0902 mmol, 32%).

Decomp. 208 ˚C

IR (ATR) / cm^{-1}: 3276, 2958, 1734, 1688, 1622, 1590, 1528

^{1}H NMR (400 MHz, DMSO-d_6) δ 10.32 (br. s, 1H; N*H*), 7.70 (d, J = 8.56 Hz, 1H; $H_{aromatic(5)}$), 7.53 (s, 1H; $H_{aromatic(8)}$), 7.41 (d, J = 8.56 Hz, 1H; $H_{aromatic(6)}$), 6.24 (s, 1H; $H_{aromatic(3)}$), 4.80 (s, 2H; OCH_2), 2.39 (s, 3H; CH_3), 2.23 (t, J = 6.9 Hz, 2H; CH_2CH_2), 1.48 (sxt, J = 6.9 Hz, 2H; CH_2CH_3), 0.94 (t, J = 6.9 Hz, 3H; CH_2CH_3)

^{13}C NMR (126 MHz, DMSO-d_6) δ 165.2, 159.0, 158.4, 157.8, 147.7, 131.2, 119.7, 119.5, 117.2, 109.7, 92.5, 80.2, 58.2, 26.6, 25.1, 23.2, 18.4

MS(ESI): calcd. for $C_{17}H_{17}O_4NNa$ $[M+Na]^+$ 322.1050, found 322.1043

4-(methylthio)but-2-yn-1-ol (6a)

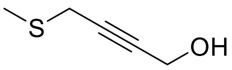

Sodium methanethiolate (500 mg, 1.5 eq., 7.13 mmol) was suspended in dry DMF (5 mL) in an oven dried flask, and chilled in a room temperature water bath. 80% propargyl bromide in toluene (0.53 mL, 1 eq., 4.76 mmol) was added dropwise, and the reaction stirred for 5 h. The reaction mixture was then poured over icewater basified to > pH 10 with sat. Na_2CO_3 solution (100 mL), and diluted with Et_2O (100 mL). The organics were washed with icewater basified to > pH 10 with sat. Na_2CO_3 solution (2 x 80 mL), then icewater/brine (50 mL), dried over $MgSO_4$ and then further dried over 4 Å mol. sieves. This solution was transferred *via* cannula to an oven dried flask, and then chilled to -78 ˚C. 2.5 M ^{n}BuLi in hexanes (2.85 mL, 1.5 eq., 7.13 mmol) was then added dropwise, and the reaction stirred for 10 min before $(CH_2O)_n$ (428 mg, 3.0 eq., 14.3 mmol) was added. The reaction was allowed to warm to room temperature and stirred for 1 h. The reaction mixture was washed with icewater (100 mL), and the aqueous layer was further extracted with Et_2O (100 mL). The organics were washed with brine (50 mL), dried over $MgSO_4$ and evaporated. The crude oil was purified by flash chromatography with 50% Et_2O/petrol on silica (49 g) to give the desired product as a malodorous, cloudy, viscous oil (146 mg, 1.25 mmol, 26%).

R_f (50% Et_2O/petrol) 0.26

^{1}H NMR (400 MHz, $CDCl_3$) δ 4.32 (s, 2H; OCH_2), 3.26 - 3.31 (m, 2H; SCH_2), 2.24 (s, 3H; CH_3)

^{13}C NMR (126 MHz, $CDCl_3$) δ 81.7, 81.0, 51.1, 21.7, 15.3

MS(ESI): calcd. for $C_5H_8O^{32}SNa$ $[M+Na]^+$ 139.0188, found 139.0188

4-(methylthio)but-2-ynl (7-amino-4-methylcoumarin)carbamate (6)

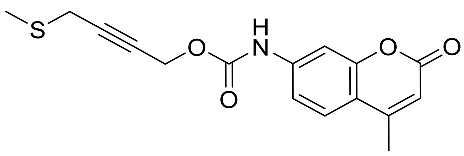

Isocyanate **3c** (40 mg, 1 eq., 0.199 mmol) and alcohol **6a** (29 mg, 1.26 eq., 0.251 mmol) were dissolved in freshly distilled THF (5 mL) at room temperature. Dibutyltin dilaurate (0.01 mL, 0.08 eq., 17.4 μmol) was added, and the reaction stirred for 1 h. The reaction mixture was evaporated to ¾ its volume, and the product precipitated by dropwise addition of water (4 mL). The precipitate was washed with water (4 x 3 mL), MeOH (3 x 3 mL), Et_2O (3 x 3 mL) and petrol (3 x 3 mL), then dried *in vacuo* to give the desired product as a white powder (38 mg, 0.121 mmol, 61%).

Decomp. 208 ˚C

IR (ATR) / cm^{-1}: 3276, 3172, 3100, 1738, 1687, 1627, 1593, 1534

^{1}H NMR (400 MHz, DMSO-d_6) δ 10.37 (br. s, 1H; N*H*), 7.71 (d, *J* = 8.51 Hz, 1H; H_5), 7.53 (d, *J* = 1.36 Hz, 1H; H_8), 7.41 (dd, *J* = 1.36, 8.51 Hz, 1H; H_6), 6.25 (s, 1H; H_3), 4.85 - 4.88 (m, 2H; OCH_2), 3.41 - 3.44 (m, 2H; SCH_2), 2.39 (s, 3H; CH_3), 2.16 (s, 3H; SCH_3)

^{13}C NMR (101 MHz, DMSO-d_6) δ 160.5, 154.3, 153.6, 152.9, 142.8, 126.5, 115.0, 114.7, 112.5, 105.0, 83.8, 77.6, 53.3, 20.9, 18.5, 15.0

MS(ESI): calcd. for $C_{16}H_{15}NO_4{}^{32}SNa$ $[M+Na]^+$ 340.0614, found 340.0598

Propargyl trityl thioether (7a)

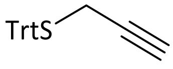

K_2CO_3 (750 mg, 3 eq., 5.43 mmol) was dried under high vacuum for 2 h before adding dry DMF (5 mL), followed by 80% propargyl bromide in toluene (0.6 mL, 3 eq., 5.43 mmol). Triphenylmethane thiol (500 mg, 1 eq., 1.81 mmol) was then added in one portion at room temperature and the reaction stirred at room temperature for 6 h. The reaction was then diluted in Et_2O (80 mL) and washed with icewater (2 x 30 mL), then icewater/brine (30 mL). The organics were dried over $MgSO_4$, and evaporated *in vacuo* to give the desired product as a light yellow solid (457 mg, 1.45 mmol, 80%).

R_f (petrol) 0.19

m.p. 127.5-130.5 ˚C

IR (ATR) / cm^{-1}: 3299, 3065, 1593, 1485, 1441

^{1}H NMR (400 MHz, $CDCl_3$) δ 7.44 (d, *J* = 7.58 Hz, 6H; $H_{aromatic}$), 7.30 - 7.35 (m, 6H; $H_{aromatic}$), 7.23 - 7.29 (m, 3H; $H_{aromatic}$), 2.83 (d, *J* = 2.5 Hz, 2H; CH_2), 2.19 (t, *J* = 2.5 Hz, 1H; C≡C*H*)

^{13}C NMR (101 MHz, $CDCl_3$) δ 144.0, 129.5, 128.1, 126.8, 79.1, 71.8, 67.2, 20.2

MS(ESI): calcd. for $C_{22}H_{18}SNa$ $[M+Na]^+$ 337.1021, found 337.1033

4-(tritylthio)but-2-yn-1-ol (7b)

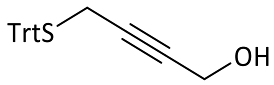

Propargyl trityl thioether **7a** (100 mg, 1 eq., 0.318 mmol) was added to an oven dried flask and dissolved in freshly distilled THF (5 mL). The solution was cooled to -78 ˚C before adding 2.5 M ^{n}BuLi in hexanes (0.14 mL, 1.1 eq., 0.350 mmol) dropwise. After 20 min stirring at -78 ˚C, $(CH_2O)_n$ (30 mg, 3 eq., 0.715 mmol) that had been previously dried under high vacuum overnight was added in one portion. After a further 20 min stirring, the reaction was warmed to room temperature and stirred for 4 h. The reaction mixture was poured over ice (30 mL) and extracted with EtOAc (2 x 50 mL). The organics were washed with icewater/brine (30 mL), dried over $MgSO_4$, filtered and evaporated. The crude material was purified by prep. TLC (2 mm, 25% EtOAc/petrol) to give the desired product as a yellow waxy solid (88 mg, 0.255 mmol, 80%).

R_f (25% EtOAc/petrol) 0.23

IR (ATR) / cm^{-1}: 3377, 3056, 2924, 1594, 1486, 1444

^{1}H NMR (400 MHz, $CDCl_3$) δ 7.46 (d, *J* = 7.7 Hz, 6H; $H_{aromatic}$), 7.34 (t, *J* = 7.7 Hz, 6H; $H_{aromatic}$), 7.23 - 7.29 (m, 3H; $H_{aromatic}$), 4.18 - 4.22 (m, 2H; SCH_2), 2.88 - 2.92 (m, 2H; CH_2O), 1.59 (br. s., 1H; OH)

^{13}C NMR (101 MHz, $CDCl_3$) δ 144.1, 129.6, 128.0, 126.9, 81.9, 81.2, 67.2, 51.2, 20.5

MS(ESI): calcd. for $C_{23}H_{20}O^{32}SNa$ $[M+Na]^+$ 367.1127, found 367.1129

4-(tritylthio)but-2-ynl (7-amino-4-methylcoumarin)carbamate (7)

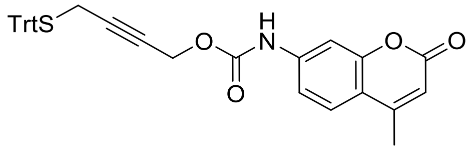

Isocyanate **3c** (57 mg, 1 eq., 0.283 mmol) and alcohol **7b** (98 mg, 1 eq., 0.283 mmol) were dissolved in freshly distilled THF (5 mL) at room temperature in an oven dried flask. Dibutyltin dilaurate (0.01 mL, 0.06 eq., 16.9 µmol) was added, and the reaction stirred for 1 h. The product was precipitated by dropwise addition of water (15 mL) and filtered. The precipitate was washed with MeOH (3 x 3 mL), Et_2O (3 x 3 mL) and petrol (3 x 3 mL), then dried *in vacuo* to give the desired product as an off-white powder (110 mg, 0.201 mmol, 71%).

m.p. 184.5-188.0 ˚C

IR (ATR) / cm^{-1}: 3278, 3084, 1739, 1697, 1625, 1591, 1531

^{1}H NMR (400 MHz, DMSO-d_6) δ 10.34 (s, 1H; NH), 7.71 (d, *J* = 8.60 Hz, 1H; H_5), 7.53 (d, *J* = 1.96 Hz, 1H; H_8), 7.41 (dd, *J* = 1.96, 8.60 Hz, 1H; H_6), 7.30 - 7.39 (m, 12H; $H_{aromatic}$), 7.23 - 7.29 (m, 3H; $H_{aromatic}$), 6.25 (s, 1H; H_3), 4.73 - 4.77 (m, 2H; OCH_2), 2.89 - 2.94 (m, 2H; SCH_2), 2.39 (s, 3H; CH_3)

^{13}C NMR (101 MHz, DMSO-d_6) δ 160.4, 154.3, 153.6, 152.9, 144.1, 142.9, 129.5, 128.6, 127.4, 126.6, 115.0, 114.7, 112.5, 105.0, 82.5, 78.7, 67.4, 53.1, 20.2, 18.5

MS(ESI): calcd. for $C_{34}H_{27}NO_4{}^{32}SNa$ $[M+Na]^+$ 568.1553, found 568.1549

$Pd(COD)Cl_2$ (10)

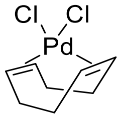

$PdCl_2$ (200 mg, 1 eq., 1.13 mmol) was suspended in concentrated HCl (~37%, 0.5 mL) and gently warmed until fully dissolved. The solution was cooled to room temperature and then diluted with absolute ethanol (15 mL). The solution was filtered, and the residues washed with further ethanol (2 mL). To the filtrate was added 1,5-cyclooctadiene (0.3 mL, 2.16 eq., 2.44 mmol) and the reaction was stirred for 5 min before being left to stand for 10 min. The yellow precipitate was filtered and washed with Et_2O (3 x 3 mL) to give the desired product as a bright yellow powder (216 mg, 0.756 mmol, 67%). The data were in accordance with the literature.[6,7]

^{1}H NMR (400 MHz, $CDCl_3$) δ 6.35 – 6.27 (m, 4H; C$H_{alkenyl}$), 2.98 – 2.85 (m, 4H; CH_{alkyl}), 2.63 – 2.51 (m, 4H; CH_{alkyl})

5-(methylthio)pent-2-yn-1-ol (11a)

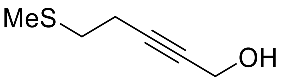

K_2CO_3 (1.77 g, 2.5 eq., 12.8 mmol) was dissolved in water (20 mL) and 4-bromo-1-butyne (750 mg, 1.1 eq., 5.64 mmol) was added. Sodium methanethiolate (359 mg, 1 eq., 5.13 mmol) was then added at room temperature and the reaction stirred for 5 h. The reaction was extracted with Et_2O (3 x 20 mL) and the organics dried over $MgSO_4$, then filtered directly onto 4 Å mol. sieves in a dry flask. The solution was then filtered *via cannula* into a dry flask and chilled to -78 °C. 1.6 M ^{n}BuLi in hexanes (4.81 mL, 1.5 eq., 7.69 mmol) was added dropwise followed by $(CH_2O)_n$ (308 mg, 2 eq., 10.3 mmol) after 10 min. The reaction was warmed to room temperature and stirred for 1 h before quenching with sat. aq. NH_4Cl (30 mL) and separating. The organics were dried over $MgSO_4$, filtered and evaporated. The crude material was purified by flash chromatography on silica (30 g) with 50% Et_2O/petrol to give the desired product as a clear oil (262 mg, 2.01 mmol, 39%) which contains traces of Et_2O and petrol.

R_f (50% Et_2O/Petrol) 0.24

IR (ATR) / cm^{-1}: 3345, 2917, 2865, 1431

^{1}H NMR (500 MHz, $CDCl_3$) δ 4.27 (br. s., 2H; OCH_2), 2.68 (t, *J* = 7.02 Hz, 2H; SCH_2), 2.54 (t, *J* = 6.71 Hz, 2H; CH_2CC), 2.16 (s, 3H; SCH_3), 1.88 (br. s, 1H; O*H*)

^{13}C NMR (126 MHz, $CDCl_3$) δ 84.4, 79.4, 51.2, 33.1, 19.7, 15.6

MS(ESI): calcd. for $C_6H_{11}OS$ $[M+H]^+$ 131.0525, found 131.0530

5-(methylthio)pent-2-yn-1-yl (4-methylcoumarin)carbamate (11)

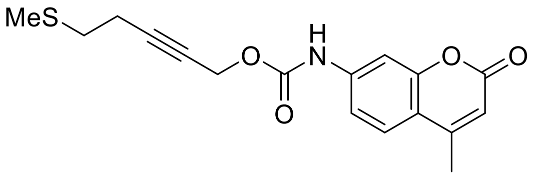

7-amino-4-methylcoumarin (50 mg, 1 eq., 0.285 mmol) and triphosgene (42 mg, 0.5 eq., 0.143 mmol) were dissolved in freshly distilled toluene (10 mL) and refluxed for 0.5 h in oven dried glassware before evaporating *in vacuo*. The crude isocyanate was then dissolved in freshly distilled THF (5 mL) at room temperature and alcohol **11a** (56 mg, 1.5 eq., 0.428 mmol) added followed by dibutyltin dilaurate (0.01 mL, 0.08 eq., 16.9 µmol). After 1 h, the reaction was evaporated *in vacuo* to ~ ½ volume and chilled to 0 °C. Water (10 mL) was added dropwise and the precipitate filtered. The crude solids were triturated with MeOH (2 x 3 mL), Et_2O (2 x 3 mL) and petrol (2 x 3 mL) to give the desired product as a white powder (63 mg, 0.190 mmol, 66%).

m.p. 175.6-177.8 °C

IR (ATR) / cm^{-1}: 3277, 3174, 3100, 2956, 2920, 1735, 1687, 1625

^{1}H NMR (400 MHz, DMSO-d_6) δ 10.33 - 10.37 (m, 1H; N*H*), 7.70 (d, *J* = 8.86 Hz, 1H; H_5), 7.53 (d, *J* = 1.70 Hz, 1H; H_8), 7.40 (dd, *J* = 1.70, 8.52 Hz, 1H; H_6), 6.24 (s, 1H; H_2), 4.80 - 4.82 (m, 2H; OCH_2), 2.59 - 2.65 (m, 2H; SCH_2/CCCH_2), 2.52 - 2.58 (m, 2H; SCH_2/CCCH_2), 2.39 (s, 3H; CCH_3), 2.09 (s, 3H; SCH_3)

^{13}C NMR (101 MHz, DMSO-d_6) δ 160.4, 154.2, 153.6, 153.0, 142.9, 126.5, 115.0, 114.7, 112.5, 104.9, 86.6, 76.0, 53.4, 32.4, 19.4, 18.5, 15.1

MS(ESI): calcd. for $C_{17}H_{18}O_4NS$ $[M+H]^+$ 332.0951, found 332.0960

But-3-yn-1-yl(trityl)sulfane (12a)

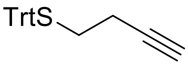

K_2CO_3 (1.5 g, 3 eq., 10.9 mmol) was suspended in DMF (10 mL) in an oven dried flask. 4-bromo-1-butyne (577 mg, 1.2 eq., 4.34 mmol) was added followed by triphenylmethane thiol (1 g, 1 eq., 3.62 mmol) in one portion. After 6 h stirring at room temperature, the reaction was diluted in Et_2O (100 mL) and washed with water basified to pH > 10 with sat. aq. Na_2CO_3 (2 x 50 mL), then brine (50 mL). The organics were dried over $MgSO_4$, filtered and dry-loaded directly onto silica (5 g). The crude material was purified by flash chromatography on silica (50 g) with 5% Et_2O/petrol to give the desired product as a white powder (886 mg, 2.73 mmol, 75%).

R_f 0.41 (5% Et_2O/petrol)

m.p. 73.5-76.5 °C

IR (ATR) / cm^{-1}: 3292, 3057, 3030, 2932, 1594, 1488, 1443

^{1}H NMR (400 MHz, $CDCl_3$) δ 7.46 (d, *J* = 7.32 Hz, 6H), 7.32 (t, *J* = 7.32 Hz, 6H), 7.22 - 7.29 (m, 3H), 2.41 (t, *J* = 7.45 Hz, 2H), 2.15 (dt, *J* = 2.55, 7.45 Hz, 2H), 2.00 (t, *J* = 2.55 Hz, 1H)

^{13}C NMR (101 MHz, $CDCl_3$) δ 144.7, 129.6, 127.9, 126.7, 82.6, 69.2, 66.8, 30.8, 18.5

MS(ESI): calcd. for $C_{23}H_{19}S$ $[M\text{-}H]^-$ 327.1207, found 327.1205

5-(tritylthio)pent-2-yn-1-ol (12b)

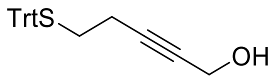

Propargyl ether **12a** (645 mg, 1 eq., 1.96 mmol) was dissolved in freshly distilled THF (20 mL) in an oven dried flask and chilled to -78 °C. 1.6 M ^{n}BuLi in hexanes (1.5 mL, 1.2 eq., 2.36 mmol) was then added dropwise. After 10 mins, $(CH_2O)_n$ (88 mg, 1.5 eq., 2.95 mmol) was then added in one portion and the reaction warmed to room temperature. After 1 h the reaction was quenched with sat. aq. NH_4Cl (30 mL) and the phases separated. The aqueous layer was further extracted with EtOAc (2 x 30 mL). The organics were dried over $MgSO_4$, filtered and evaporated directly onto silica (5 g) and purified by flash chromatography on silica (50 g) with a gradient of 25-50% EtOAc/petrol to give the desired product as a yellow amorphous solid (677 mg, 1.89 mmol, 99%).

R_f 0.33 (40% EtOAc/petrol)

m.p. 99.2-101.5 °C

IR (ATR) / cm^{-1}: 3365, 3057, 3018, 2929, 2870, 1594, 1488, 1443

^{1}H NMR (400 MHz, $CDCl_3$) δ 7.43 (d, *J* = 7.40 Hz, 6H; $H_{aromatic}$), 7.30 (t, *J* = 7.40 Hz, 6H; $H_{aromatic}$), 7.23 (t, *J* = 7.40 Hz, 3H; $H_{aromatic}$), 4.19 - 4.23 (m, 2H; OCH_2), 2.37 (t, *J* = 7.50 Hz, 2H; SCH_2), 2.18 (tt, *J* = 1.70, 7.20 Hz, 2H; CH_2CC), 1.58 - 1.64 (m, 1H; O*H*)

^{13}C NMR (101 MHz, $CDCl_3$) δ 144.7, 129.6, 127.9, 126.7, 84.5, 79.3, 66.8, 51.3, 30.9, 18.8

MS(ESI): calcd. for $C_{24}H_{22}OSNa$ $[M+Na]^+$ 381.1284, found 381.1293

5-(tritylthio)pent-2-yn-1-yl (4-methylcoumarin)carbamate (12)

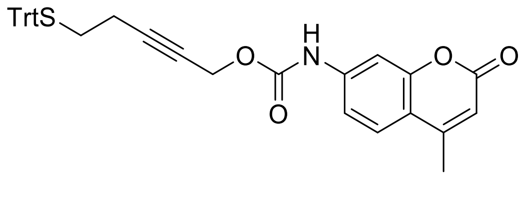

7-amino-4-methylcoumarin (50 mg, 1 eq., 0.285 mmol) and triphosgene (42 mg, 0.5 eq., 0.143 mmol) were dissolved in freshly distilled toluene (10 mL) and refluxed for 0.5 h in oven dried glassware before evaporating *in vacuo*. The crude isocyanate was then dissolved in freshly distilled THF (5 mL) at room temperature and alcohol **12b** (123 mg, 1.2 eq., 0.342 mmol) added followed by dibutyltin dilaurate (0.01 mL, 0.08 eq., 16.9 µmol). After 1 h, the reaction was evaporated *in vacuo* to ~ ½ volume and chilled to 0 °C. Water (10 mL) was added dropwise and the precipitate filtered. The crude solids were triturated with MeOH (2 x 3 mL), Et_2O (2 x 3 mL) and petrol (2 x 3 mL) to give the desired product as an off-white powder (63 mg, 0.191 mmol, 67%).

m.p. 188.8-190.0 °C

IR (ATR) / cm^{-1}: 3277, 3176, 3085, 3057, 2926, 2956, 2854, 1738, 1697, 1624

^{1}H NMR (400 MHz, DMSO-d_6) δ 10.33 (s, 1H; N*H*), 7.69 (d, *J* = 8.86 Hz, 1H; H_5), 7.53 (d, *J* = 2.04 Hz, 1H; H_8), 7.40 (dd, *J* = 1.87, 8.69 Hz, 1H; H_6), 7.30 - 7.36 (m, 12H; H_{trityl}), 7.22 - 7.27 (m, 3H; H_{trityl}), 6.25 (s, 1H; H_2), 4.78 (s, 2H; OCH_2), 2.39 (s, 3H; CCH_3), 2.18 - 2.28 (m, 4H; SCH_2/$CCCH_2$)

^{13}C NMR (101 MHz, DMSO-d_6) δ 160.5, 154.2, 153.6, 153.0, 144.7, 142.9, 129.5, 128.5, 127.2, 126.5, 115.0, 114.7, 112.5, 105.0, 85.9, 76.2, 66.7, 53.3, 30.8, 18.5, 18.4

MS(ESI): calcd. for C35H29NO4S $[M+Na]^+$ 582.1715, found 582.1709

2-(2-(2-ethoxyethoxy)ethoxy)ethyl 4-methylbenzenesulfonate (13a)

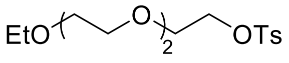

Triethylene glycol monoethylether (25 mL, 1 eq., 143 mmol) was dissolved in TEA (100 mL, 5 eq., 720 mmol) and chilled to 0 °C. TsCl (30 g, 1.1 eq., 157 mmol) was added over 5 min and the reaction allowed to warm to room temperature after 15 min and stirred for 5 h. The reaction mixture was poured slowly over a mixture of ice (200 mL) and conc. HCl (150 mL) and then extracted with Et_2O (3 x 100 mL). The combined organics were washed with brine (100 mL) and sat. aq. $NaHCO_3$ (100 mL), dried over $MgSO_4$, filtered and evaporated to give the desired product as a light yellow oil (42.759 g, 129 mmol, 90%). The data were in agreement with the literature.[8]

^{1}H NMR (400 MHz, $CDCl_3$) δ 7.79 (d, J = 8.05 Hz, 2H; H_{aryl}), 7.34 (d, J = 8.05 Hz, 2H; H_{aryl}), 4.14 - 4.17 (m, 2H; SO_2CH_2), 3.66 - 3.70 (m, 2H; $SO_2CH_2CH_2$), 3.54 - 3.63 (m, 8H; OCH_2CH_2O), 3.51 (q, J = 7.06 Hz, 2H; OCH_2CH_3), 2.44 (s, 3H; $ArCH_3$), 1.20 (t, J = 7.06 Hz, 3H; OCH_2CH_3)

S-(2-(2-(2-ethoxyethoxy)ethoxy)ethyl) ethanethioate (13b)

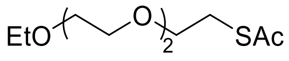

Tosylate **12a** (2 g, 1 eq., 6.02 mmol) was dissolved in acetone (50 mL) and degassed by five evac./refill cycles. KSAc (1.37 g, 2 eq., 12.0 mmol) was then added in one portion and the reaction stirred for 20 h. The reaction was evaporated then diluted in sat. aq. $NaHCO_3$ (60 mL) and extracted with DCM (4 x 30 mL) and evaporated *in vacuo*. This gave the desired product as an orange oil (1.22 g, 5.17 mmol, 86%) which was used crude without further purification.

^{1}H NMR (400 MHz, $CDCl_3$) δ 3.55 - 3.65 (m, 10H; OCH_2), 3.51 (q, J = 7.06 Hz, 2H; OCH_2CH_3), 3.08 (t, J = 6.50 Hz, 2H; CH_2SAc), 2.32 (s, 3H; SAc), 1.19 (t, J = 7.06 Hz, 3H; OCH_2CH_3)

4-chlorobut-2-yn-1-ol (13c)

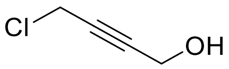

Propargyl chloride (0.2 mL, 1 eq., 2.68 mmol) was dissolved in freshly distilled Et_2O (10 mL) and chilled to -78 ˚C in an oven dried flask. 2.5 M ^{n}BuLi in hexanes (0.96 mL, 0.91 eq., 2.44 mmol) was then added quickly dropwise. After 10 min, $(CH_2O)_n$ (96 mg, 1.2 eq., 3.22 mmol) was added in one portion. The reaction was warmed to room temperature and stirred for 1 h before pouring over saturated aqueous NH_4Cl (20 mL) and extracting with Et_2O (3 x 20 mL). The organics were dried over $MgSO_4$ and evaporated *in vacuo* to give the desired product as an orange oil (209 mg, 2.00 mmol, 72%). The data were in accordance with the literature.[9]

R$_f$ (1% MeOH/DCM): 0.27

^{1}H NMR (400 MHz, $CDCl_3$) δ 4.23 - 4.26 (m, 2H), 4.13 (t, J = 1.92 Hz, 2H)

3,6,9-trioxa-12-thiahexadec-14-yn-16-ol (13d)

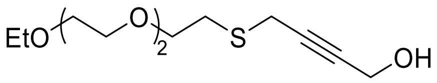

A 1 M solution of NaOMe in MeOH was prepared by dissolving Na (553 mg, 24.1 mmol) in freshly distilled MeOH (24 mL). An portion of this solution (6.3 mL, 2.06 eq., 6.28 mmol) was added to thioester **13b** (721 mg, 1 eq., 3.05 mmol) in an oven dried flask and the reaction stirred at room temperature for 1.5 h. Propargyl alcohol **13c** (478 mg, 1.5 eq., 4.58 mmol) was then added in one portion and the reaction stirred for a further 20 h. The reaction was then evaporated and purified by flash chromatography with a gradient of 50-70% EtOAc/petrol on silica (80 g) to give the desired product as an orange oil (581 mg, 2.21 mmol, 73%).

R$_f$ 0.10 (50% EtOAc/petrol)

IR (ATR) / cm^{-1}: 3407, 2866

^{1}H NMR (500 MHz, $CDCl_3$) δ 4.29 - 4.31 (m, 2H; OC*H*$_2$C≡C), 3.74 (t, J = 6.71 Hz, 2H; SCH$_2$C*H*$_2$O), 3.66 - 3.69 (m, 6H; OC*H*$_2$), 3.60 - 3.63 (m, 2H; C*H*$_2$OCH$_2$CH$_3$), 3.55 (q, J = 7.02 Hz, 2H; OC*H*$_2$CH$_3$), 3.39 (t, J = 1.98 Hz, 2H; SC*H*$_2$C≡C), 2.91 (t, J = 6.71 Hz, 2H; SC*H*$_2$CH$_2$O), 1.89 - 1.95 (m, 1H; O*H*), 1.24 (t, J = 7.02 Hz, 3H; OCH$_2$C*H*$_3$)

^{13}C NMR (126 MHz, $CDCl_3$) δ 81.7, 81.6, 70.8, 70.6, 70.6, 70.4, 69.8, 66.7, 51.1, 30.7, 20.1, 15.1

MS(ESI): calcd. $C_{12}H_{22}O_4S$ for $[M+H]^+$ 263.1259, 263.1256

3,6,9-trioxa-12-thiahexadec-14-yn-16-yl (4-methylcoumarin)carbamate (13)

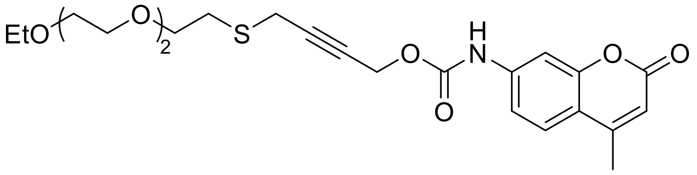

7-amino-4-methylcoumarin (50 mg, 1 eq., 0.285 mmol) and triphosgene (42 mg, 0.5 eq., 0.143 mmol) were dissolved in freshly distilled toluene (10 mL) and refluxed for 0.5 h in oven dried glassware before evaporating *in vacuo*. The crude isocyanate was then dissolved in freshly distilled THF (5 mL) at room temperature and alcohol **13d** (97 mg, 1.3 eq., 0.371 mmol) was added followed by dibutyltin dilaurate (0.01 mL, 0.06 eq., 17.1 µmol). After 1 h, the reaction was evaporated *in vacuo*. The crude viscous oil was treated with petrol (15 mL) and then evaporated again. The crude waxy solid was triturated with petrol (5 x 3 mL) and Et_2O (5 x 3 mL) then dried *in vacuo* to give the desired product as a light brown waxy solid (100 mg, 0.216 mmol, 87%).

IR (ATR) / cm^{-1}: 3278, 3173, 3094, 2868, 17.36, 1687

^{1}H NMR (400 MHz, DMSO-d_6) d 10.37 (s, 1H; N*H*), 7.71 (d, J = 8.69 Hz, 1H; H_5), 7.54 (d, J = 1.36 Hz, 1H; H_8), 7.41 (dd, J = 1.36, 8.69 Hz, 1H; H_6), 6.25 (s, 1H; H_3), 4.86 (s, 2H; $OCH_2C≡C$), 3.61 (t, J = 6.47 Hz, 2H; SCH_2CH_2O), 3.37 - 3.54 (m, 12H; $O(CH_2)_2O$+$SCH_2C≡C$+OCH_2CH_3), 2.78 (t, J = 6.47 Hz, 2H; SCH_2CH_2O), 2.39 (s, 3H; $ArCH_3$), 1.09 (t, J = 6.98 Hz, 3H; OCH_2CH_3)

^{13}C NMR (101 MHz, DMSO-d_6) δ 160.4, 154.3, 153.6, 153.0, 142.9, 126.5, 115.0, 114.7, 112.5, 105.0, 84.2, 77.6, 70.3, 70.3, 70.2, 70.0, 69.6, 66.0, 53.3, 30.9, 19.4, 18.5, 15.6

MS(ESI): calcd. for $C_{23}H_{30}NO_7S$ $[M+H]^+$ 464.1743, found 464.1752

3,6,9-trioxa-12-thiahexadec-14-yn-16-yl (4-nitrophenyl) carbonate (15a)

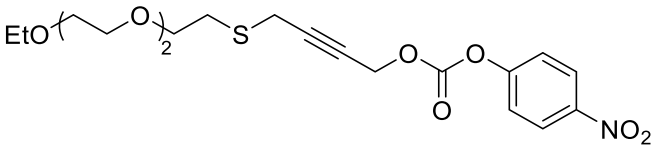

Alcohol **13d** (200 mg, 1 eq., 0.762 mmol) was dissolved in freshly distilled DCM (12 mL) in dry glassware and p-nitrophenylchloroformate (231 mg, 1.5 eq., 1.14 mmol) was added. DIPEA (0.27 mL, 2.0 eq., 1.52 mmol) was added dropwise and the reaction stirred for 20 h. The reaction was evaporated and purified by flash chromatography with 50% EtOAc/petrol on silica (60 g). Crude fractions were evaporated, the material dissolved in DCM (50 mL) and washed with dilute aq. $NaHCO_3$ (30 mL). The aqueous layer was extracted with DCM (25 mL) and the combined organics dried over $MgSO_4$, filtered and evaporated *in vacuo* to give the desired product as a light yellow oil (192 mg, 0.449 mmol, 59%).

R_f 0.3 (50% EtOAc/Petrol)

IR (ATR) / cm^{-1}: 2869, 1768

^{1}H NMR (400 MHz, $CDCl_3$) δ 8.30 (d, J = 9.20 Hz, 2H; $H_{aromatic}$), 7.42 (d, J = 9.20 Hz, 2H; $H_{aromatic}$), 4.92 (t, J = 1.70 Hz, 2H; $OCH_2C≡C$), 3.73 (t, J = 6.56 Hz, 2H; SCH_2CH_2O), 3.63 - 3.69 (m, 6H; OCH_2), 3.58 - 3.63

(m, 2H; CH_2OCH_2CH_3), 3.53 (q, J = 7.07 Hz, 2H; OCH_2CH_3), 3.41 (t, J = 1.87 Hz, 2H), 2.88 (t, J = 6.56 Hz, 2H; SCH_2CH_2O), 1.22 (t, J = 7.07 Hz, 3H; OCH_2CH_3)

^{13}C NMR (101 MHz, $CDCl_3$) δ 155.3, 152.0, 145.5, 125.3, 121.8, 85.2, 75.2, 70.8, 70.7, 70.6, 70.4, 69.8, 66.6, 57.1, 31.1, 20.0, 15.2

MS(ESI): calcd. for $C_{19}H_{26}NO_8S$ $[M+H]^+$ 428.1374, found 428.1360

cDox (15)

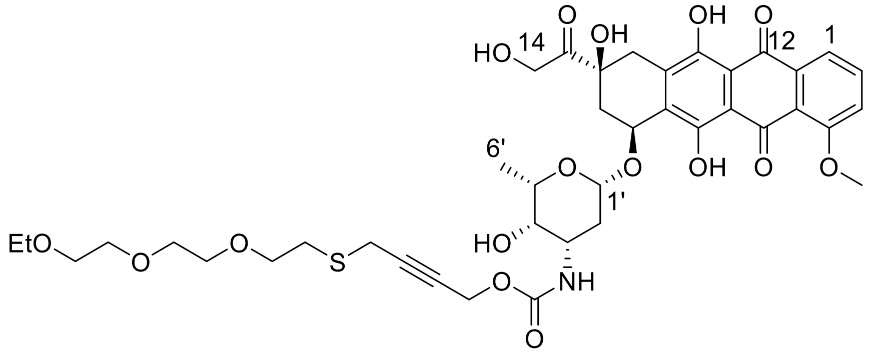

Carbonate **15a** (48 mg, 1.3 eq., 112 µmol) was dissolved in DMF and Doxorubicin hydrochloride (50 mg, 1.0 eq., 86.2 µmol) was added. DIPEA (37 µL, 2.5 eq., 216 µmol) was then added in one portion and the reaction stirred for 3 h then diluted with EtOAc (75 mL), washed with H_2O (2 x 30 mL) and brine (30 mL). The organics were dried over $MgSO_4$, filtered and evaporated *in vacuo*. The crude material was purified by flash chromatography with 5% MeOH/DCM on silica (30 g) to give the desired product as a red waxy solid (55 mg, 65.6 µmol, 76%).

R_f 0.38 (5% MeOH/DCM)

IR (ATR) / cm^{-1}: 3441, 2931, 1721

^{1}H NMR (500 MHz, $CDCl_3$) δ 13.98 (s, 1H; $H_{6/11}$), 13.25 (s, 1H; $H_{6/11}$), 8.05 (d, J = 7.63 Hz, 1H; H_1), 7.80 (dd, J = 7.63, 8.24 Hz, 1H; H_2), 7.41 (d, J = 8.24 Hz, 1H; H_3), 5.52 – 5.48 (m, 1H; $H_{1'}$), 5.43 (d, J = 8.54 Hz, 1H; NH), 5.31 – 5.27 (m, 1H; H_7), 4.78 (d, J = 3.05 Hz, 2H; H_{14}), 4.68 (d, J = 15.4 Hz, 1H; H_9), 4.59 (d, J = 13.9 Hz, 2H; OCH_2C≡C), 4.12 (q, J = 6.7 Hz, 1H; $H_{5'}$), 4.10 (s, 3H; OCH_3), 3.84 - 3.92 (m, 1H; $H_{3'}$),3.63 - 3.73 (m, 10H; SCH_2CH_2O + CH_2O + $H_{4'}$ + $H_{3'}$), 3.59 - 3.63 (m, 2H; CH_2OCH_2CH_3), 3.54 (q, J = 7.02 Hz, 2H; OCH_2CH_3), 3.34 (br. s., 2H; C≡CCH_2S), 3.31 (d, J = 2.2 Hz, 2H; $C_{4'/9}$OH), 3.27 (dd, J = 18.9, 2.0 Hz, 1H; H_{10}), 3.02 (s, 2H; C_{14}OH), 2.97 (d, J = 14.6 Hz, 1H; H_{10}), 2.85 (dt, J = 2.14, 6.70 Hz, 2H; SCH_2CH_2O), 2.50 - 2.55 (m, 1H; $C_{4'/9}$OH), 2.36 (d, J = 14.65 Hz, 1H; H_8), 2.19 (dd, J = 3.66, 14.65 Hz, 1H; H_8), 1.82 - 1.87 (m, 2H; $H_{2'}$), 1.31 (d, J = 6.41 Hz, 3H; $C_{6'}$-CH_3), 1.22 (t, J = 7.02 Hz, 3H; OCH_2CH_3)

^{13}C NMR (126 MHz, $CDCl_3$) δ 213.9, 187.1, 186.7, 161.1, 156.2, 155.7, 154.8, 135.8, 135.5, 133.6, 133.6, 120.9, 119.9, 118.5, 111.6, 111.4, 100.7, 82.7, 77.6, 76.6, 70.7, 70.7, 70.5, 70.3, 69.7, 69.6, 69.3, 67.4, 66.7, 65.6, 56.7, 53.0, 47.2, 35.6, 34.0, 31.5, 30.0, 20.0, 16.9, 15.1

MS(ESI): calcd. for $C_{40}H_{49}NO_{16}SNa$ $[M+Na]^+$ 854.2664, found 854.2648

tert-butyl (2-(2-(2-(2-hydroxyethoxy)ethoxy)ethoxy)ethyl)carbamate (16a)

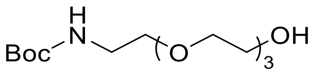

A solution of Boc_2O (207 mg, 1.5 eq., 1.13 mmol) in EtOH (0.5 mL) was added to 2-(2-2-2-aminoetriethoxy)ethanol (150 mg, 1.0 eq., 0.753 mmol) in EtOH (1.0 mL). The reaction mixture was stirred at room temperature for 18 h and the solvent was evaporated *in vacuo*. The residue was dissolved in DCM (20 mL) and washed with 1 M HCl (20 mL), sat. aq. $NaHCO_3$ (20 mL) and brine (20 mL). The organic layer was dried over $MgSO_4$, filtered and evaporated *in vacuo*. The crude material was purified by flash chromatography on silica with 0-10% MeOH/DCM to give the desired product as a pale yellow oil (186 mg, 0.473 mmol, 63%). The data were in accordance with the literature.[8]

R_f (5% MeOH/DCM) 0.41

^{1}H NMR (400 MHz, $CDCl_3$) δ 3.75 – 3.68 (m, 4H; OCH_2CH_2O), 3.68 – 3.60 (m, 8H; OCH_2CH_2O), 3.53 (dd, *J* = 5.5, 4.5 Hz, 2H; OCH_2CH_2N), 3.31 (dd, *J* = 5.5, 4.5 Hz, 2H; CH_2NH), 1.44 (s, 9H; C(CH_3)$_3$)

2,2-dimethyl-4-oxo-3,8,11,14-tetraoxa-5-azahexadecan-16-yl 4-methylbenzenesulfonate (16b)

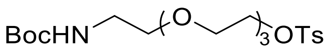

Carbamate **16a** (180 mg, 1.0 eq., 0.617 mmol) was dissolved in freshly distilled DCM (1.0 mL) in an oven dried flask. TEA (422 µL, 5.0 eq., 3.05 mmol) and DMAP (3.66 mg, 0.05 eq., 0.0305 mmol) were added and the reaction was cooled to 0 °C. TsCl (173 mg, 1.5 eq., 0.926 mmol) was added slowly, the reaction allowed to warm to room temperature and stirred for 20 h. Then the reaction was diluted with DCM (20 mL) and washed with HCl 1 M (20 mL). The aqueous layer was extracted with DCM (3 x 20 mL). The organic layers were combined, washed with brine (50 mL), dried over $MgSO_4$, filtered and evaporated *in vacuo*. The crude product was purified by flash chromatography on silica with 0-5% MeOH/DCM to give the desired product as light yellow oil (257 mg, 0.574 mmol, 93 %). The data were in accordance with the literature.[8]

R_f 0.46 (5% MeOH/DCM)

^{1}H NMR (400 MHz, $CDCl_3$) δ 7.80 (d, *J* = 8.3 Hz, 2H; H_{aryl}), 7.34 (d, *J* = 8.1 Hz, 2H; H_{aryl}), 4.16 (dd, *J* = 9.7, 1.1 Hz, 2H; SO_2CH_2), 3.70 (dd, *J* = 5.6, 4.1 Hz, 2H; SO_2CH_2CH_2), 3.60 (m, *J* = 1.7 Hz, 8H; OCH_2CH_2O), 3.53 (t, *J* = 5.2 Hz, 2H; OCH_2CH_2N), 3.30 (q, *J* = 5.5 Hz, 2H; CH_2NH), 2.45 (s, 3H; ArCH_3), 1.44 (s, 9H; C(CH_3)$_3$)

***S*-(2,2-dimethyl-4-oxo-3,8,11,14-tetraoxa-5-azahexadecan-16-yl) ethanethioate (16c)**

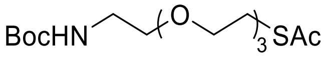

Tosylate **16b** (182 mg, 1.0 eq., 0.407 mmol) was dissolved in acetone (6 mL) and degassed by five evac./refill cycles. KSAc (278 mg, 6.0 eq., 2.44 mmol) was then added in one portion and the reaction stirred for 20 h at ambient temperature. The reaction was then evaporated *in vacuo*, the crude

material diluted in sat. aq. $NaHCO_3$ (30 mL) and extracted with DCM (3 x 30 mL). The organic layers were combined, washed with 5% NaOH (2x 50 mL) and evaporated *in vacuo*. The crude was purified by flash chromatography on silica with 5% MeOH/DCM to give the desired product as a yellow oil (139 mg, 0.394 mmol, 97%).

R_f (5% MeOH/DCM) 0.46

^{1}H NMR (400 MHz, $CDCl_3$) δ 3.66 – 3.57 (m, 10H, OCH_2CH_2O), 3.54 (t, *J* = 5.1 Hz, 2H, OCH_2CH_2N), 3.31 (q, *J* = 5.4 Hz, 2H, CH_2NH), 3.09 (t, *J* = 6.5 Hz, 2H, CH_2SCH_3), 2.33 (s, 3H, CH_2SCH_3), 1.43 (s, 9H, $C(CH_3)_3$)

^{13}C NMR (75 MHz, DMSO-d_6) δ 195.6, 156.1, 70.7, 70.7, 70.5, 70.4, 69.9, 40,5, 30.7, 29.0, 28.6

IR (ATR) / cm^{-1}: 3354, 2977, 2869, 1690, 1513

MS(ESI): calcd. for $C_{15}H_{29}NO_6SNa$ $[M+Na]^+$ 374.1613, found 374.1599

***tert*-butyl (16-hydroxy-3,6,9-trioxa-12-thiahexadec-14-yn-1-yl)carbamate (16d)**

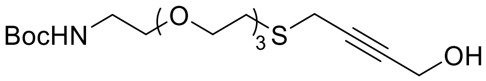

Thioester **16c** (120 mg, 1.0 eq., 0.341 mmol) was dissolved in freshly distilled MeOH (2 mL). A 1 M solution of NaOMe in MeOH was prepared by dissolving Na (553 mg, 24.05 mmol) in freshly distilled MeOH (24 mL). A portion of this solution (0.59 mL, 2.0 eq., 0.683 mmol) was added to **16c** in an oven dried flask and the reaction stirred for 1.5 h. **13c** (48 mg, 1.35 eq., 0.460 mmol) was then added in one portion and the reaction stirred for further 20 h. The reaction was then evaporated *in vacuo* and purified by flash chromatography on silica with 0-33% MeOH/DCM to give the desired product as a yellow oil (109 mg, 0.290 mmol, 85%).

R_f 0.40 (5% MeOH/DCM)

^{1}H NMR (400 MHz, $CDCl_3$) δ 5.18 (s, 1H, O*H*), 4.29 – 4.27 (m, 2H, $OCH_2C{\equiv}C$), 3.74 (t, *J* = 6.5 Hz, 2H, SCH_2CH_2O), 3.68 – 3.60 (m, 8H, OCH_2CH_2O), 3.55 (q, *J* = 5.1 Hz, 2H, OCH_2CH_2N), 3.38 (t, *J* = 2.2 Hz, 2H, $SCH_2C{\equiv}C$), 3.36 – 3.28 (m, 2H, CH_2NH), 2.88 (t, *J* = 6.5 Hz, 2H, CH_2SCH_2), 1.45 (s, 9H, $C(CH_3)_3$)

^{13}C NMR (75 MHz, DMSO-d_6) δ 156.3, 81.8, 81.7, 79.4, 71.0, 70.8, 70.7, 70.4, 66.0, 51.2, 40.5, 31.0, 28.6, 20.3, 15.4

IR (ATR) / cm^{-1}: 3368, 2868, 1691, 1515

MS(ESI): calcd. for $C_{17}H_{31}NO_6S$ $[M+H]^+$ 378.1945, found 378.1941

(*E*)-*N*-(16-hydroxy-3,6,9-trioxa-12-thiahexadec-14-yn-1-yl)-4-oxo-4-phenylbut-2-enamide (16e)

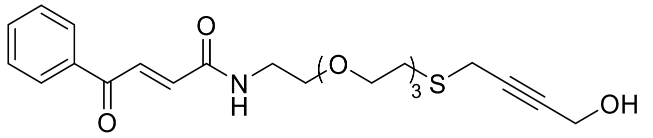

Alcohol **16d** (60 mg, 1.0 eq., 0.159 mmol) was dissolved in 20% TFA in DCM (1 mL) and stirred for 3 h. Afterwards the solvent was evaporate *in vacuo* and the crude product was dissolved in dry DMF (0.75 mL) in an oven dried flask. DIPEA (31 µL, 2.0 eq, 0.32 mmol) was added to the solution. 3-benzoylacrylic acid (28 mg, 1.0 eq., 0.16 mmol) and HBTU (72 mg, 1.2 eq., 0.19 mmol) were dissolved in dry DMF (0.75 mL) and DIPEA (31 µL, 2.0 eq., 0.318 mmol) was added. This solution was stirred for 15 min and added dropwise to the crude amine in DMF at 0 °C. The reaction was allowed to warm to room temperature and it stirred for 18 h. The solvent was evaporated *in vacuo* and coevaporated 3 times with toluene. The crude product was dissolved in DCM (20 mL) and washed with HCl 1 M (3x 20 mL), $NaHCO_3$ sat. (3x 20 mL) and brine (3x 20 mL). The organic layer was dried over $MgSO_4$, filtered and evaporated *in vacuo*. The crude material was purified by flash chromatography on silica with 0-5% MeOH/DCM to give the desired product as brown waxy oil (15.1 mg, 35.2 µmol, 22% over two steps).

R_f 0.26 (5% MeOH/DCM)

^{1}H NMR (400 MHz, $CDCl_3$) δ 8.06 – 8.03 (m, 2H; H_{aryl}), 7.98 (d, *J* = 15.1 Hz, 1H; *H*C=CH), 7.65 – 7.59 (m, 1H; H_{aryl}), 7.51 (dd, *J* = 8.4, 7.0 Hz, 2H; H_{aryl}), 7.31 (s, 1H; N*H*), 7.04 (d, *J* = 15.1 Hz, 1H; HC=C*H*), 4.28 (t, *J* = 2.1 Hz, 2H; OCH_2C≡C), 3.74 (t, *J* = 6.6 Hz, 2H; SCH_2CH_2O), 3.71 – 3.64 (m, 10H; OCH_2CH_2O), 3.62 (dd, *J* = 7.4, 3.3 Hz, 2H; SCH_2C≡C), 3.32 (t, *J* = 2.1 Hz, 2H; CH_2NH), 2.86 (t, *J* = 6.6 Hz, 2H; CH_2SCH_2), 2.39 (s; O*H*)

^{13}C NMR (75 MHz, DMSO-*d*$_6$) δ 189.9, 164.4, 136.9, 135.6, 133.7, 133.0, 128.9, 128.8, 81.8, 81.4, 70.8, 70.6, 70.5, 70.2, 70.1, 69.6, 51.0, 39.9, 30.8, 20.1

IR (ATR) / cm^{-1}: 3279, 3070, 2917, 2867, 1646, 1596, 1579, 1535

MS(ESI): calcd. for $C_{22}H_{29}NO_6SNa$ $[M+Na]^+$ 458.1608, found 458.1597

(*E*)-*N*-(16-hydroxy-3,6,9-trioxa-12-thiahexadec-14-yn-1-yl)-4-oxo-4-phenylbut-2-enamide doxorubicin carbamate (16)

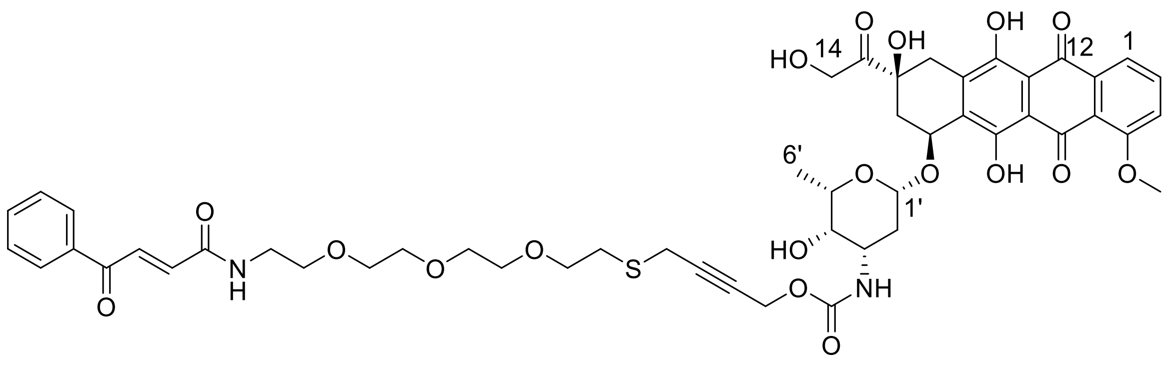

Alcohol **16e** (15 mg, 1.0 eq., 34.4 µmol) was dissolved in DCM (1 mL) and *p*-nitrophenylchloroformate (11 mg, 1.57 eq., 54.1 µmol) was added in dry glassware. DIPEA (18 µL, 3.0 eq., 0.103 mmol) was added dropwise and the reaction stirred for 20 h. Then it was diluted with DCM (20 mL) and washed with dilute aq. $NaHCO_3$ (20 mL). The aqueous layer was extracted with DCM (2 x 20 mL) and the combined organic layer was dried over $MgSO_4$, filtered and evaporated under reduced pressure. The residue was dissolved in dry DMF (1 mL) and Doxorubicin hydrochloride (20 mg, 1.0 eq., 0.035 mmol) and DIPEA (18 µL, 3.0 eq., 0.11 mmol) was then added in one portion and the reaction stirred for 18 h. Afterwards DMF was coevaporated with toluene (3 x 5 mL). The crude product was purified by flash chromatography on silica with 0-5% MeOH/DCM and then purified by preparative TLC (1 mm, 5% MeOH/DCM) to give the desired product as a red waxy solid (15 mg, 15.1 µmol, 43% over two steps).

R_f 0.21 (5% MeOH/DCM)

^{1}H NMR (400 MHz, $CDCl_3$) δ 13.97 (s, 1H, $H_{6/11}$), 13.27 (s, 1H, $H_{6/11}$), 8.08 – 8.00 (m, 3H, $H_{1,}$ H_{Aryl}), 7.96 (d, *J* = 15.0 Hz, 1H, *H*C=CH), 7.79 (dd, *J* = 8.5, 7.7 Hz, 1H, H_{Aryl}), 7.60 (t, *J* = 7.4 Hz, 1H, H_{Aryl}), 7.50 (t, *J* = 7.6 Hz, 2H, H_{Aryl}), 7.39 (dd, *J* = 8.6, 1.1 Hz, 1H, H_{Aryl}), 7.22 (s, 1 H, N*H*), 7.05 (d, *J* = 15.1 Hz, 1H, HC=C*H*), 5.64 (d, *J* = 8.6 Hz, 1H, N*H*), 5.57 – 5.49 (m, 1H, H_1), 5.31 (s, 1H, H_7), 4.77 (s, 2H, H_{14}), 4.70 (s, 1H, H_9), 4.65 (d, *J* = 19.5 Hz, 2H, OCH_2C≡C), 4.50 (d, *J* = 15.4 Hz, 2H), 4.22 (s, 1H, $H_{5'}$), 4.08 (s, 3H, OCH_3), 3.87 (s, 1H, $H_{3'}$), 3.78 – 3.54 (m, 16H, SCH_2CH_2O + CH_2O + $H_{4'}$ + $H_{3'}$), 3.31 (d, *J* = 2.5 Hz, 2H, C≡CCH_2S), 3.27 (d, *J* = 2.0 Hz, 1H, H_{10}), 3.15 (d, *J* = 6.3 Hz, 1H, H_{10}), 2.86 – 2.80 (m, 2H, SCH_2CH_2O), 2.36 (d, *J* = 14.6 Hz, 1H, H_8), 2.22 – 2.12 (m, 1H, H_8), 1.90 – 1.80 (m, 2H, $H_{2'}$), 1.31 (d, *J* = 6.5 Hz, 3H, $C_{6'}$-CH_3)

^{13}C NMR (75 MHz, DMSO-d_6) δ 213.90, 189.70, 187.16, 186.77, 164.48, 161.07, 156.24, 155.72, 154.84, 154.75, 136.92, 135.75, 135.56, 135.37, 133.70, 133.62, 133.09, 128.84, 120.96, 119.86, 118.43, 111.62, 111.44, 100.80, 82.76, 70.86, 70.55, 70.42, 70.26, 70.20, 69.56, 69.30, 67.98, 67.48, 65.57, 56.70, 52.94, 47.33, 39.92, 35.67, 34.03, 30.75, 30.06, 29.71, 25.62, 20.03, 17.00

IR (ATR) / cm^{-1}: 3694, 3327, 2925, 2335, 2191, 2064, 1974,1721, 1646, 1579

MS(ESI): calcd. for $C_{50}H_{56}N_2O_{18}S$ $[M+H]^+$ 1027.3141, found 1027.3108

***tert*-butyl(prop-2-yn-1-yl)sulfane (17a)**

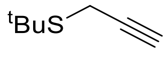

NaOH (106 mg, 1.2 eq., 2.66 mmol) was dissolved in MeOH (4.5 mL) before chilling to 0 ˚C and 80% propargyl bromide in toluene (0.37 mL, 1.5 eq., 3.33 mmol) was added. ^{t}BuSH (0.25 mL, 1 eq., 2.22 mmol) was then added in one portion and the reaction warmed to room temperature. After 3 h stirring, the reaction mixture was evaporated *in vacuo*, the residues diluted in H_2O and extracted with DCM (3 x 20 mL). The organics were dried over $MgSO_4$ and evaporated *in vacuo* to give the desired product as a yellow oil (185 mg, 1.44 mmol, 65%). The data were in accordance with the literature.[10]

^{1}H NMR (400 MHz, $CDCl_3$) δ 3.26 (d, *J* = 2.74 Hz, 2H; CH_2), 2.17 (t, *J* = 2.65 Hz, 1H; C*H*), 1.37 (s, 9H $(CH_2)_3$)

4-(*tert*-butylthio)but-2-yn-1-ol (17b)

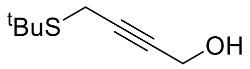

Alkyne **17a** (185 mg, 1 eq., 1.44 mmol) was dissolved in freshly distilled THF (10 mL) and chilled to -78 ˚C in an oven dried flask. 2.5 M ^{n}BuLi in hexanes (0.63 mL, 1.1 eq., 1.59 mmol) was then added dropwise. $(CH_2O)_n$ (65 mg, 1.5 eq., 2.16 mmol) was added after 10 min. The reaction was warmed to room temperature and stirred for 1 h before pouring over saturated aqueous NH_4Cl (20 mL) and extracting with Et_2O (3 x 20 mL). The organics were dried over $MgSO_4$ and evaporated *in vacuo*. The crude material was purified by flash chromatography with 50% Et_2O/petrol on silica (26 g) to give the desired product as a light yellow oil (71 mg, 0.448 mmol, 31%). The data were in accordance with the literature.[11]

R_f (50% Et_2O/petrol) 0.31

^{1}H NMR (400 MHz, $CDCl_3$) δ 4.24 (t, *J* = 2.10 Hz, 2H; OC*H*$_2$), 3.30 (t, *J* = 2.10 Hz, 2H; SC*H*$_2$), 1.35 (s, 9H; (C*H*$_3$)$_3$)

4-(*tert*-butylthio)but-2-yn-1-yl (4-methylcoumarin)carbamate (17)

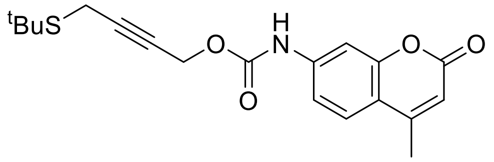

7-amino-4-methylcoumarin (50 mg, 1 eq., 0.285 mmol) and triphosgene (42 mg, 0.5 eq., 0.143 mmol) were dissolved in freshly distilled toluene (10 mL) in and refluxed for 0.5 h in oven dried glassware before evaporating *in vacuo*. The crude isocyanate was then dissolved in freshly distilled THF (5 mL) at room temperature and alcohol **17b** (68 mg, 1.5 eq., 0.428 mmol) added followed by dibutyltin dilaurate (0.01 mL, 0.06 eq., 20.4 µmol). After 1 h, the reaction was chilled to 0 ˚C and water (15 mL) added dropwise to precipitate the product. The precipitate was filtered and washed with MeOH (3 mL), Et_2O (2 x 3 mL) and petrol (3 mL), then dried *in vacuo* to give the desired product as an off-white powder (34 mg, 0.0938 mmol, 33%).

m.p. 175.1-176.3 °C

IR (ATR) / cm^{-1}: 3263, 3058, 2963, 1721, 1686, 1620

^{1}H NMR (400 MHz, DMSO-d$_6$) δ 7.69 (d, *J* = 8.74 Hz, 1H; *H*$_5$), 7.52 (d, *J* = 2.06 Hz, 1H; *H*$_8$), 7.39 (dd, *J* = 2.06, 8.74 Hz, 1H; *H*$_6$), 6.24 (d, *J* = 1.28 Hz, 1H; *H*$_3$), 4.82 (t, *J* = 2.10 Hz, 2H; OC*H*$_2$), 3.44 (t, *J* = 2.10 Hz, 2H; SC*H*$_2$), 2.38 (d, *J* = 1.28 Hz, 3H; ArC*H*$_3$), 1.30 (s, 9H; (C*H*$_3$)$_3$)

^{13}C NMR (126 MHz, DMSO-d$_6$) δ 160.4, 154.3, 153.6, 153.0, 142.9, 126.5, 115.0, 114.7, 112.5, 105.0, 85.2, 77.0, 53.3, 43.5, 30.9, 18.5, 16.4

MS(ESI): calcd. for $C_{19}H_{21}NO_4SNa$ $[M+Na]^+$ 328.1084, found 328.1065

Methyl *N*-(*tert*-butoxycarbonyl)-*S*-(4-hydroxybut-2-yn-1-yl)-*L*-cysteinate (18a)

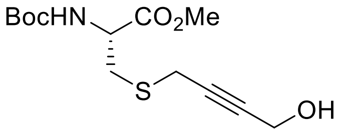

KOAc (1.514 g, 3.5 eq., 15.0 mmol) was suspended in acetone (100 mL) and **13c** (0.67 g, 1.5 eq., 6.44 mmol) added. BocCysOMe (1.01 g, 1 eq., 4.29 mmol) was then added and the reaction stirred at room temperature for 20 h. The reaction was then evaporated *in vacuo*, and purified by flash chromatography with 40% EtOAc/petrol on silica (60 g) to give the desired product as a light orange oil (1.01 g, 3.33 mmol, 78%).

R_f (50% EtOAc/petrol) 0.16

IR (ATR) / cm^{-1}: 3382, 2978, 1742, 1698, 1511

^{1}H NMR (400 MHz, $CDCl_3$) δ 5.41 - 5.53 (m, 1H; N*H*), 4.53 - 4.70 (m, 1H; αC*H*), 4.23 (br. s, 1H; O*H*), 3.74 (s, 3H; CH_3), 3.21 - 3.39 (m, 2H; SCH_2), 2.95 - 3.18 (m, 2H; βCH_2), 1.41 (s, 9H; (CH_3)$_3$)

^{13}C NMR (101 MHz, $CDCl_3$) δ 171.6, 155.3, 82.8, 80.6, 80.4, 53.3, 52.6, 50.8, 33.8, 28.3, 20.2

MS(ESI): calcd. for $C_{13}H_{22}NO_5S$ $[M+H]^+$ 304.1219, found 304.1213

Methyl *N*-(*tert*-butoxycarbonyl)-*S*-(4-(((4-methylcoumarin)carbamoyl)oxy)but-2-yn-1-yl)-*L*-cysteinate (18)

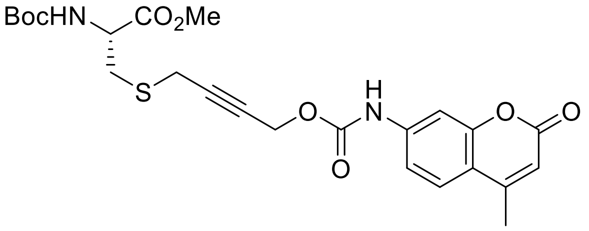

7-amino-4-methylcoumarin (35 mg, 1 eq., 0.200 mmol) and triphosgene (30 mg, 0.5 eq., 0.100 mmol) were dissolved in freshly distilled toluene (10 mL) in and refluxed for 0.5 h in oven dried glassware before evaporating *in vacuo*. The crude isocyanate was then dissolved in freshly distilled THF (3 mL) at room temperature and alcohol **18a** (85 mg, 1.4 eq., 0.280 mmol) in further THF (2 mL) added followed by dibutyltin dilaurate (0.01 mL, 0.08 eq., 16.9 µmol). After 1 h, the reaction was evaporated *in vacuo* and re-dissolved in DCM (10 mL). 10% aqueous KF (10 mL) was added and stirred for 10 min before the layers were separated. The aqueous layer was extracted with DCM (2 x 10 mL) and the organics were dried over $MgSO_4$, filtered and evaporated *in vacuo*. The crude solids were triturated with MeOH (3 x 3 mL), Et_2O (3 x 3 mL) and petrol (3 x 3 mL) to give the desired product as an off-white powder (41 mg, 0.0821 mmol, 41%).

m.p. 137-139 °C

IR (ATR) / cm^{-1}: 3284, 3082, 2986, 1741, 1683, 1624

^{1}H NMR (500 MHz, DMSO-d$_6$) δ 10.39 (s, 1H; N*H*), 7.72 (d, *J* = 8.85 Hz, 1H; *H*$_5$), 7.54 (d, *J* = 1.83 Hz, 1H; *H*$_8$), 7.41 (dd, *J* = 1.83, 8.85 Hz, 1H; *H*$_6$), 6.25 (d, *J* = 0.92 Hz, 1H; *H*$_3$), 4.92 (t, *J* = 1.83 Hz, 1H; OC*H*$_2$), 4.55 (t, *J* = 1.83 Hz, 1H; OC*H*$_2$), 4.12 - 4.17 (m, 1H; αC*H*), 3.64 - 3.66 (m, 3H; CO$_2$C*H*$_3$), 2.78 - 2.95 (m, 2H; SC*H*$_2$), 2.67 - 2.74 (m, 1H; SC*H*$_2$), 2.55 - 2.62 (m, 1H; SC*H*$_2$), 2.40 (s, 3H; ArC*H*$_3$), 1.40 (s, 9H; (C*H*$_3$)$_3$)

^{13}C NMR (126 MHz, DMSO-d$_6$) δ 171.2, 160.0, 155.3, 153.8, 153.2, 152.4, 142.3, 126.1, 114.8, 114.2, 112.0, 104.3, 82.1, 81.2, 78.4, 56.1, 52.4, 51.9, 30.6, 28.1, 25.3, 18.0

MS(ESI): calcd. for $C_{24}H_{28}N_2O_8SNa$ [M+Na]$^+$ 527.1459, found 527.1443

Methyl *S*-(4-hydroxybut-2-yn-1-yl)-*L*-cysteinate (19a)

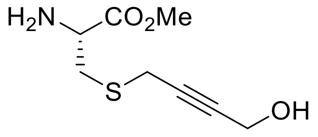

Alcohol **18a** (1.01 g, 1 eq., 3.33 mmol) was dissolved in DCM (10 mL) and TFA (1 mL) added. After 3 h, the reaction was evaporated *in vacuo* and purified by flash chromatography with 5% TEA/8% MeOH/DCM on silica (10 g), then 5% MeOH/EtOAc on silica (30 g) then 8% MeOH/EtOAc on silica (60 g) to give the desired product (542 mg, 2.66 mmol, 80%). The product could not be fully separated from triethylamine salts and was used crude.

R$_f$ (8% MeOH/EtOAc) 0.19

^{1}H NMR (400 MHz, CDCl$_3$) d 4.26 (t, J = 2.20 Hz, 2H; OC*H*$_2$), 3.80 (dd, J = 4.85, 7.23 Hz, 1H; α*H*), 3.76 (s, 3H; C*H*$_3$), 3.32 (td, J = 2.13, 4.35 Hz, 2H; SC*H*$_2$), 3.11 (dd, J = 4.76, 13.90 Hz, 1H; β*H*), 2.98 (dd, J = 7.14, 13.72 Hz, 1H; β*H*), 2.56 (br. s., 3H; O*H*/N*H*$_2$)

Methyl *N*-acetyl-*S*-(4-hydroxybut-2-yn-1-yl)-*L*-cysteinate (19b)

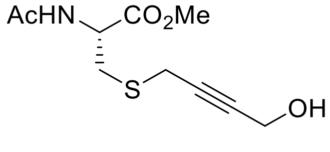

Alcohol **19a** (191 mg, 1 eq., 0.940 mmol) was dissolved in DCM (10 mL) and TEA (0.2 mL, 1.5 eq., 1.41 mmol) was added followed by AcCl (0.08 mL, 1.2 eq., 1.13 mmol). After 4 h the reaction was evaporated *in vacuo* and purified by flash chromatography with 1% HCOOH/EtOAc to give the desired product as a light yellow oil (53 mg, 0.216 mmol, 23%).

R$_f$ (1% HCOOH/EtOAc) 0.33

^{1}H NMR (500 MHz, CDCl$_3$) δ 6.57 (br. d, J = 6.70 Hz, 1H; N*H*), 4.92 - 4.97 (m, 1H; α*H*), 4.28 - 4.31 (m, 2H; OC*H*$_2$), 3.81 (s, 3H; OC*H*$_3$), 3.40 (td, J = 1.98, 16.78 Hz, 1H; β*H*), 3.31 - 3.34 (m, 1H; β*H*), 3.28 - 3.30 (m, 1H; SC*H*), 3.09 (dd, J = 5.65, 14.19 Hz, 1H; SC*H*), 2.10 (s, 3H; COC*H*$_3$)

^{13}C NMR (126 MHz, CDCl$_3$) δ 171.3, 170.9, 83.3, 80.4, 52.9, 52.5, 50.9, 33.1, 23.2, 20.4

IR (ATR) / cm^{-1}: 3275, 2954, 1740, 1655, 1537

MS(ESI): calcd. For $C_{10}H_{16}O_4N^{32}S$ $[M+H]^+$ 246.0795, found 246.0791

Methyl *N*-acetyl-*S*-(4-(((4-methylcoumarin)carbamoyl)oxy)but-2-yn-1-yl)-*L*-cysteinate (19)

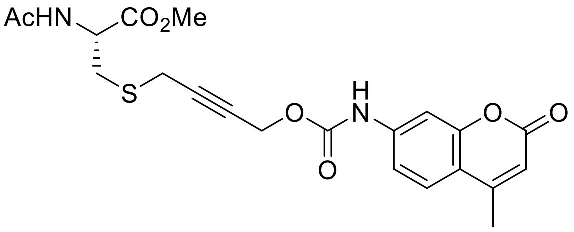

7-amino-4-methylcoumarin (50 mg, 1 eq., 0.285 mmol) and triphosgene (42 mg, 0.5 eq., 0.143 mmol) were dissolved in freshly distilled toluene (10 mL) and refluxed for 0.5 h in oven dried glassware before evaporating *in vacuo*. The crude isocyanate was then dissolved in freshly distilled THF (3 mL) at room temperature and alcohol **19b** (77 mg, 1.1 eq., 0.314 mmol) added in further THF (2 mL) followed by dibutyltin dilaurate (0.01 mL, 0.06 eq., 16.9 µmol). After 1 h, the reaction was evaporated *in vacuo* and re-dissolved in DCM (10 mL). 10% aqueous KF (10 mL) was added and stirred for 10 min before the layers were separated. The aqueous layer was extracted with DCM (2 x 10 mL) and the organics were dried over $MgSO_4$, filtered and evaporated *in vacuo*. The crude solids were triturated with MeOH (3 x 3 mL), Et_2O (3 x 3 mL) and petrol (3 x 3 mL) to give the desired product as a yellow powder (46 mg, 0.103 mmol, 36%).

Decomp. 184 °C

^{1}H NMR (500 MHz, DMSO-d_6) δ 10.35 (br. s, 1H; N*H*CO_2), 8.41 (d, *J* = 7.90 Hz, 1H; N*H*CO), 7.71 (d, *J* = 8.54 Hz, 1H; H_5), 7.53 (d, *J* = 1.83 Hz, 1H; H_8), 7.40 (dd, *J* = 2.14, 8.54 Hz, 1H; H_6), 6.25 (d, *J* = 0.92 Hz, 1H; H_3), 4.85 - 4.87 (m, 2H; OCH_2), 4.47 - 4.56 (m, 1H; α*H*), 3.64 (s, 3H; OCH_3), 3.48 - 3.52 (m, 2H; SCH_2), 3.05 (dd, *J* = 5.34, 13.89 Hz, 1H; β*H*), 2.86 (dd, *J* = 8.70, 13.89 Hz, 1H; β*H*), 2.39 (d, *J* = 0.61 Hz, 3H; ArCH_3), 1.87 (s, 3H; COCH_3)

^{13}C NMR (126 MHz, DMSO-d_6) δ 171.7, 169.9, 160.5, 154.3, 153.6, 153.0, 142.9, 126.5, 115.0, 114.7, 112.5, 105.0, 83.4, 78.1, 53.2, 52.6, 52.0, 32.9, 22.7, 19.4, 18.5

IR (ATR) / cm^{-1}: 3279, 1739, 1687, 1625

MS(ESI): calcd. For $C_{21}H_{23}O_7N_2S$ $[M+H]^+$ 447.1220, found 447.1205

Methyl *S*-(4-hydroxybut-2-yn-1-yl)-*L*-cysteinate hydrochloride (20a)

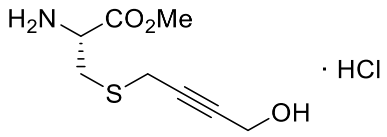

In a dry flask, dry MeOH (10 mL) was chilled to 0 °C and AcCl (0.06 mL) added. The reaction was warmed to room temperature, and then re-chilled to 0 °C before amine **19a** (80 mg, 1 eq., 0.394 mmol) was added. After 5 min the reaction was evaporated *in vacuo* to give the desired product as a clear oil (88 mg, 0.367 mmol, 93%) which was used without further purification.

Methyl *S*-(4-(((4-methylcoumarin)carbamoyl)oxy)but-2-yn-1-yl)-*L*-cysteinate (20)

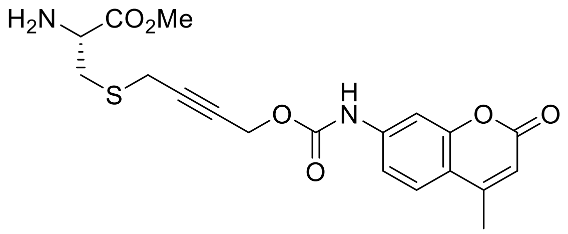

7-amino-4-methylcoumarin (50 mg, 1 eq., 0.285 mmol) and triphosgene (42 mg, 0.5 eq., 0.143 mmol) were dissolved in freshly distilled toluene (10 mL) and refluxed for 0.5 h in oven dried glassware before evaporating *in vacuo*. The crude isocyanate was then dissolved in freshly distilled THF (3 mL) at room temperature and alcohol **20a** (88 mg, 1.29 eq., 0.367 mmol) added in further THF (2 mL) followed by dibutyltin dilaurate (0.01 mL, 0.06 eq., 16.94 µmol). After 1 h, the reaction was evaporated *in vacuo* and re-dissolved in DCM (10 mL). 10% aqueous KF (10 mL) was added and stirred for 10 min before the aqueous layer was basified to pH > 10 with saturated aqueous Na_2CO_3 and the layers were separated. The aqueous layer was extracted with DCM (2 x 10 mL) and the organics were dried over $MgSO_4$, filtered and evaporated *in vacuo*. The crude solids were triturated with MeOH (3 x 3 mL), Et_2O (3 x 3 mL) and petrol (3 x 3 mL) to give the desired product as a dark yellow powder (52 mg, 0.129 mmol, 45%).

m.p. 109-111 °C

^{1}H NMR (500 MHz, DMSO-d_6) δ 9.33 (s, 1H; N*H*CO_2), 7.64 (d, *J* = 8.54 Hz, 1H; H_5), 7.57 (d, *J* = 2.14 Hz, 1H; H_5), 7.26 (dd, *J* = 1.98, 8.70 Hz, 1H; H_8), 6.89 (d, *J* = 7.93 Hz, 1H; H_6), 6.20 (d, *J* = 0.92 Hz, 1H; H_3), 5.16 (t, *J* = 5.95 Hz, 1H; N*H*), 4.56 - 4.64 (m, 1H; N*H*), 4.07 - 4.10 (m, 2H; OCH_2), 3.70 (s, 3H; OCH_3), 3.45 - 3.49 (m, 2H; SCH_2), 3.13 (dd, *J* = 4.88, 13.73 Hz, 1H; β*H*), 3.04 (dd, *J* = 7.32, 13.73 Hz, 1H; β*H*), 2.38 (d, *J* = 0.61 Hz, 3H; CH_3CO)

^{13}C NMR (126 MHz, DMSO-d_6) δ 172.0, 160.6, 154.6, 154.5, 153.7, 144.1, 126.4, 114.4, 114.0, 111.8, 104.3, 83.7, 80.1, 52.8, 52.5, 49.5, 33.4, 19.8, 18.4

IR (ATR) / cm^{-1}: 3381, 2922, 1717, 1677, 1618

MS(ESI): calcd. For $C_{19}H_{21}O_6N_2S$ $[M+H]^+$ 405.1120, found 405.1114

Pd(nbd)Cl$_2$ (21)

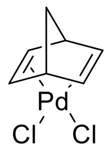

$PdCl_2$ (100 mg, 1 eq., 0.564 mmol) was suspended in concentrated HCl (~37%, 0.25 mL) and gently warmed until fully dissolved. The solution was cooled to room temperature and then diluted with absolute ethanol (7.5 mL). The solution was filtered, and the residues washed with further ethanol (1 mL). To the filtrate was added norbornadiene (0.12 mL, 2.16 eq., 1.22 mmol) and the reaction was stirred for 5 min before being left to stand for 10 min. The yellow precipitate was filtered and washed with Et_2O (3 x 3 mL) to give the desired product as a bright yellow powder (94 mg, 0.349 mmol, 62%).[7,12]

^{1}H NMR (400 MHz, DMSO-d$_6$) δ 6.74 (t, *J* = 1.92 Hz, 4H; C$H_{alkenyl}$), 3.56 – 3.52 (m, 2H; C$H_{bridgehead}$), 1.87 (t, *J* = 1.56 Hz, 2H; C$H_{bridging}$)

Prop-2-yn-1-yl benzylcarbamate (22)

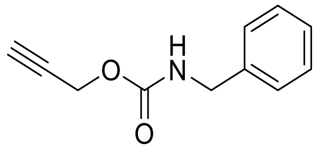

Benzylamine (1.02 mL, 1 eq., 9.33 mmol) was dissolved in freshly distilled THF in an oven dried flask and TEA (1.95 mL, 1.5 eq., 14.0 mmol) was added. Propargyl chloroformate (1.0 mL, 1.1 eq., 10.3 mmol) was added dropwise and the reaction stirred for 1.5 h. The reaction mixture was diluted with icewater (50 mL) and extracted with EtOAc (3 x 80 mL). The organics were washed with icewater/brine (30 mL), dried over $MgSO_4$ and evaporated *in vacuo* to yield the desired product as a white powder (1.652 g, 1.07 mmol, 94%).

m.p. 52.0-55.0 ˚C

IR (ATR) / cm^{-1}: 3288, 3032, 2948, 2878, 1692, 1622, 1519

^{1}H NMR (400 MHz, $CDCl_3$) δ 7.19 - 7.35 (m, 5H; $H_{aromatic}$), 5.21 (br. s, 1H; N*H*), 4.68 (d, *J* = 2.21 Hz, 2H; OCH_2), 4.31 - 4.37 (m, 2H; NCH_2), 2.45 (t, *J* = 2.21 Hz, 1H; C≡C*H*)

^{13}C NMR (126 MHz, $CDCl_3$) δ 155.5, 138.1, 128.7, 127.6, 127.5, 78.2, 74.7, 52.6, 45.2

MS(ESI): calcd. for $C_{11}H_{12}NO_3$ $[M+H]^+$ 190.0868, found 190.0865

Decaging of 22

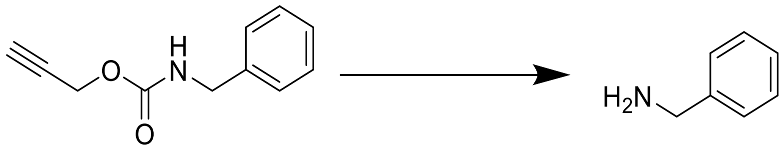

Propargyl carbamate **22** (50 mg, 1 eq., 0.264 mmol) and allylpalladium chloride dimer (10 mg, 0.1 eq., 0.0264 mmol) were dissolved in 10% H_2O/acetone (2 mL) and degassed by three freeze-pump-thaw cycles. The reaction was warmed to room temperature and stirred overnight before evaporating *in vacuo*. The crude material was purified by flash chromatography on silica (29 g) with 3% TEA, 3% MeOH/DCM to give benzylamine (28.5 mg, 0.266 mmol, 101%) as a brown oil.

But-2-yn-1-yl benzylcarbamate (23)

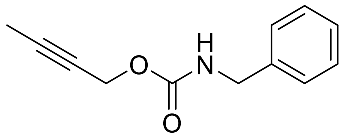

2-butyn-1-ol (0.11 mL, 1 eq., 1.43 mmol) and TEA (0.6 mL, 2.5 eq., 3.58 mmol) were dissolved in freshly distilled ACN (7 mL) at room temperature. DSC (548 mg, 1 eq., 1.43 mmol) was then added and the reaction stirred for 2 h. Benzylamine (0.31 mL, 2.0 eq., 2.85 mmol) was added and the reaction stirred for 15 min. The reaction was diluted with sat. aq. $NaHCO_3$ (30 mL) and extracted with EtOAc (5 x 30 mL). The organics were dried over $MgSO_4$, filtered and evaporated *in vacuo*. The crude product was dry-loaded onto silica (1.4 g) and purified by flash chromatography with 80% petrol/EtOAc on silica (37 g) to yield the product as an off-white waxy solid (217 mg, 1.07 mmol, 75%).

m.p. 52.1-54.1 °C

IR (ATR) / cm^{-1}: 3337, 3030, 2922, 1699, 1517

^{1}H NMR (400 MHz, $CDCl_3$) δ 7.25 - 7.36 (m, 5H; $H_{aromatic}$), 5.07 (br. s, 1H; N*H*), 4.68 (br. q, *J* = 2.39 Hz, 2H; OCH_2), 4.38 (d, *J* = 5.85 Hz, 2H; NCH_2), 1.86 (t, *J* = 2.39 Hz, 3H; C≡CCH_3)

^{13}C NMR (101 MHz, $CDCl_3$) δ 155.8, 138.2, 128.7, 127.6, 127.5, 83.0, 73.7, 53.5, 45.2, 3.7

MS(ESI): calcd. for $C_{12}H_{13}NO_2Na$ $[M+Na]^+$ 226.0839, found 226.0833

Decaging of 23

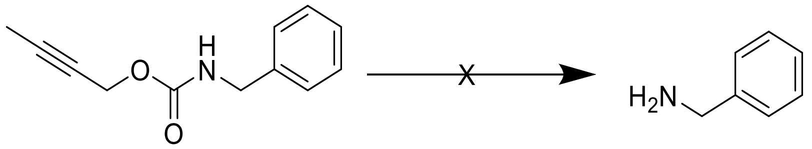

Propargyl carbamate **23** (55 mg, 1 eq., 0.273 mmol) and allylpalladium chloride dimer (10 mg, 0.1 eq., 0.0273 mmol) were dissolved in 10% H_2O/acetone (2 mL) and degassed by four freeze-pump-thaw

cycles. The reaction was warmed to 50 ˚C for 30 min and allowed to cool to room temperature overnight before evaporating *in vacuo*. No benzylamine could be detected by TLC.

4-(tritylthio)but-2-yn-1-yl benzylcarbamate (24)

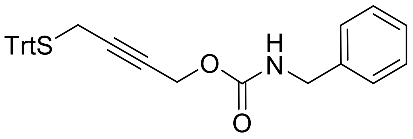

Alcohol **7b** (200 mg, 1 eq., 0.581 mmol) was dissolved in distilled ACN (10 mL) in an oven dried flask and TEA (0.24 mL, 3 eq., 1.74 mmol) added. DSC (223 mg, 1.5 eq., 0.871 mmol) was then added in one portion and the reaction stirred at room temperature for 2 h. $BnNH_2$ (0.12 mL, 2 eq., 1.16 mmol) was then added and the reaction stirred for a further 15 min. The reaction was filtered and evaporated *in vacuo*. The crude material was purified by flash chromatography, with a gradient of petrol to 50% EtOAc/petrol on silica (81 g). A second column was required, with 25% EtOAc/petrol on silica (42 g) to give the desired product as a yellow oil (133 mg, 0.278 mmol, 48%).

R_f (67% EtOAc/petrol) 0.53

IR (ATR) / cm^{-1}: 3330, 3058, 3030, 2937, 1709

^{1}H NMR (400 MHz, $CDCl_3$) δ 7.47 (d, *J* = 7.58 Hz, 6H; $H_{aromatic}$), 7.29 - 7.39 (m, 11H; $H_{aromatic}$), 7.24 - 7.29 (m, 3H; $H_{aromatic}$), 5.16 (br. s, 1H; N*H*), 4.69 (br. s., 2H; OCH_2), 4.40 (d, *J* = 5.87 Hz, 2H; NCH_2), 2.86 - 2.95 (m, 2H; SCH_2)

^{13}C NMR (101 MHz, $CDCl_3$) δ 155.6, 144.0, 138.2, 129.5, 128.7, 128.0, 127.9, 127.6, 127.5, 126.9, 81.9, 77.8, 67.4, 52.9, 45.0, 20.6

MS(ESI): calcd. for $C_{31}H_{27}O_2SNa$ $[M+Na]^+$ 500.1660, found 500.1678

Decaging of 24

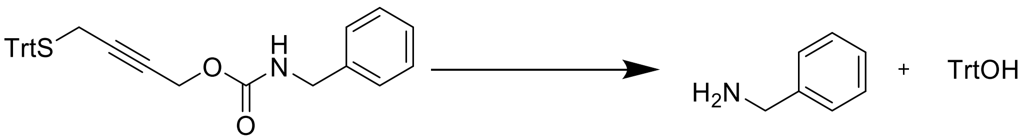

Propargyl carbamate **24** (150 mg, 1 eq., 0.314 mmol) and allylpalladium chloride dimer (23 mg, 0.2 eq., 0.0628 mmol) were dissolved in 10% H_2O/acetone (5 mL) and degassed by four freeze-pump-thaw cycles then warmed to 50 ˚C for 30 min, and allowed to cool to room temperature overnight. The reaction was evaporated *in vacuo*. The crude material was purified by flash chromatography on silica (29 g) with a gradient of DCM to 3% TEA, 3% MeOH/DCM to give triphenylmethanol (90 mg, 0.346 mmol, 110%) as an off-white solid and crude benzylamine. The crude benzylamine was further purified by flash chromatography on silica (19 g) with 5% TEA/EtOAc to give benzylamine (23 mg, 215 mmol, 68%) as a brown oil.

3. Fluorescence Studies

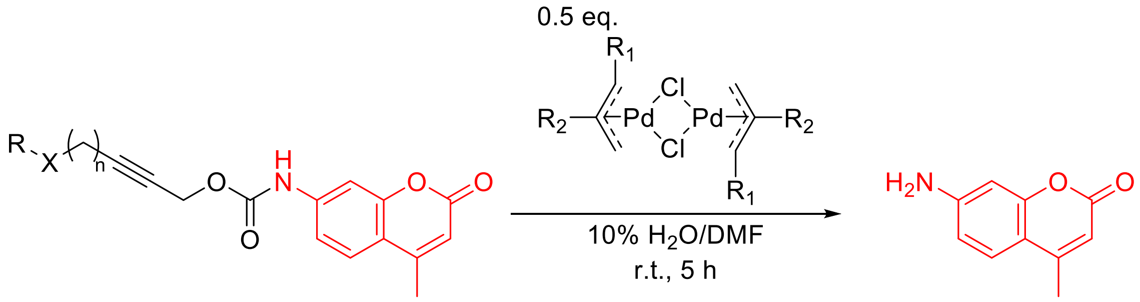

1: n = 0, X = H
6: n = 1, X = S, R = Me
7: n = 1, X = S, R = Trt
17: n = 1, X = S, R = ^{t}Bu
18: n = 1, X = S, R = BocCysOMe

Allyl: R_1, R_2 = H
1-Me allyl: R_1 = Me, R_2 = H
2-Me allyl: R_1 = H, R_2 = Me
Cinnamyl: R_1 = Ph, R_2 = H

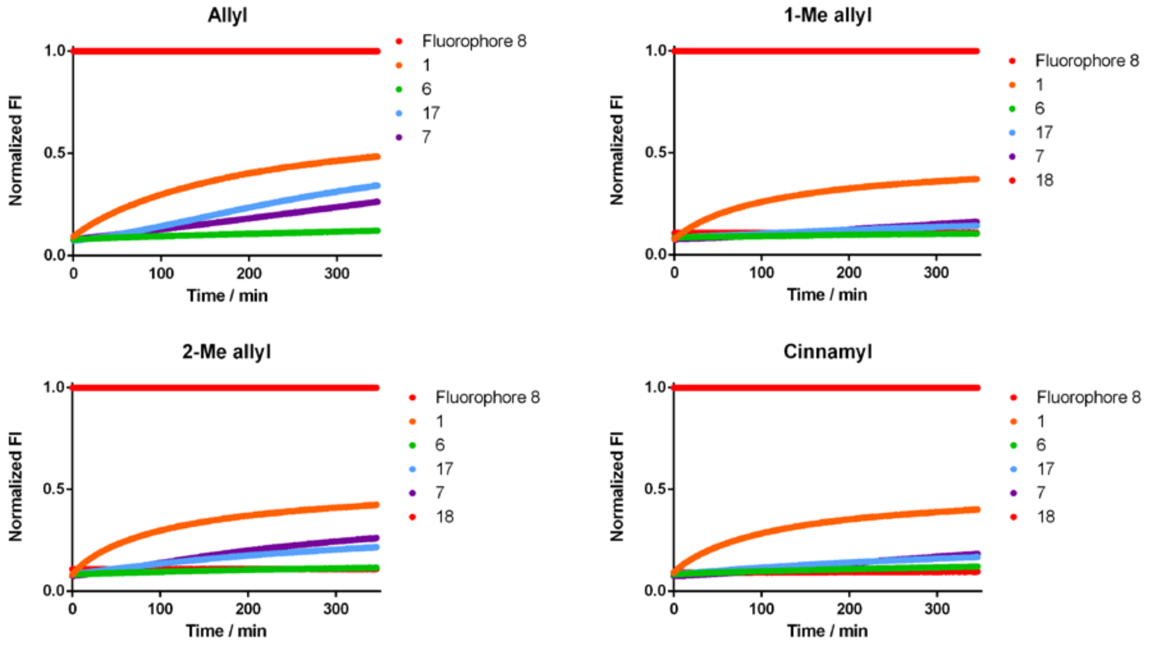

Figure S1: Decaging of propargyl carbamate derivative protected fluorophores with a series of allylpalladium chloride dimer derivatives.

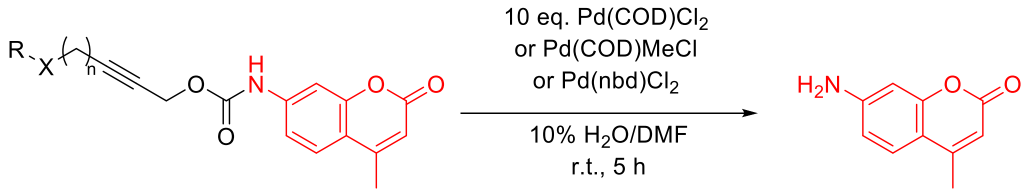

1: n = 0, X = H
7: n = 1, X = S, R = Trt
18: n = 1, X = S, R = BocCysOMe
19: n = 1, X = S, R = AcCysOMe
20: n = 1, X = S, R = CysOMe

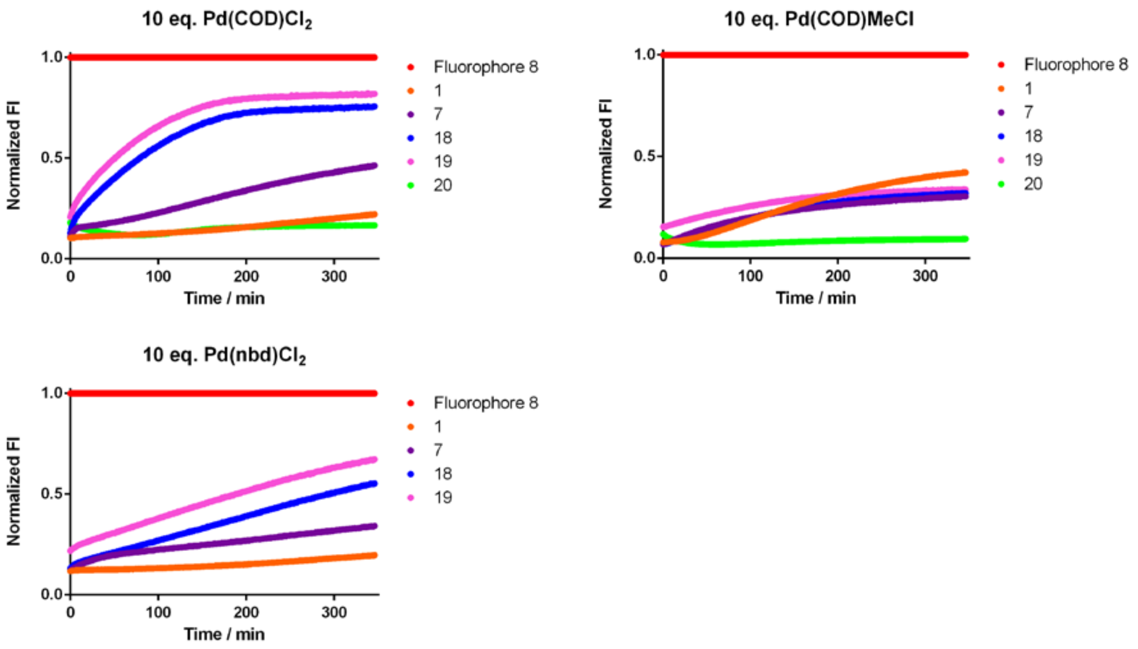

Figure S2: Decaging of propargyl carbamate derivative protected fluorophores with Pd(COD)Cl_2, Pd(COD)MeCl and Pd(nbd)Cl_2.

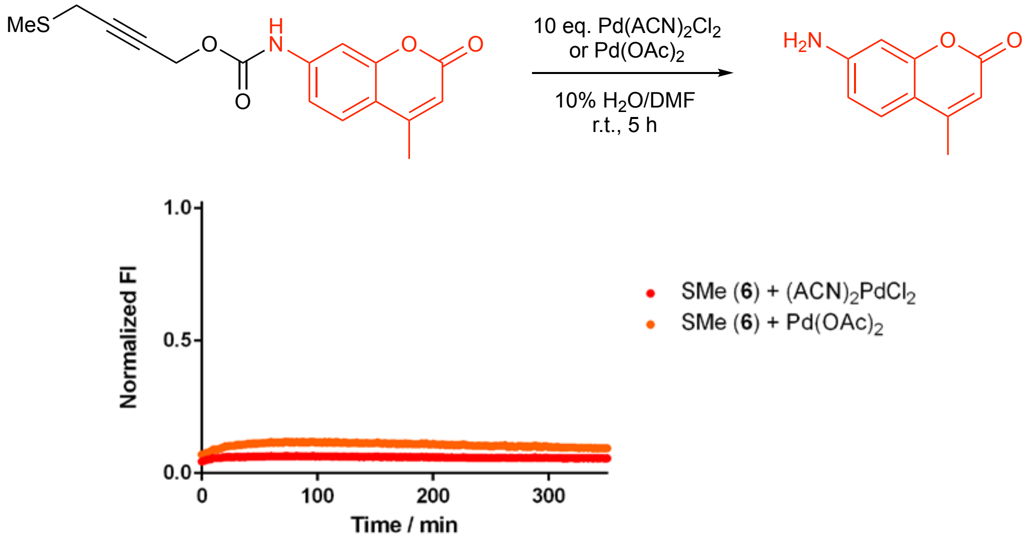

Figure S3: Decaging of propargyl carbamate derivative protected fluorophore (**6**) with 10 equivalents of Pd$(OAc)_2$ and Pd$(ACN)_2Cl_2$.

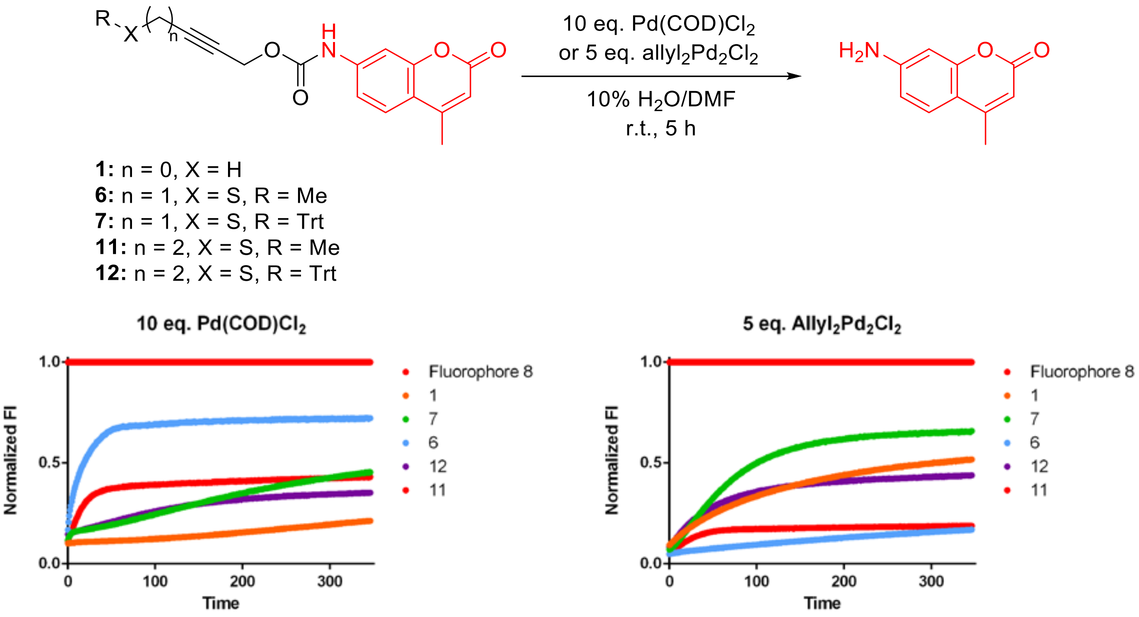

Figure S4: Determination of ideal spacer length between thioether and alkyne on a series of propargyl carbamate derivative protected fluorophores.

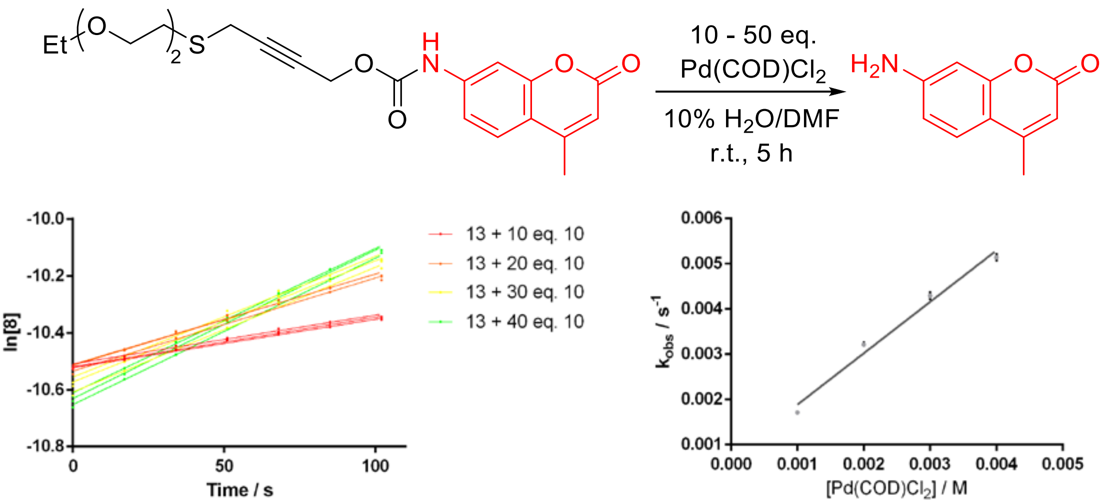

Figure S5: Decaging of PEGylated propargyl carbamate protected fluorophore **13** with Pd(COD)Cl_2 (**10**) at various concentrations to determine pseudo-first order rate constants and second order rate constant.

4. Antibody Modification

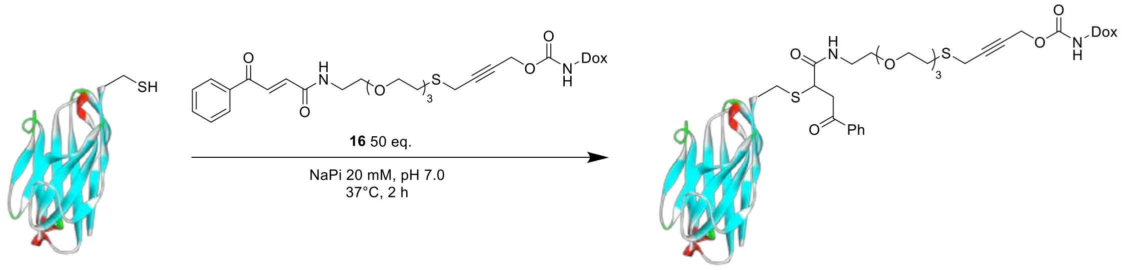

A 26.5 µL aliquot of a stock solution of anti-HER2 2Rb17c (76 µM, 2.0 nmol) was added to an eppendorf containing 61 µL NaPi 20 mM at pH 7.0. TCEP (100 nmol, 50 eq., 2.5 µL of a 40 mM solution) was added and the reaction was preincubated for 15 min at 37 °C. Afterwards a solution of acrylamide **16** (10 µL, 10 mM, 50 eq.) in DMF was added. The resulting mixture was vortexed for 15 seconds and the reaction was stirred for 2 h at 37 °C. A 10 µL aliquot was analyzed by LC-MS and complete conversion to the expected product was observed (calc. mass: 15864, obs. Mass: 15864). The reaction solution was purified by Vivaspin column.

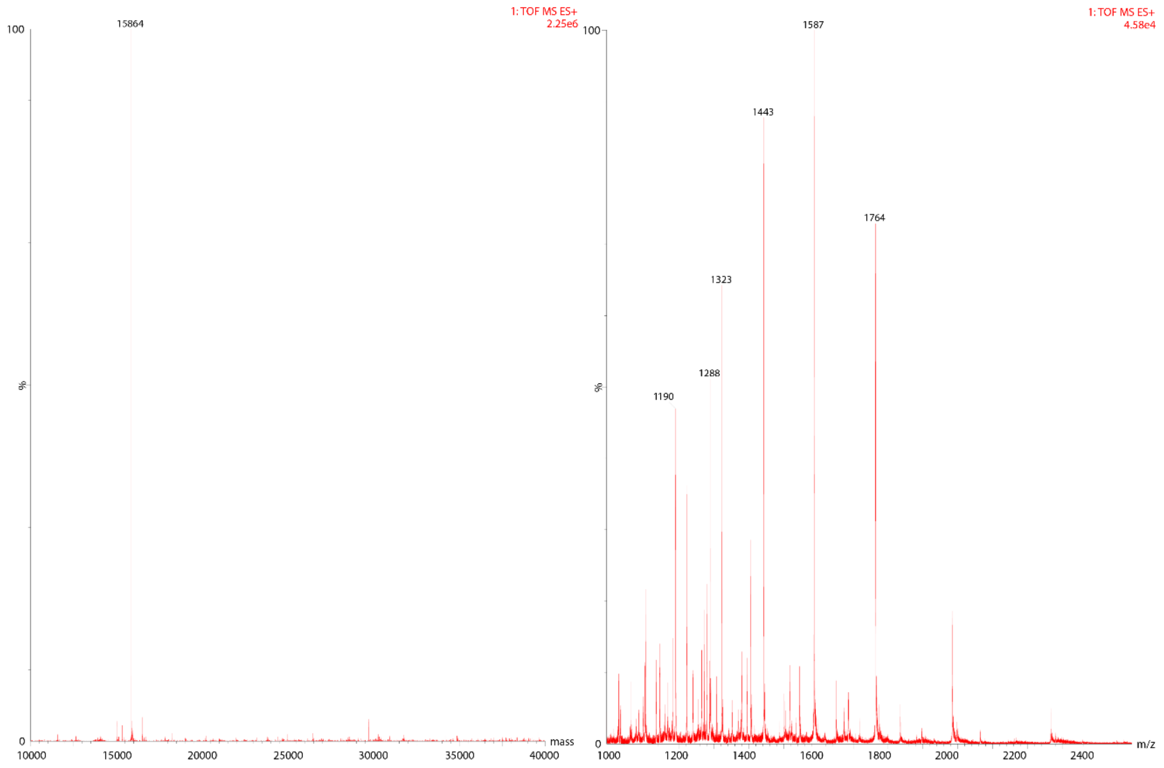

Figure S6: Deconvoluted spectrum and ion series of 2Rb17c-**16.**

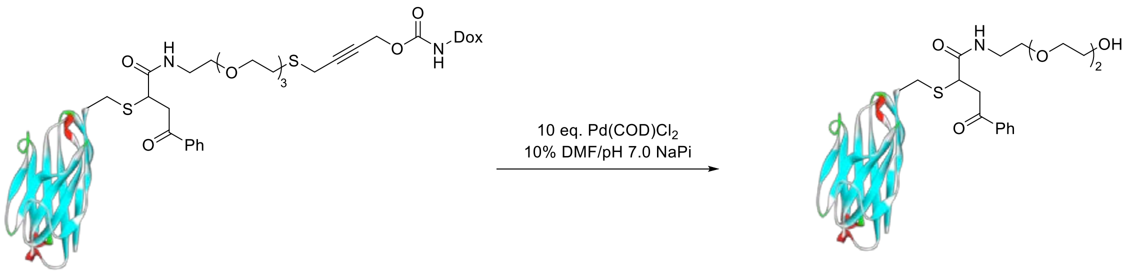

To 10 µL of a 20 µM solution of 2Rb17c-**16** in pH 7.0 20 mM NaPi was added pH 7.0 20 mM NaPi (8 uL) followed by 2 µL of a 1 mM solution of Pd(COD)Cl_2 in DMF. The reaction was vortexed for 10 s then stirred at 37 °C for 2 h. After this time, 2 uL of a 3 mM solution of 3-mercaptopropionic acid was added in DMF and the reaction analyzed by LCMS.

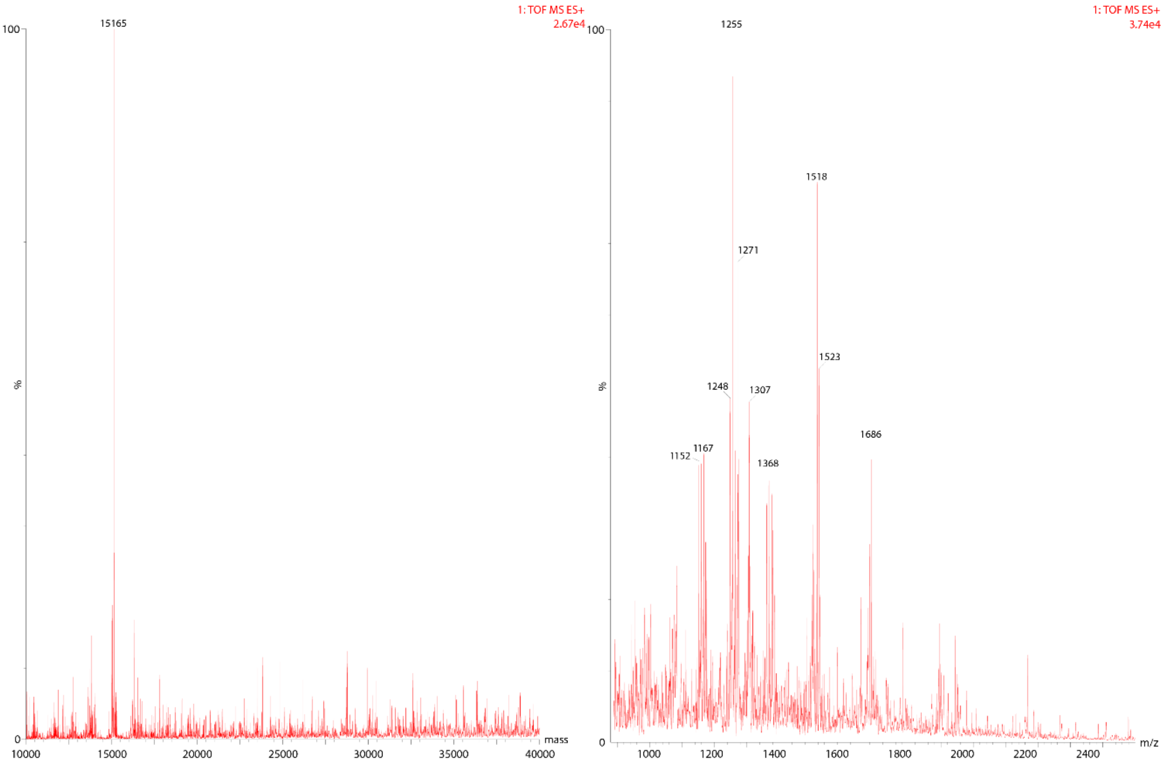

Figure S7: Deconvoluted spectrum and ion series of decaged 2Rb17c-**16**.

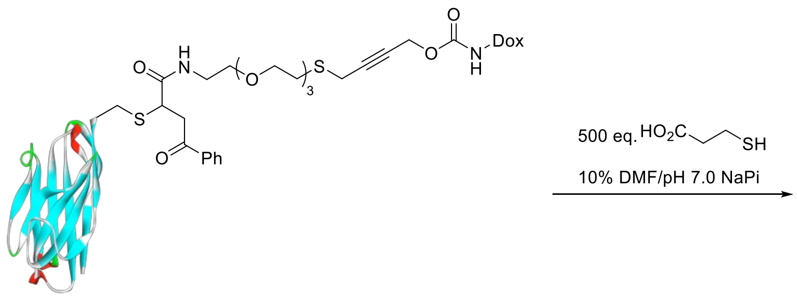

To 4 µL of a 20 µM solution of ADC in pH 7.0 20 mM NaPi was added pH 7.0 20 mM NaPi (5 uL) followed by 1 µL of a 50 mM solution of 3-mercaptopropionic acid was added in DMF and the reaction analyzed by LCMS.

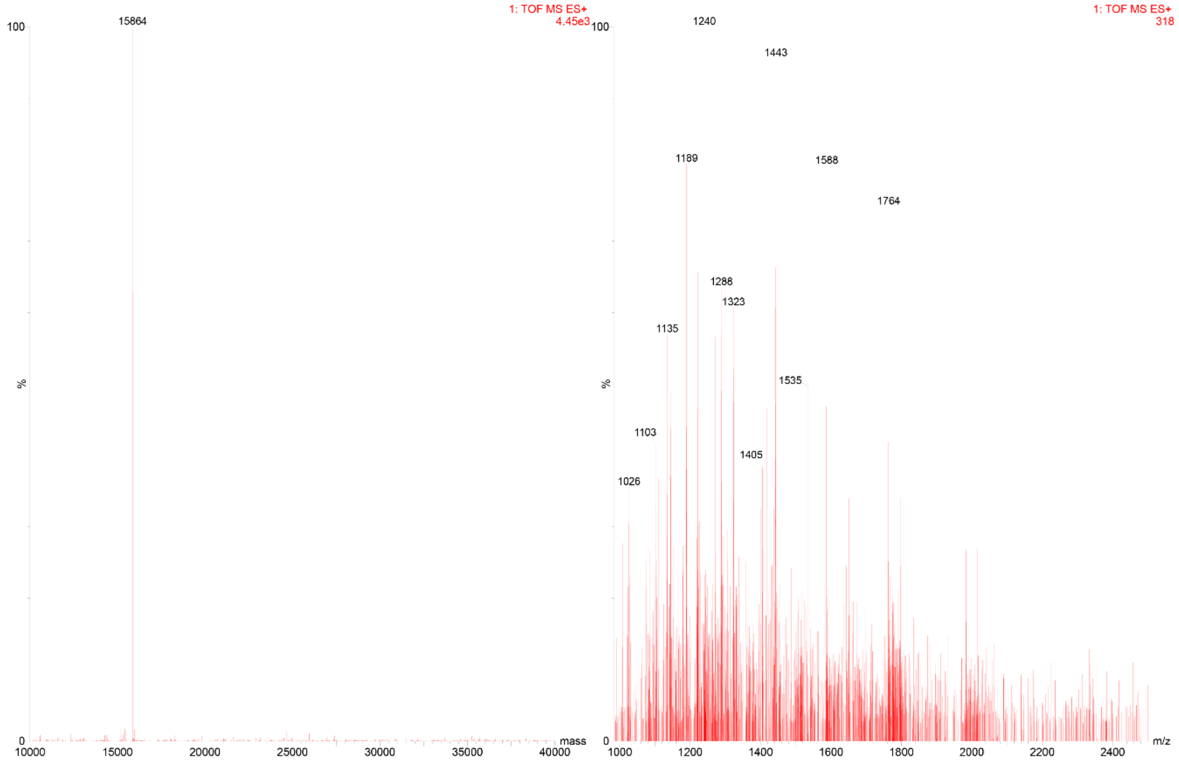

Figure S8: Deconvoluted spectrum and ion series of 2Rb17c-**16** after stability testing to thiols.

4.1 Proposed Mechanism of Decaging

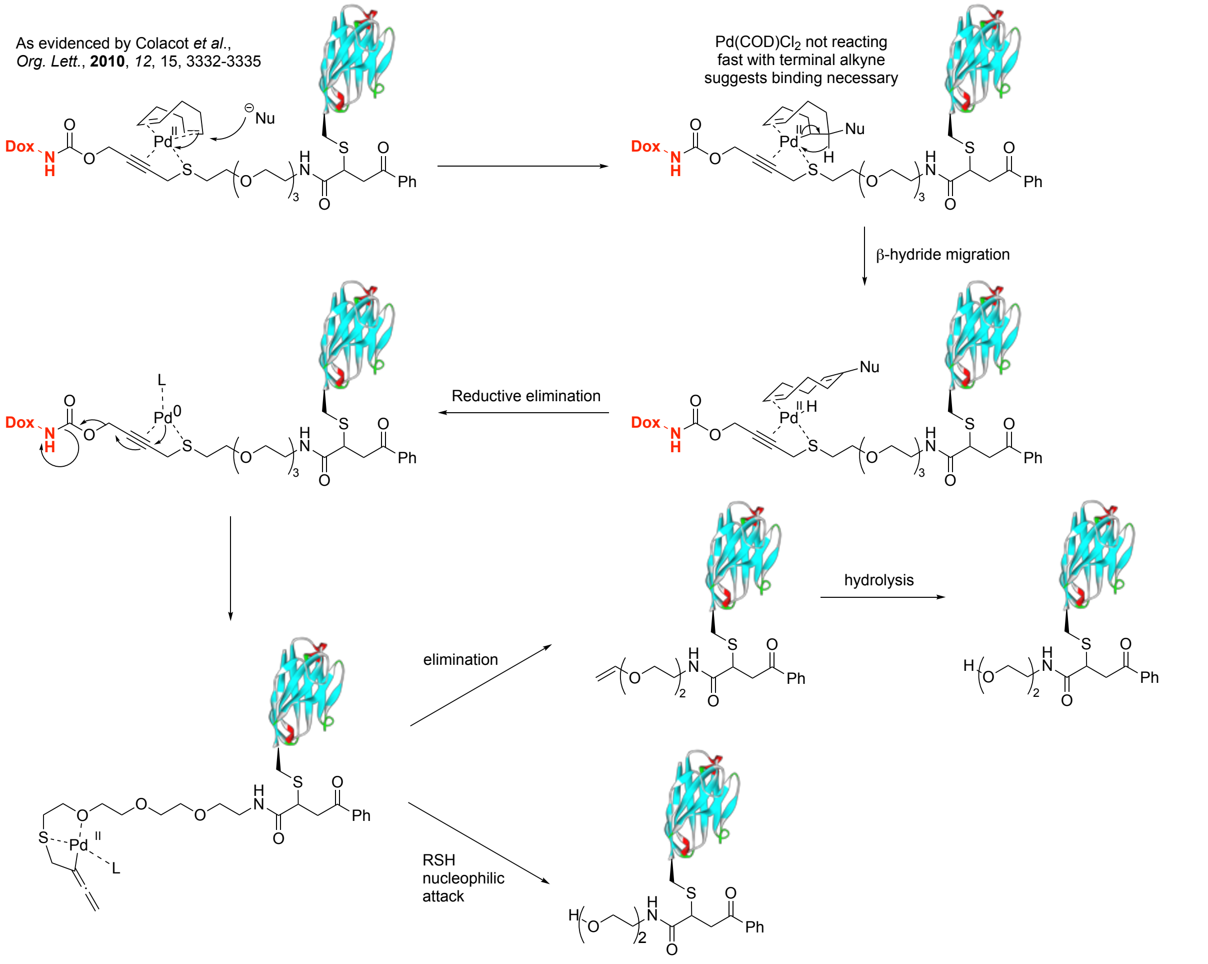

Figure S9: Complexes of Pd(II) with COD are known to reduce to Pd(0) in the presence of nucleophiles by reductive elimination.[13,14] Formation of the final product could be by elimination and subsequent hydrolysis of the vinyl ether, or directly by nucleophilic substitution with water or the palladium scavenger.

5. Cell Studies

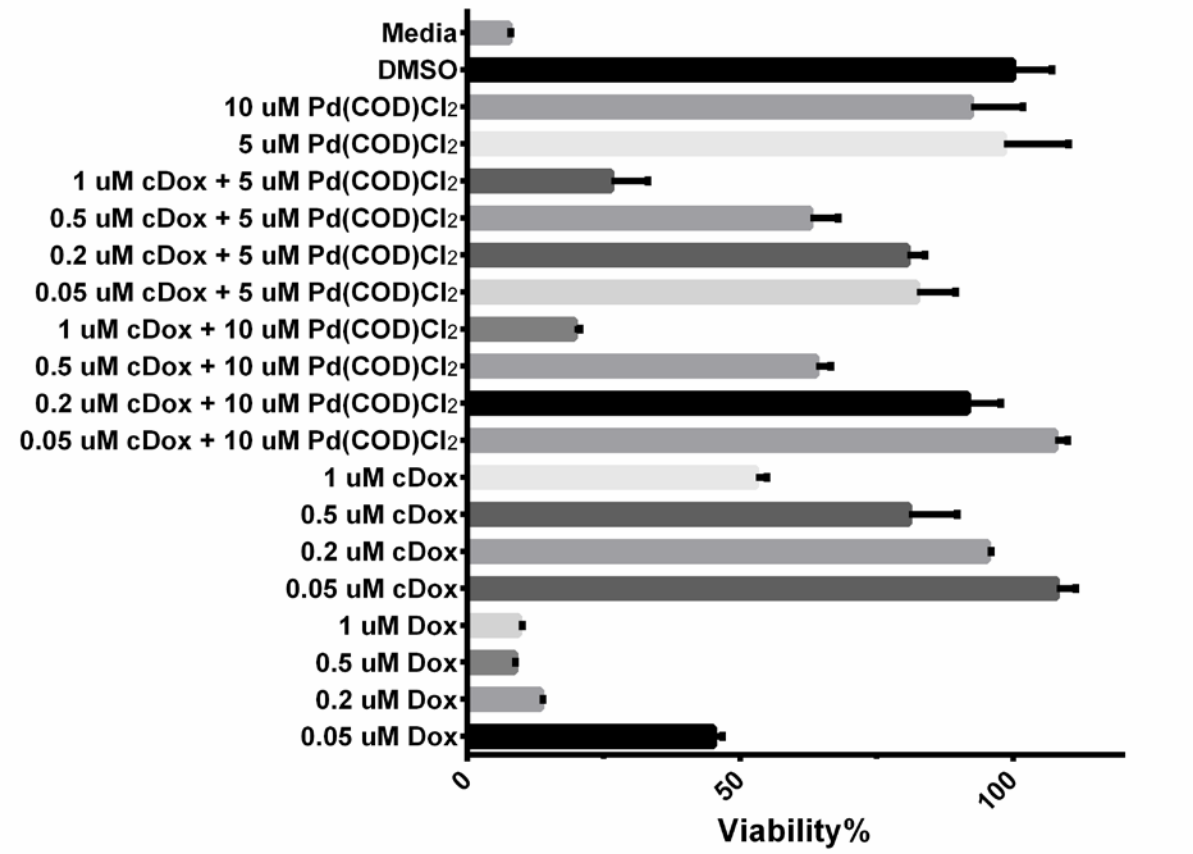

Figure S10: Toxicity data in HEK cells for Pd(COD)Cl_2 (concentrations given once every 24 h), Doxorubicin HCl (**Dox, 14**), caged Doxorubicin (**cDox, 15**) against 1% DMSO. **Dox** (**14**) and **cDox** (**15**) were dissolved in DMSO and added to cells in 1 µL. Pd(COD)Cl_2 was dissolved in 50% DMSO/H_2O and added to cells in 1 µL. All cell viabilities are normalized with respect to DMSO control.

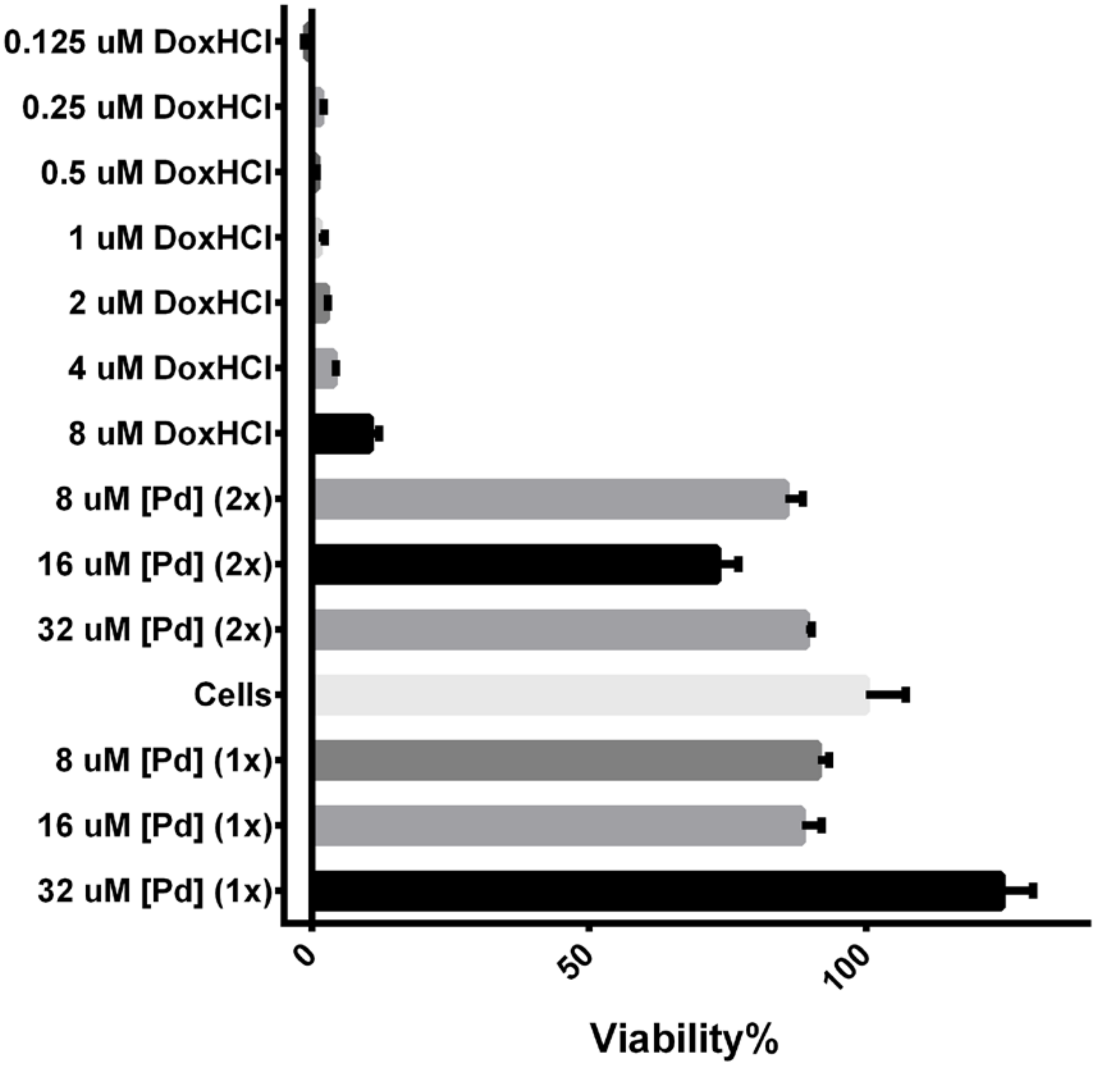

Figure S11: Toxicity data in MCF7 cells for Pd(COD)Cl_2 ([Pd] concentrations given once every 24 h or twice daily at 8 and 16 h intervals), Doxorubicin HCl (Dox) against 0.5% DMSO. Doxorubicin HCl was dissolved in DMSO and added to cells in 1 µL. Pd(COD)Cl_2 was dissolved in 1% DMSO/H_2O and added to cells in 1 µL. Cells were seeded at 3000 cells/well in 200 µL. All cell viabilities were normalized with respect to DMSO control.

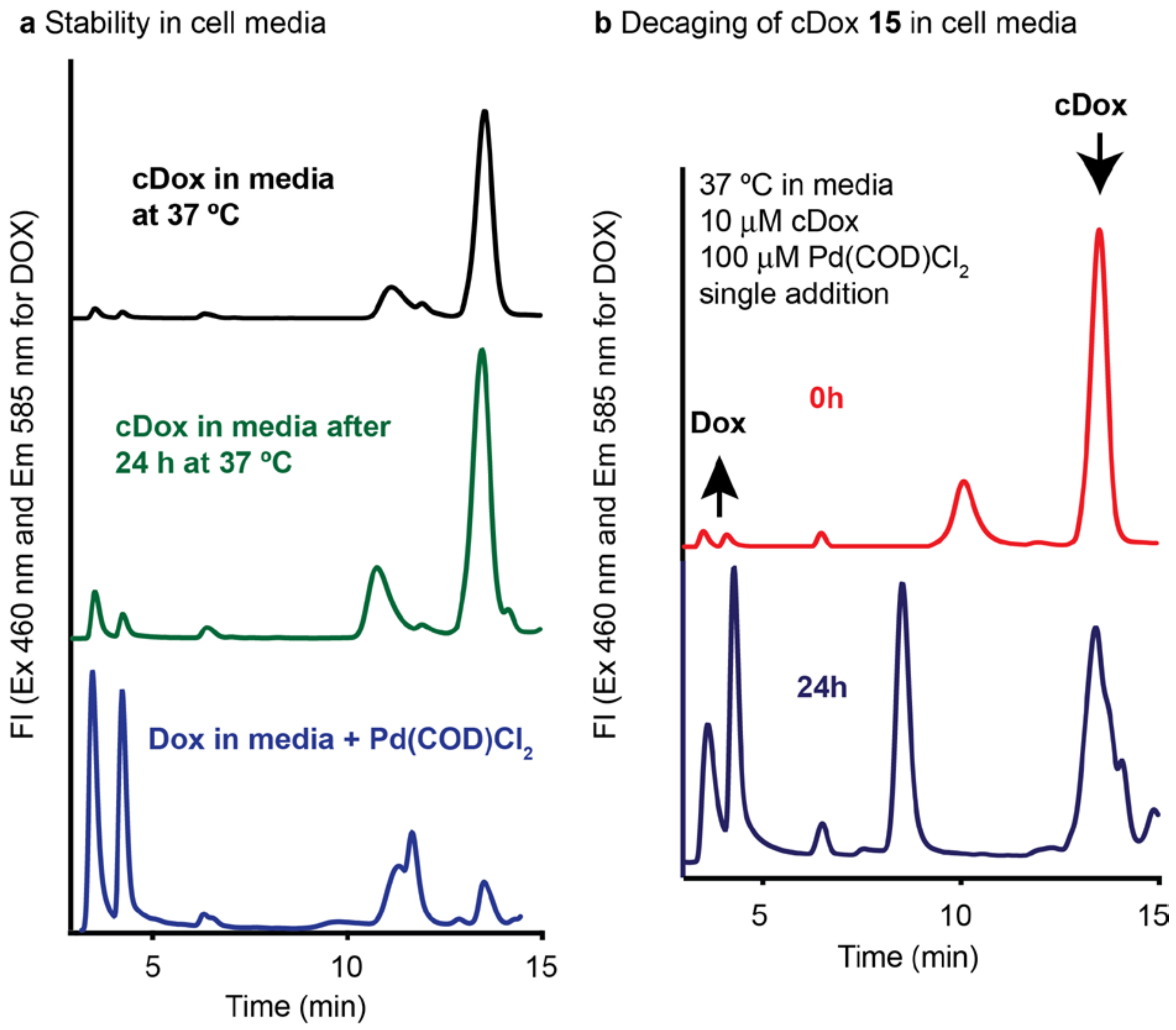

Figure S12: Stability and decaging of cDox **15** in cell media analyzed by HPLC. **a.** HPLC traces of cDox **15** in cellular media at 37 ºC at 0 and 24 h, and Dox **14** in the presence of the palladium catalyst **10**. **b.** Decaging of cDox **15** in the presence of **10** at 0 and 24 h.

<u>HPLC conditions:</u>

Column: Phenomenex luna 5 µm NH2 100A
Dimensions: 250 x 4.6 mm
Mobile Phase: A - H2O 0.1% Formic Acid, B - ACN:H_2O (71:29) 0.1% FA
Flow Rate: 0.5 mL/min
Gradient: 0 to 65% of B in 9 min then to 100% of B in 3 min followed by 4 min at 100% of B.

6. NMR Spectra

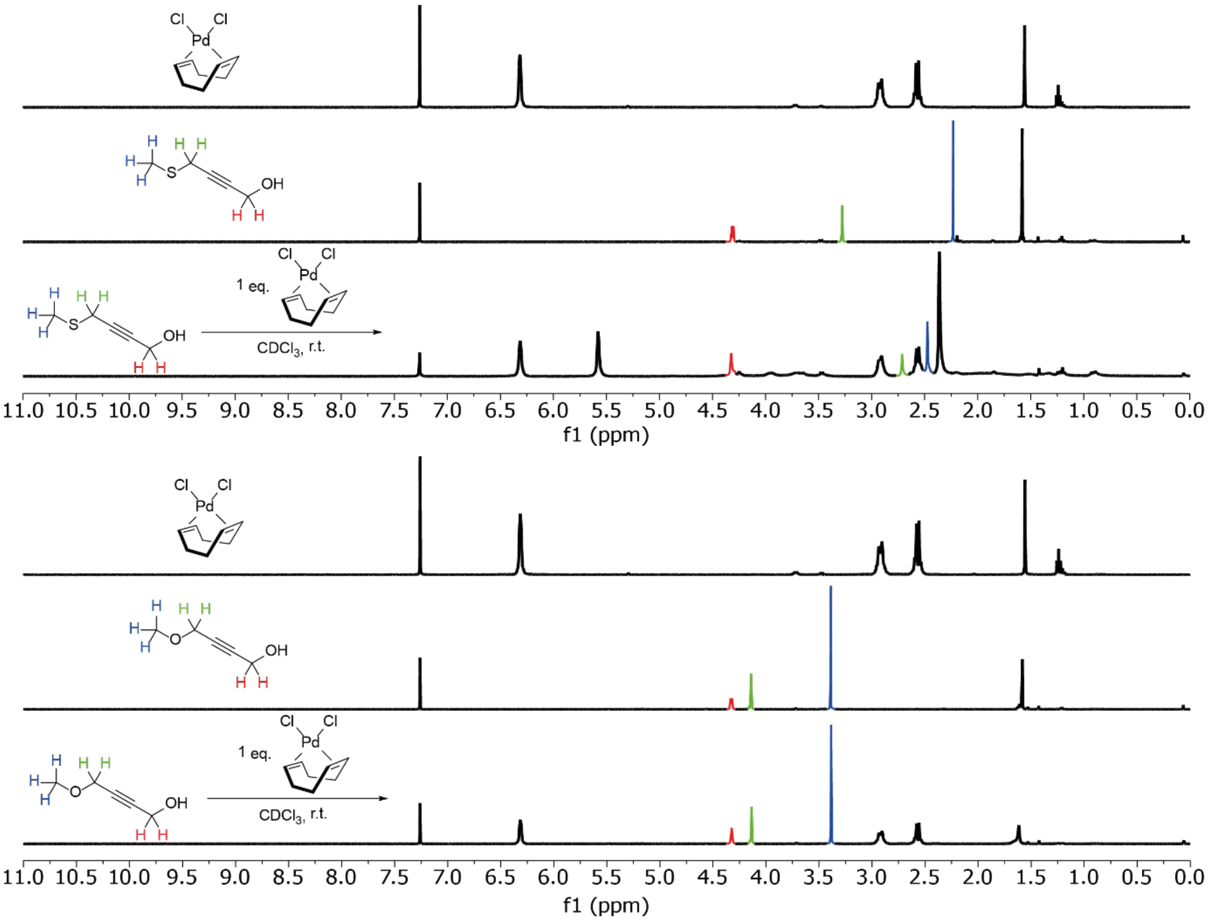

Figure S13: Binding of propargyl thioether and propargyl ethers to Pd(COD)Cl_2.

4-(dimethylamino)but-2-ynl (7-amino-4-methylcoumarin)carbamate (2)

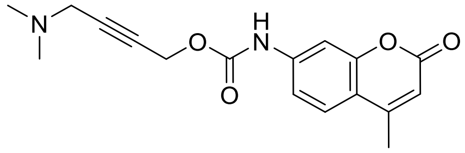

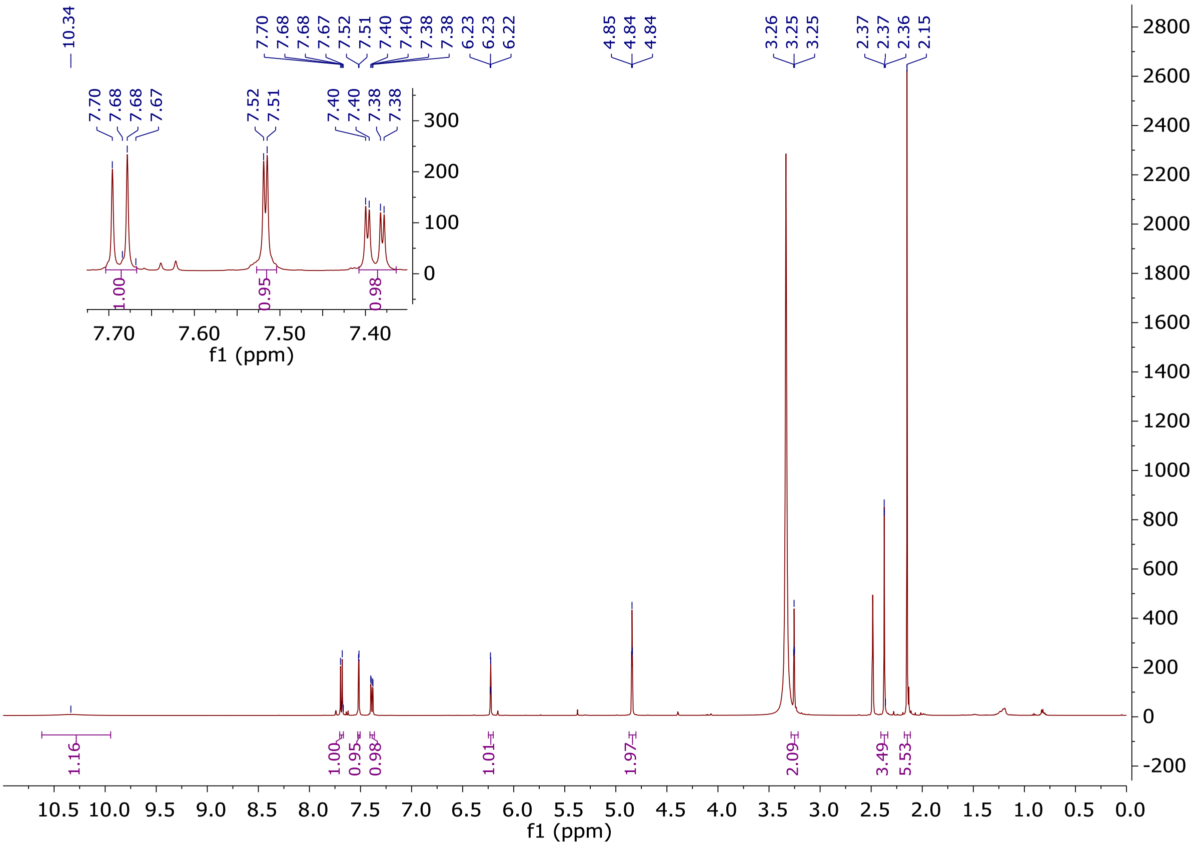

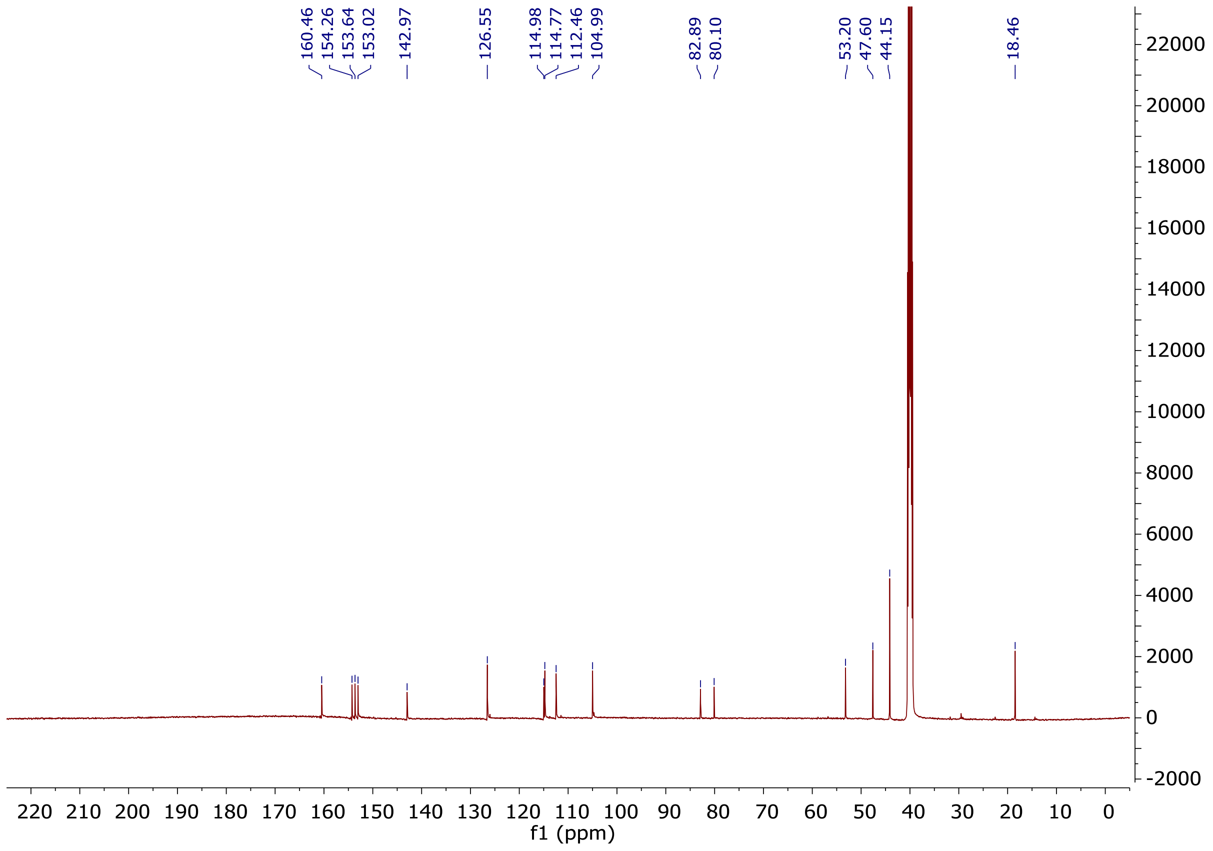
160.46
154.26
153.64
153.02
142.97
126.55
114.98
114.77
112.46
104.99
82.89
80.10
53.20
47.60
44.15
18.46
22000
20000
18000
16000
14000
12000
10000
8000
6000
4000
2000
0
-2000
220 210 200 190 180 170 160 150 140 130 120 110 100 90 80 70 60 50 40 30 20 10 0
f1 (ppm)

4-(trityloxy)but-2-ynl (7-amino-4-methylcoumarin)carbamate (3)

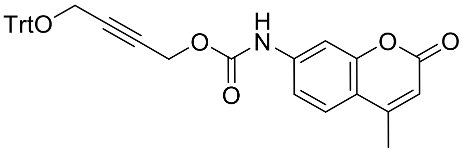

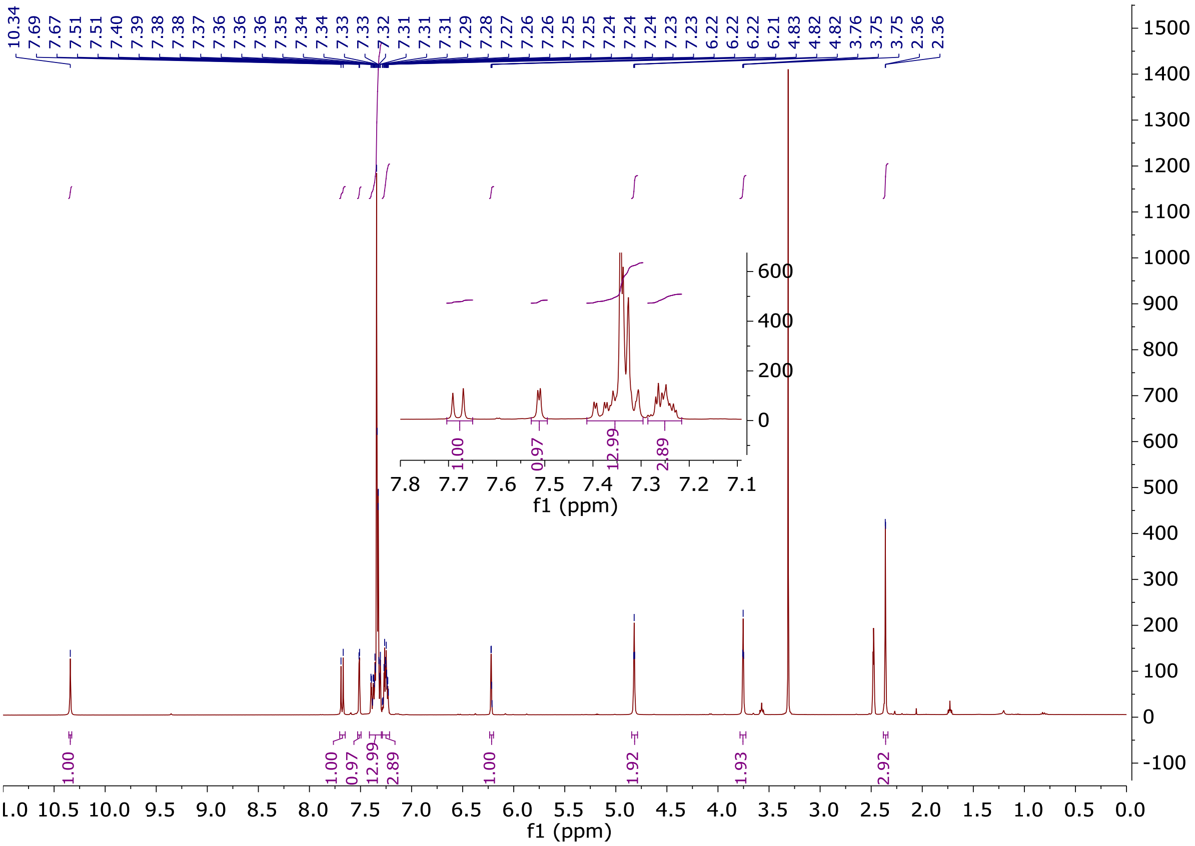

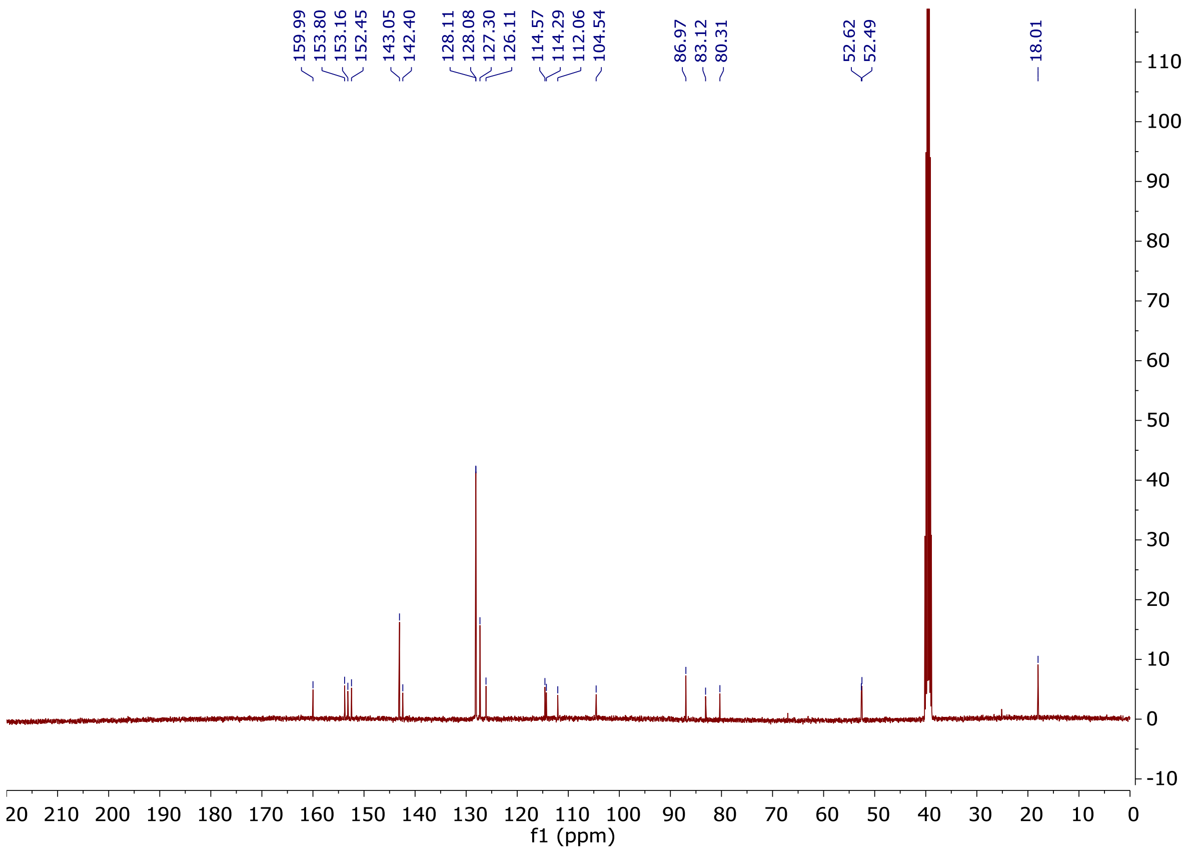
159.99
153.80
153.16
152.45
143.05
142.40
128.11
128.08
127.30
126.11
114.57
114.29
112.06
104.54
86.97
83.12
80.31
52.62
52.49
18.01
f1 (ppm)

4-methoxybut-2-ynl (7-amino-4-methylcoumarin)carbamate (4)

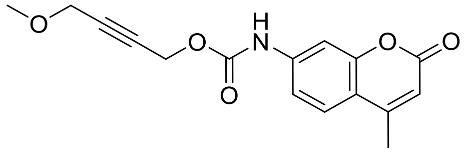

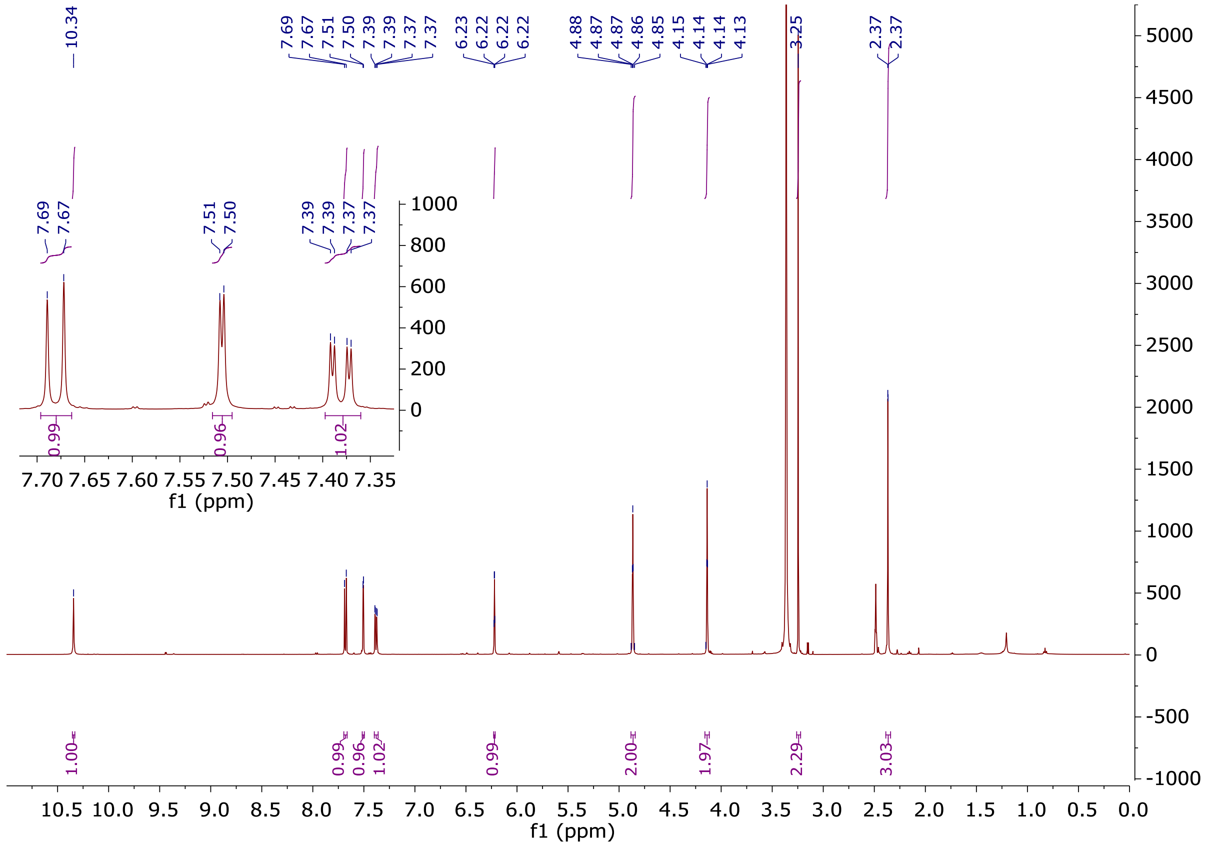

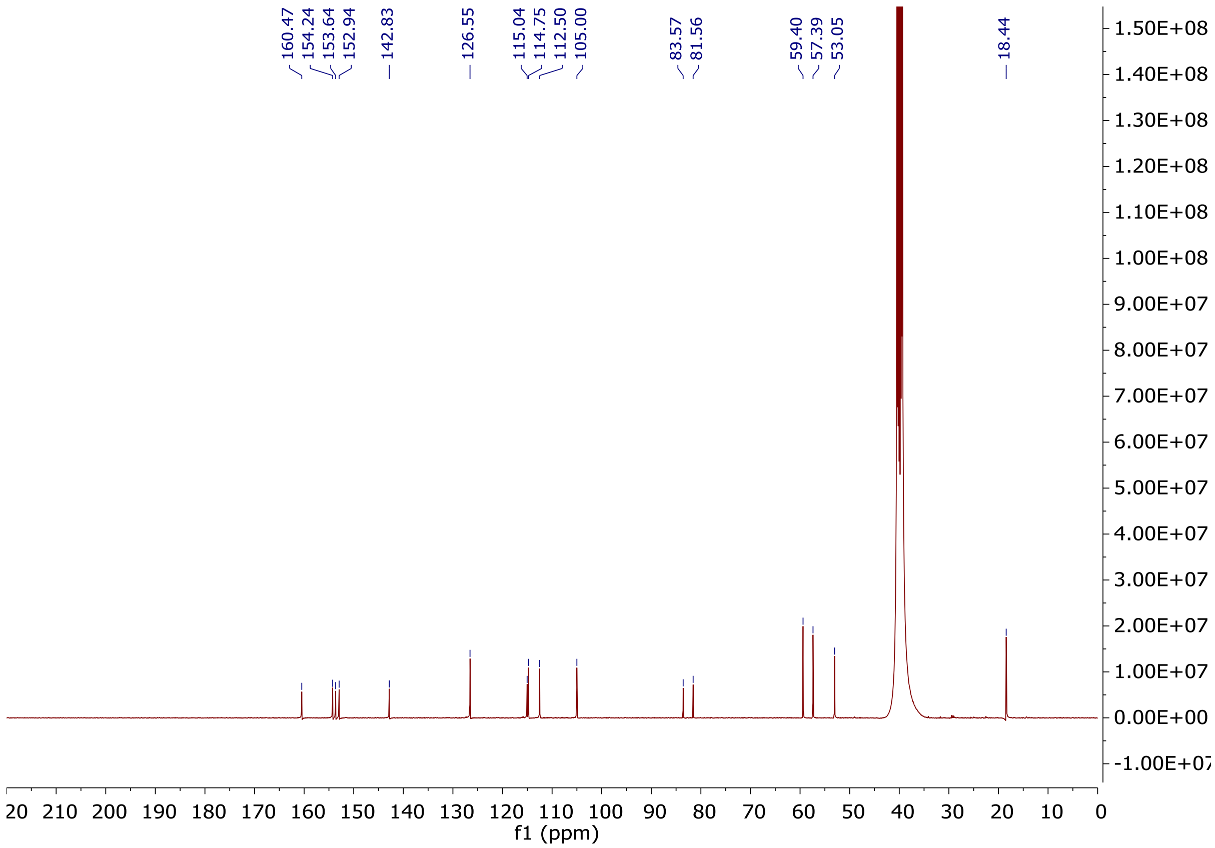
160.47
154.24
153.64
152.94
142.83
126.55
115.04
114.75
112.50
105.00
83.57
81.56
59.40
57.39
53.05
18.44
1.50E+08
1.40E+08
1.30E+08
1.20E+08
1.10E+08
1.00E+08
9.00E+07
8.00E+07
7.00E+07
6.00E+07
5.00E+07
4.00E+07
3.00E+07
2.00E+07
1.00E+07
0.00E+00
-1.00E+07
20 210 200 190 180 170 160 150 140 130 120 110 100 90 80 70 60 50 40 30 20 10 0
f1 (ppm)

Hex-2-yn-1-yl (7-amino-4-methylcoumarin)carbamate (5)

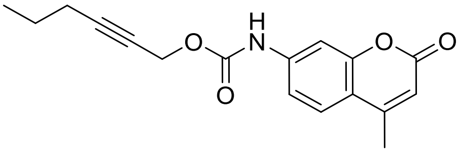

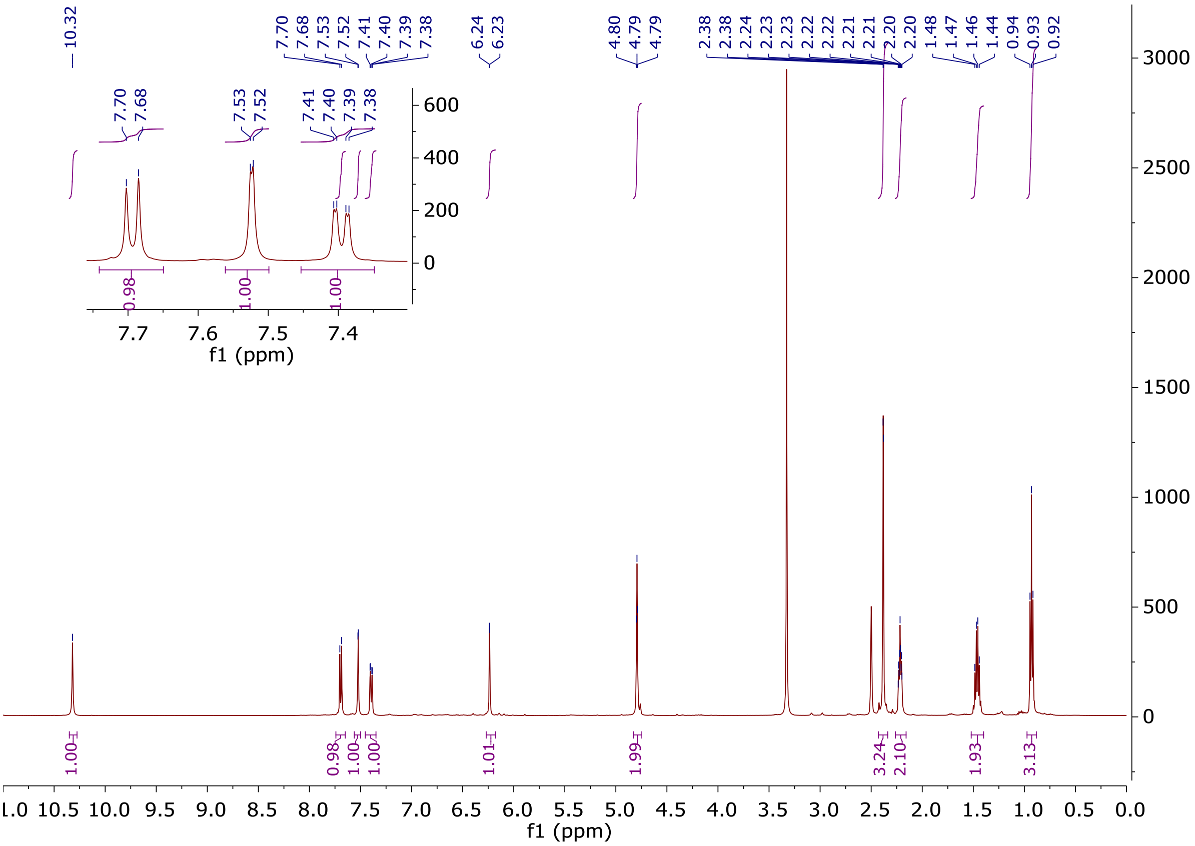

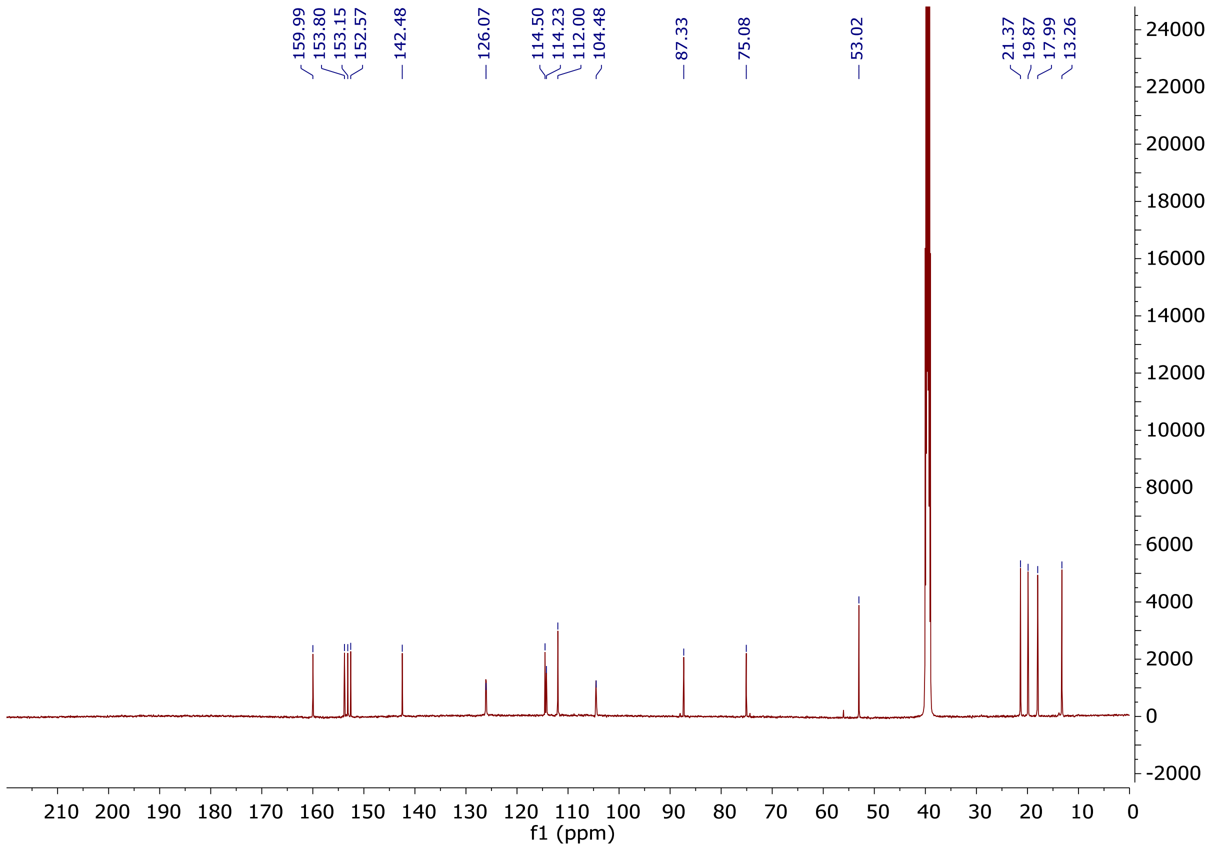
159.99
153.80
153.15
152.57
142.48
126.07
114.50
114.23
112.00
104.48
87.33
75.08
53.02
21.37
19.87
17.99
13.26
24000
22000
20000
18000
16000
14000
12000
10000
8000
6000
4000
2000
0
-2000
210 200 190 180 170 160 150 140 130 120 110 100 90 80 70 60 50 40 30 20 10 0
f1 (ppm)

4-(methylthio)but-2-yn-1-ol (6a)

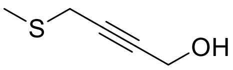

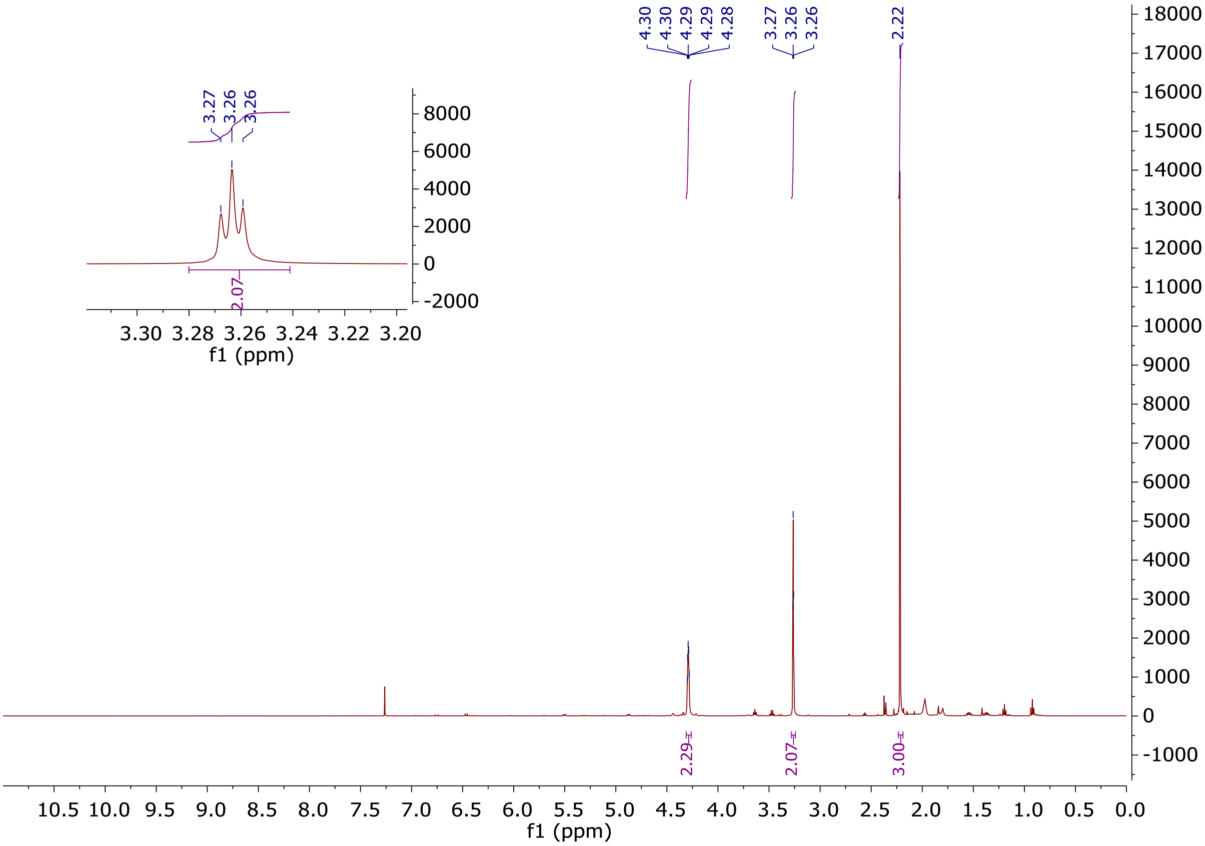

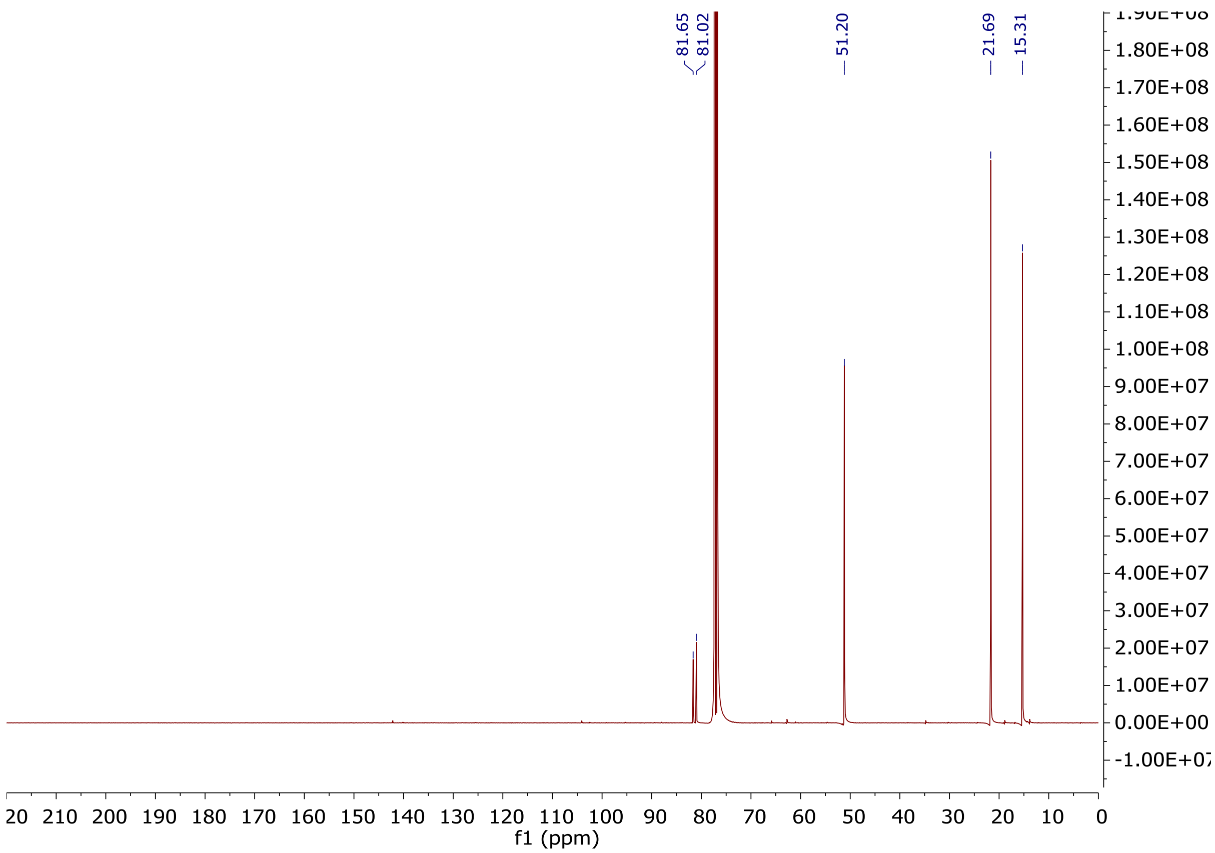

81.65
81.02
51.20
21.69
15.31
1.80E+08
1.70E+08
1.60E+08
1.50E+08
1.40E+08
1.30E+08
1.20E+08
1.10E+08
1.00E+08
9.00E+07
8.00E+07
7.00E+07
6.00E+07
5.00E+07
4.00E+07
3.00E+07
2.00E+07
1.00E+07
0.00E+00
20 210 200 190 180 170 160 150 140 130 120 110 100 90 80 70 60 50 40 30 20 10 0
f1 (ppm)

4-(methylthio)but-2-ynl (7-amino-4-methylcoumarin)carbamate (6)

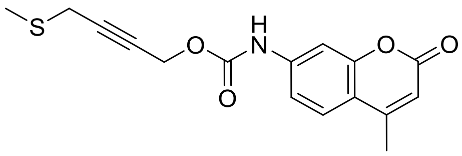

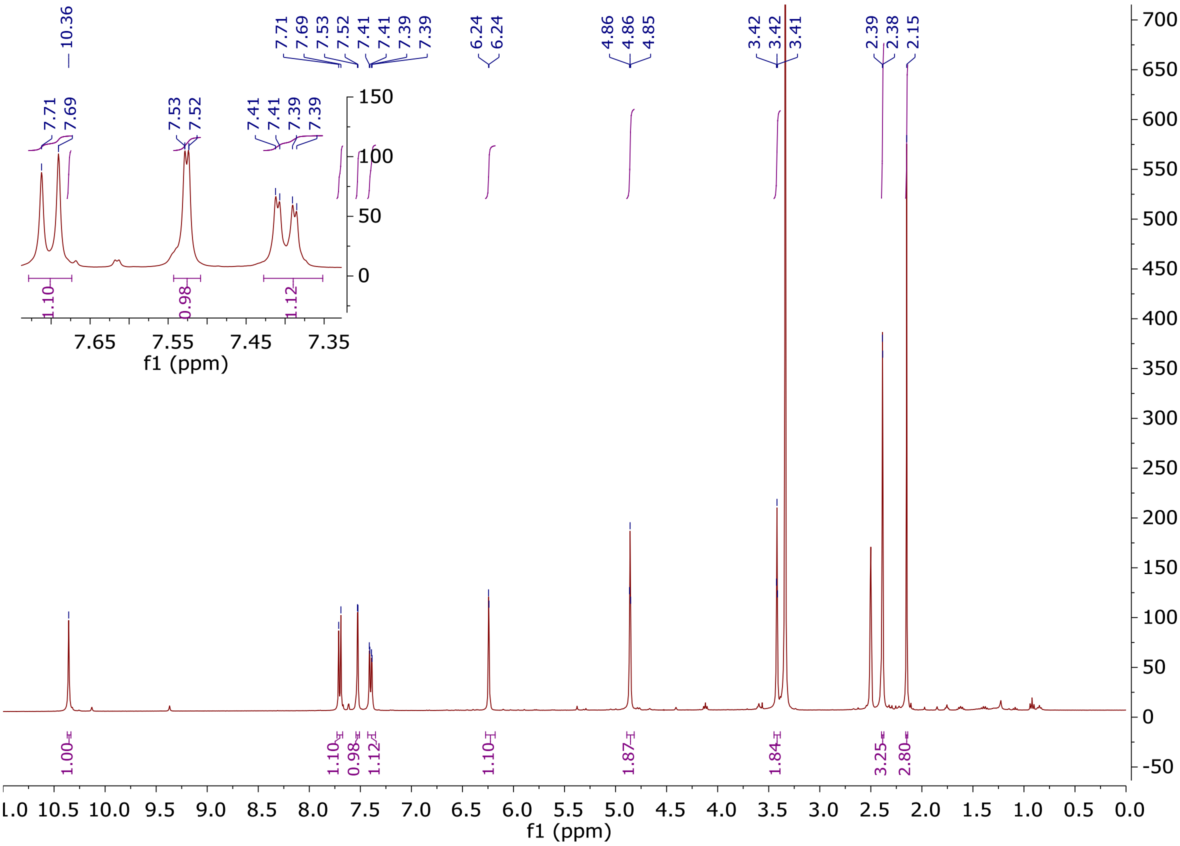

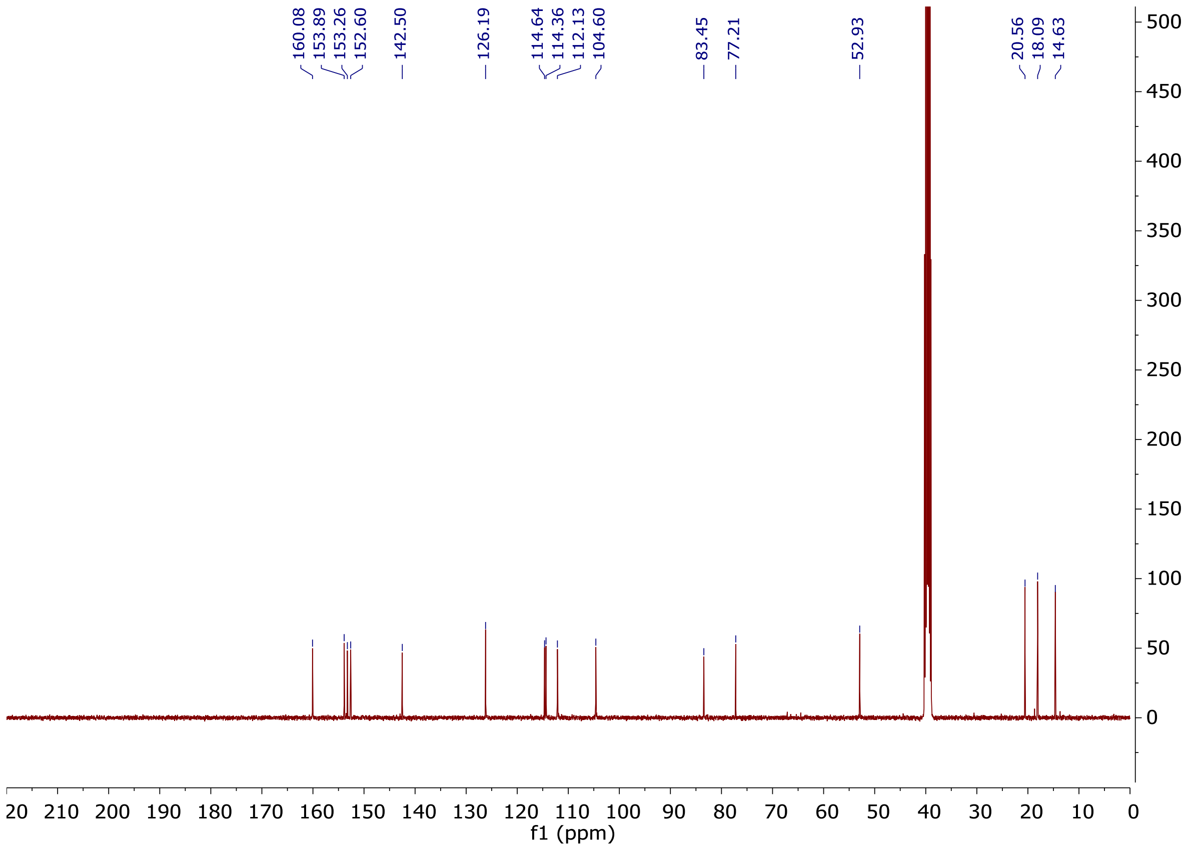
160.08
153.89
153.26
152.60
142.50
126.19
114.64
114.36
112.13
104.60
83.45
77.21
52.93
20.56
18.09
14.63
500
450
400
350
300
250
200
150
100
50
0
20 210 200 190 180 170 160 150 140 130 120 110 100 90 80 70 60 50 40 30 20 10 0
f1 (ppm)

Propargyl trityl thioether (7a)

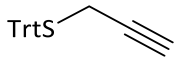

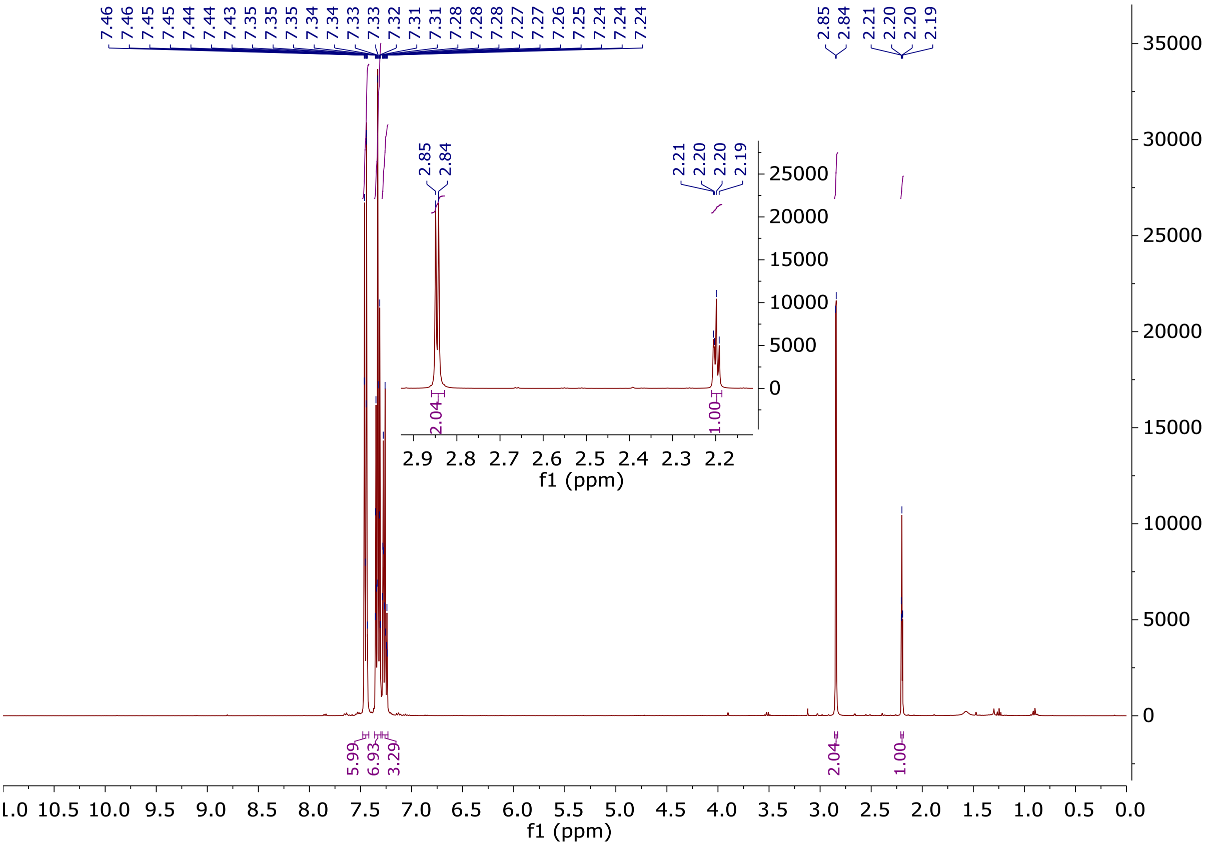

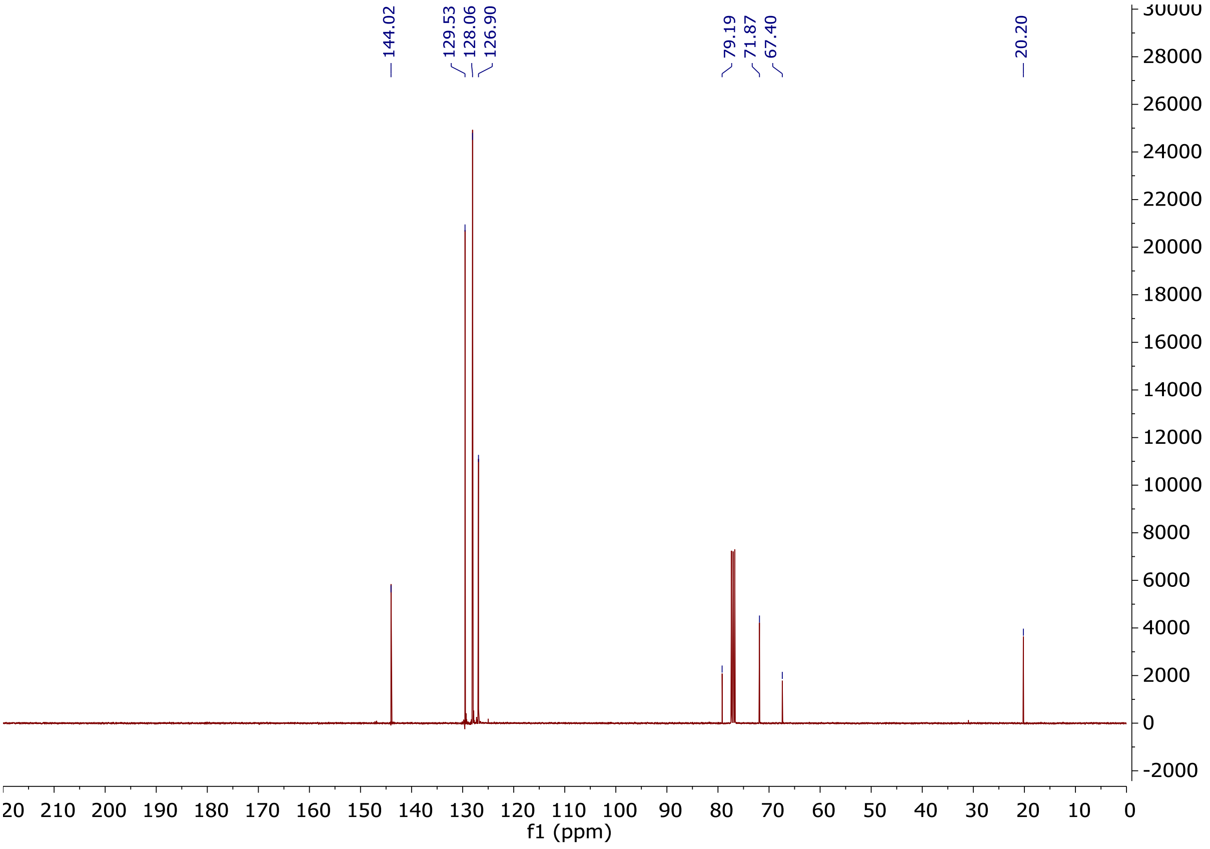

4-(tritylthio)but-2-yn-1-ol (7b)

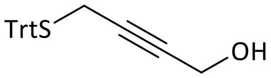

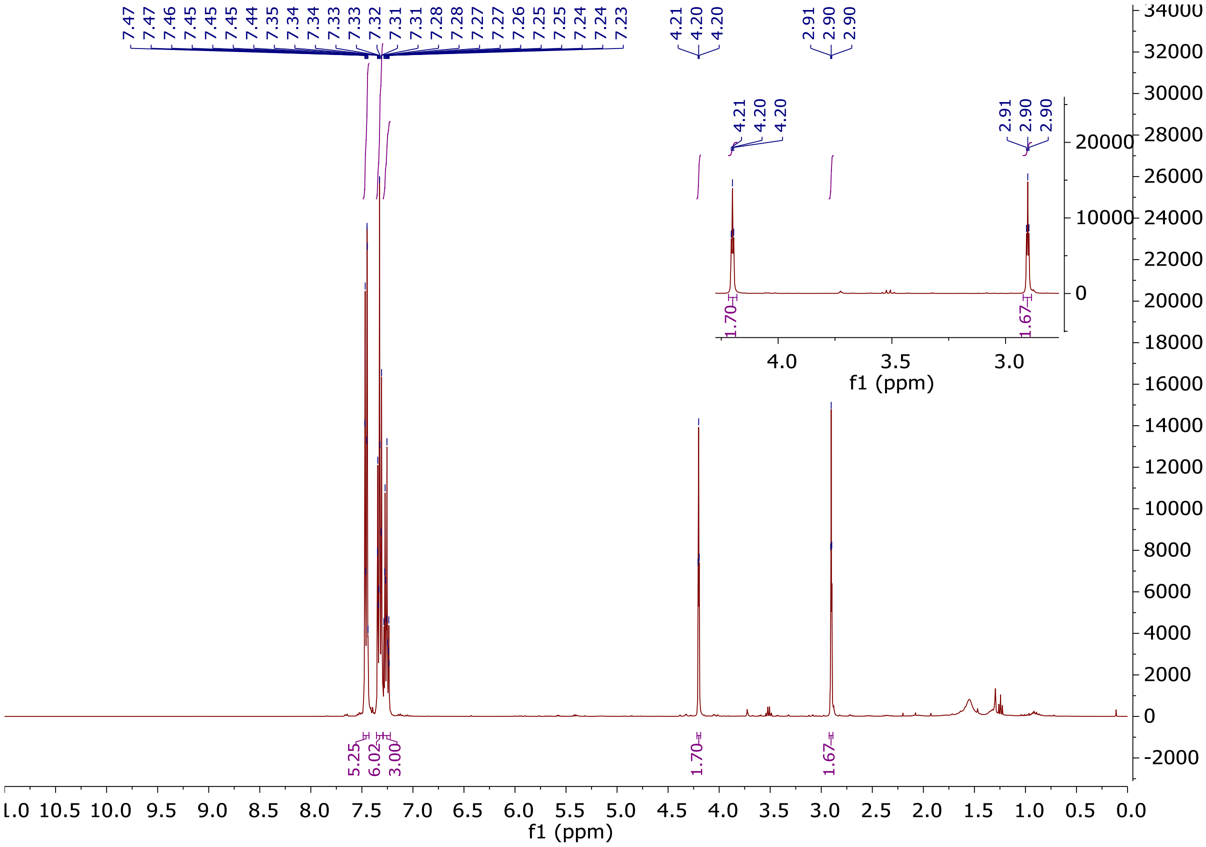

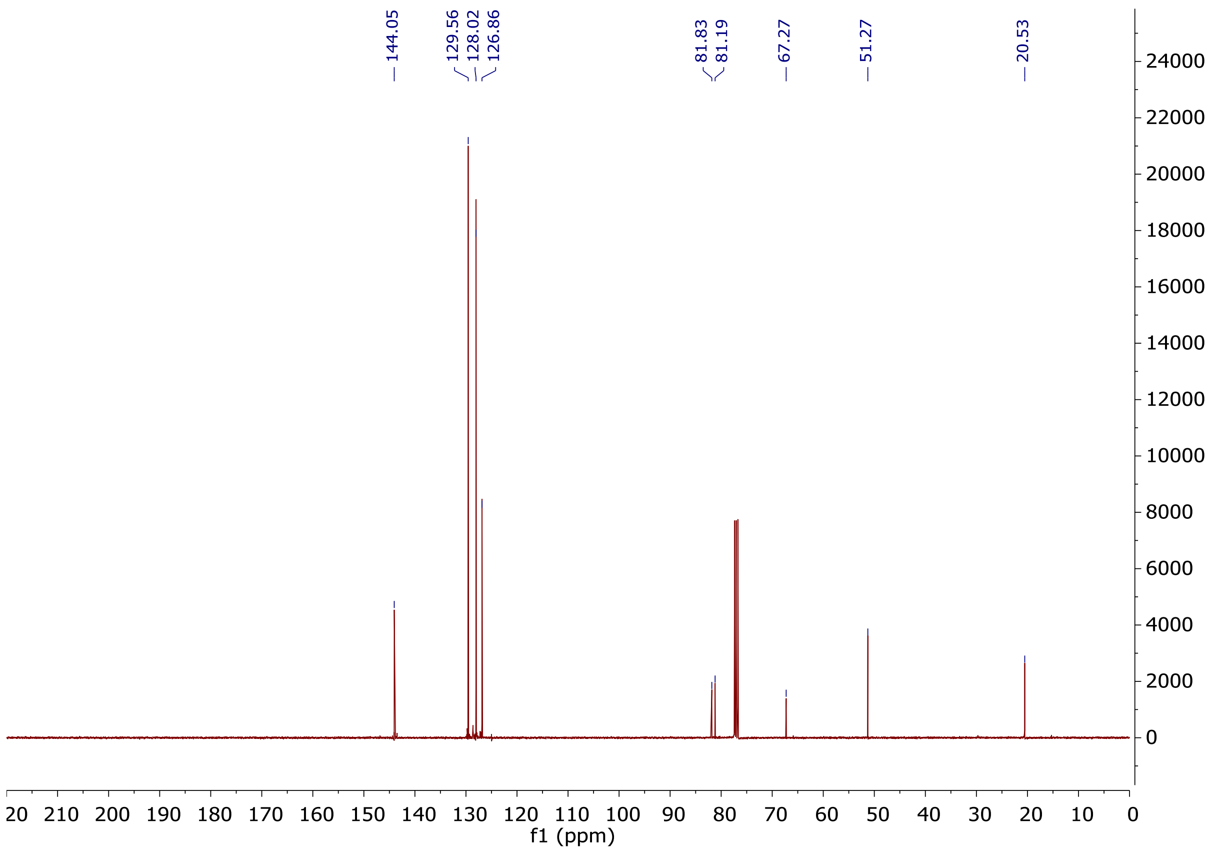

144.05
129.56
128.02
126.86
81.83
81.19
67.27
51.27
20.53
24000
22000
20000
18000
16000
14000
12000
10000
8000
6000
4000
2000
0
20 210 200 190 180 170 160 150 140 130 120 110 100 90 80 70 60 50 40 30 20 10 0
f1 (ppm)

4-(tritylthio)but-2-ynl (7-amino-4-methylcoumarin)carbamate (7)

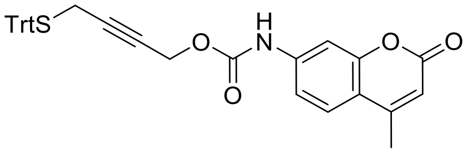

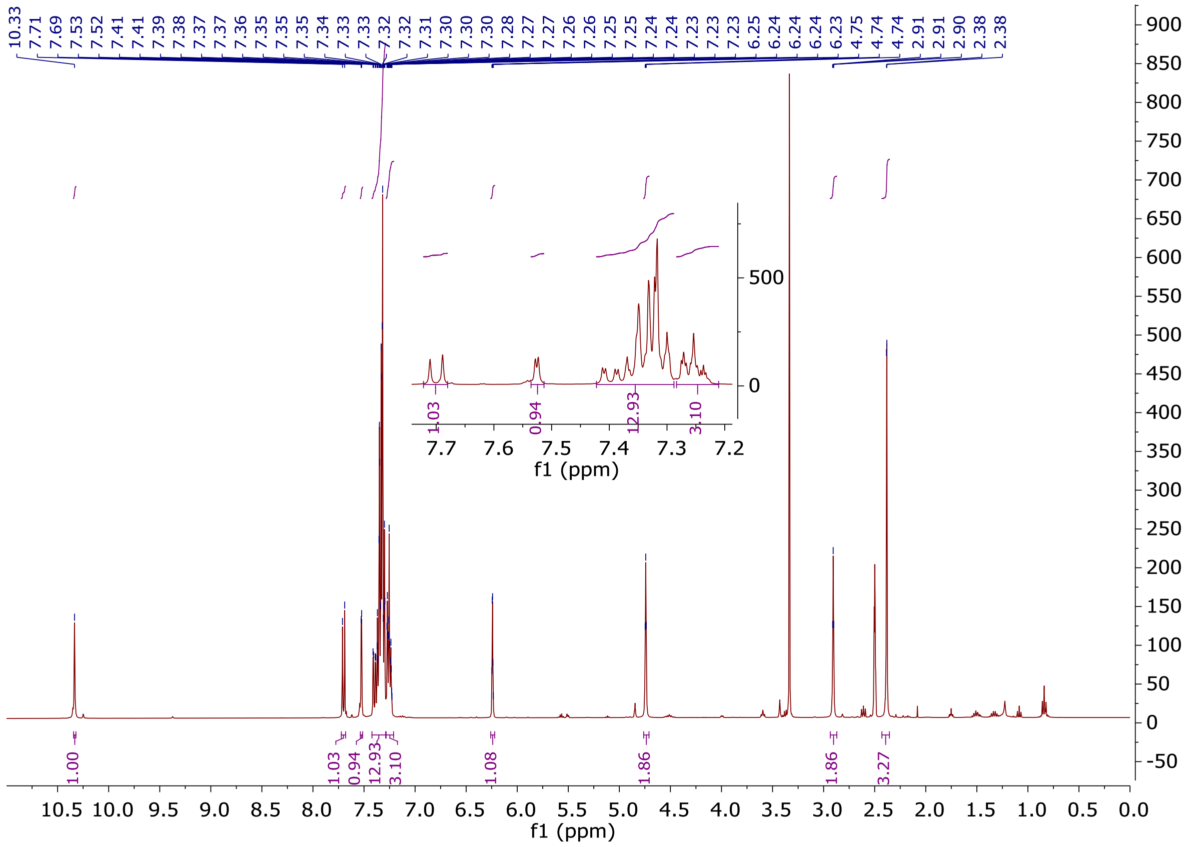

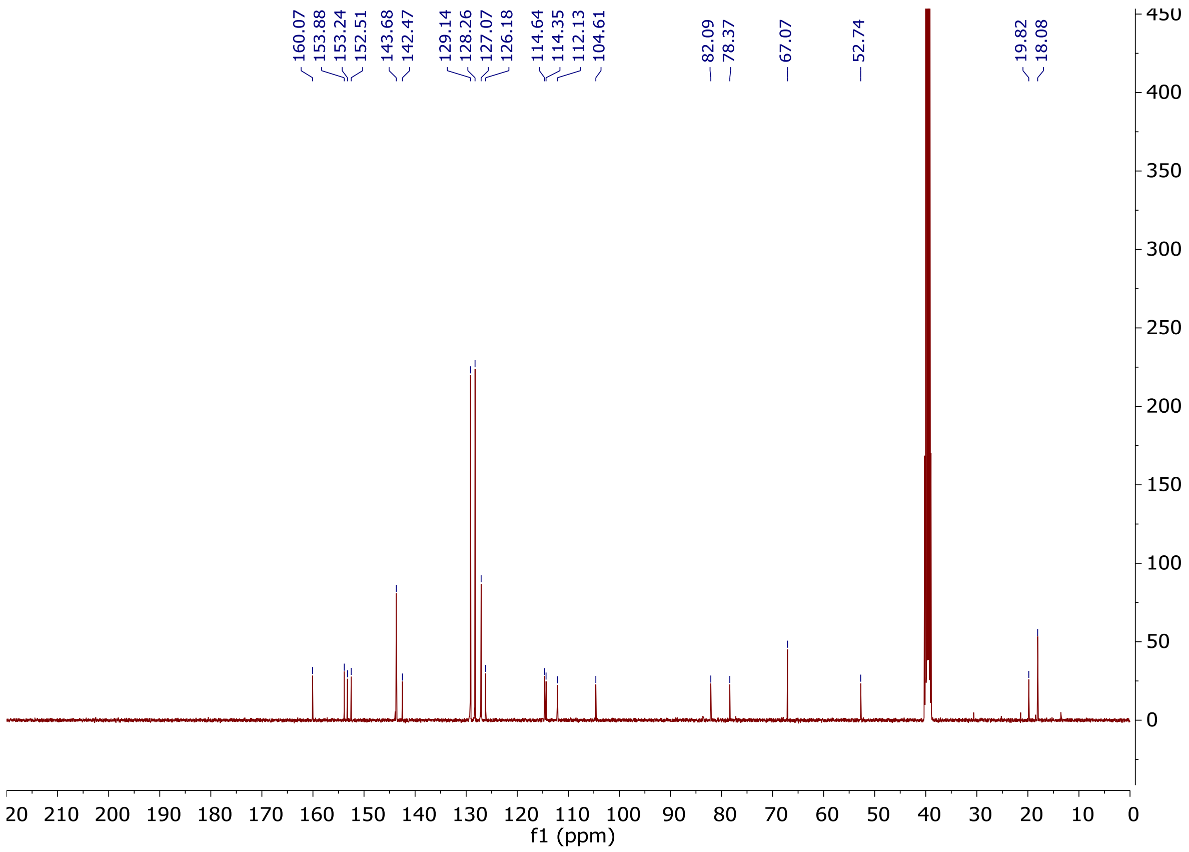
160.07
153.88
153.24
152.51
143.68
142.47
129.14
128.26
127.07
126.18
114.64
114.35
112.13
104.61
82.09
78.37
67.07
52.74
19.82
18.08
f1 (ppm)

5-(methylthio)pent-2-yn-1-ol (11a)

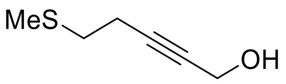

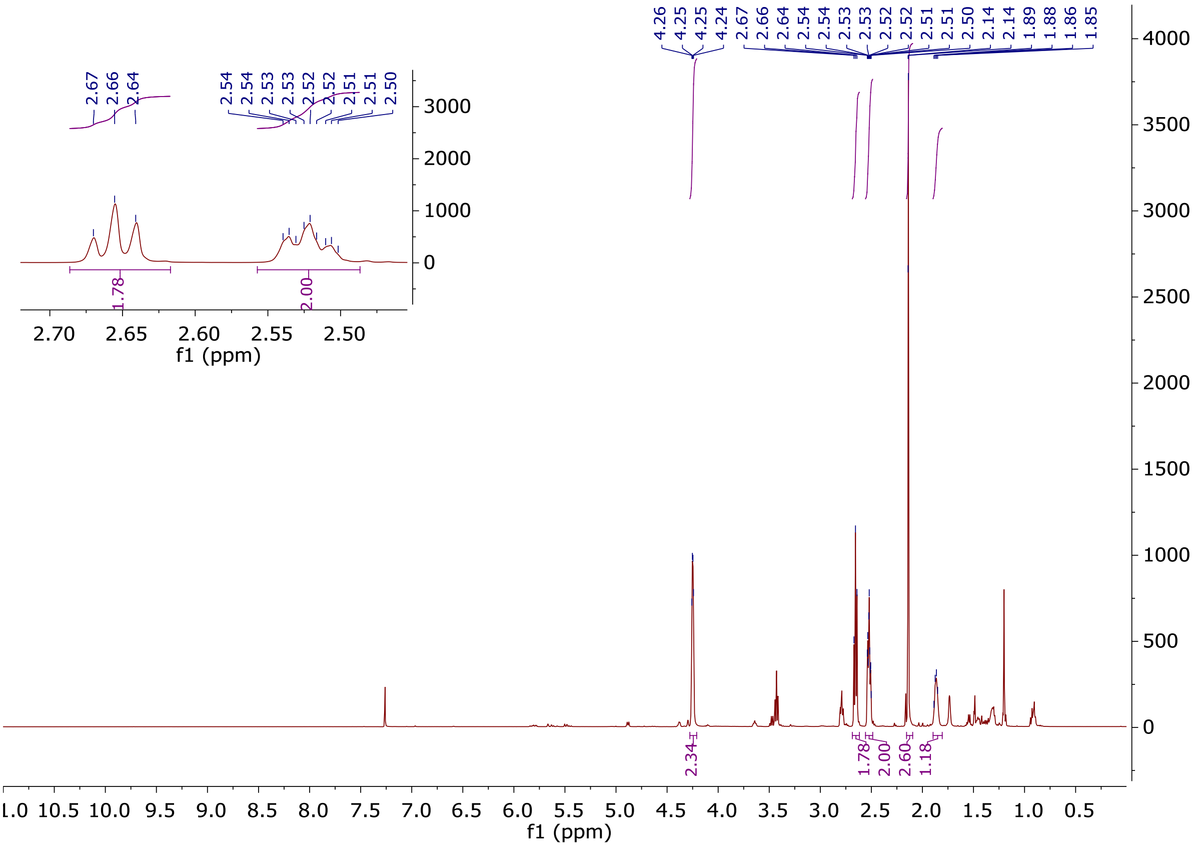

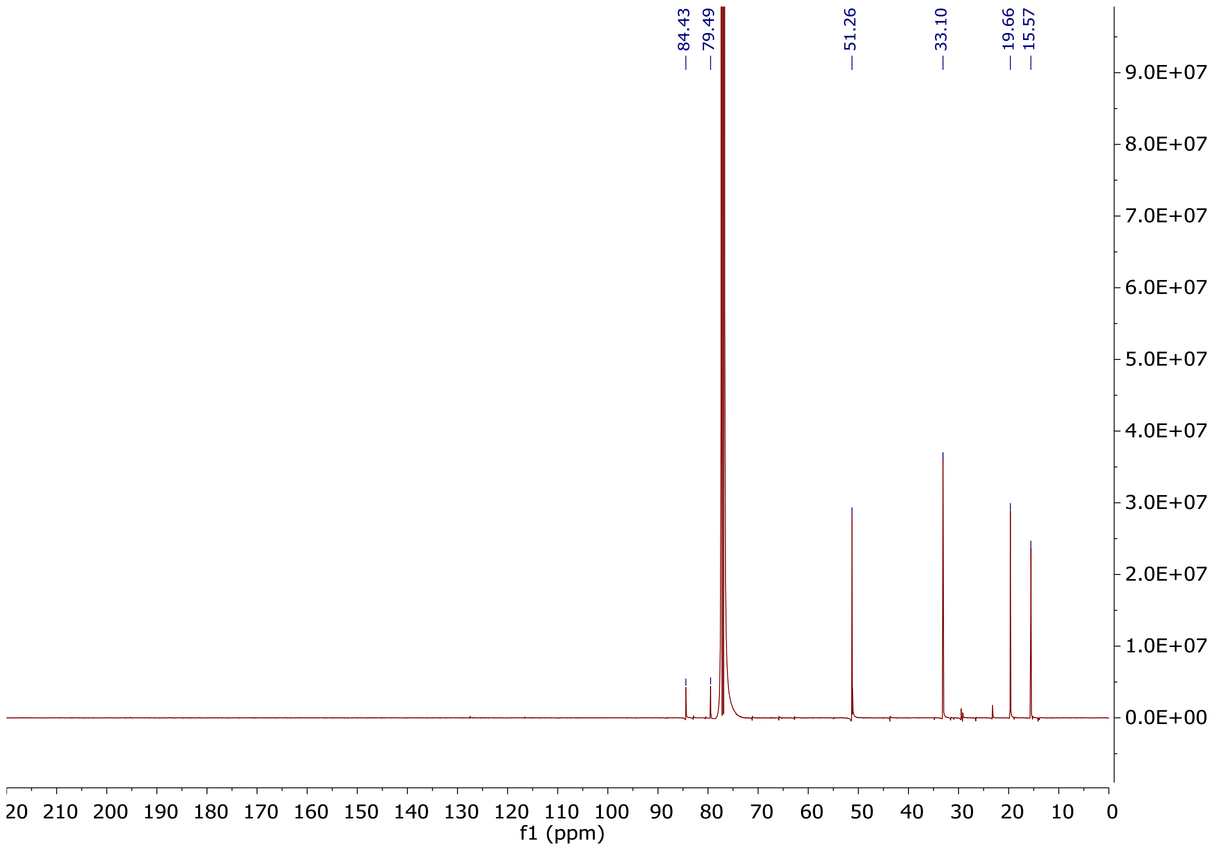
84.43
79.49
51.26
33.10
19.66
15.57
9.0E+07
8.0E+07
7.0E+07
6.0E+07
5.0E+07
4.0E+07
3.0E+07
2.0E+07
1.0E+07
0.0E+00
20 210 200 190 180 170 160 150 140 130 120 110 100 90 80 70 60 50 40 30 20 10 0
f1 (ppm)

5-(methylthio)pent-2-yn-1-yl (4-methylcoumarin)carbamate (11)

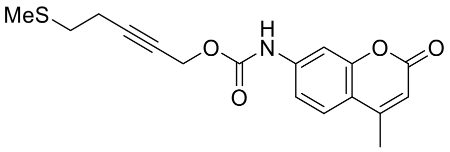

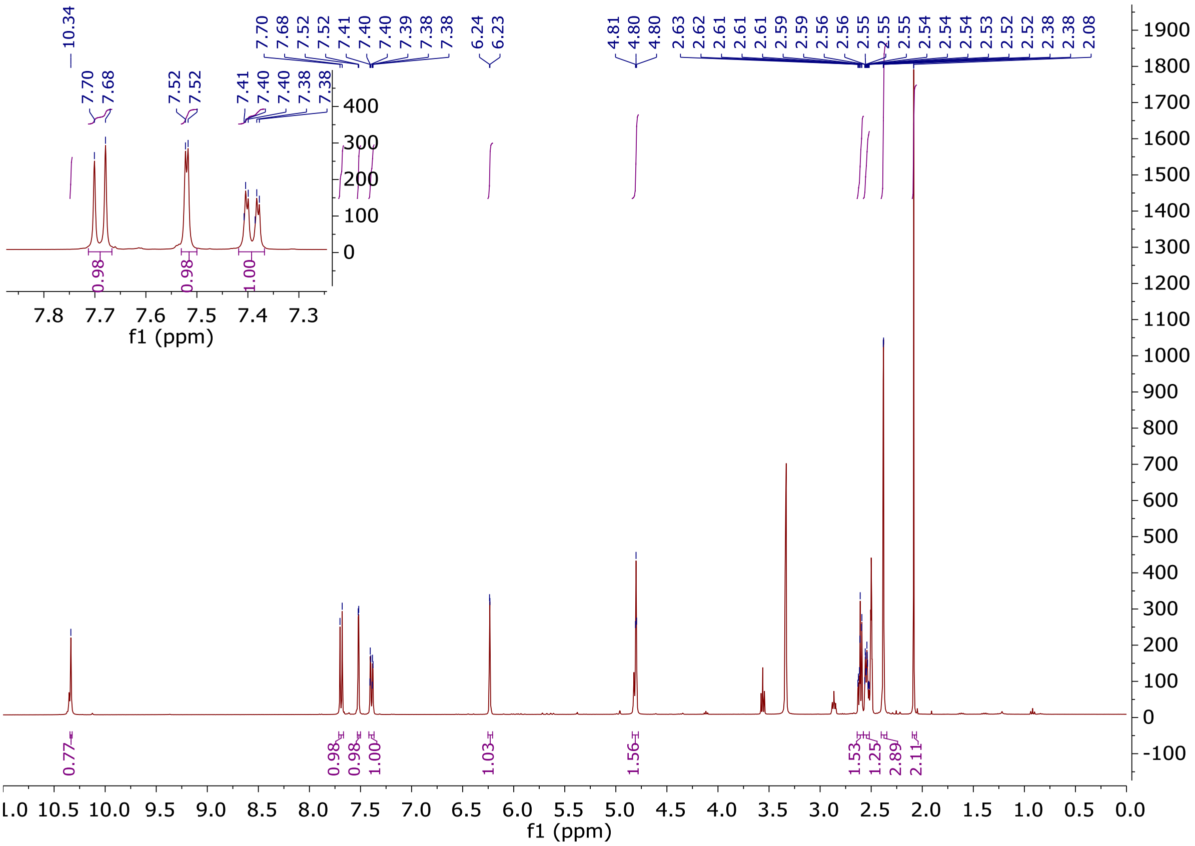

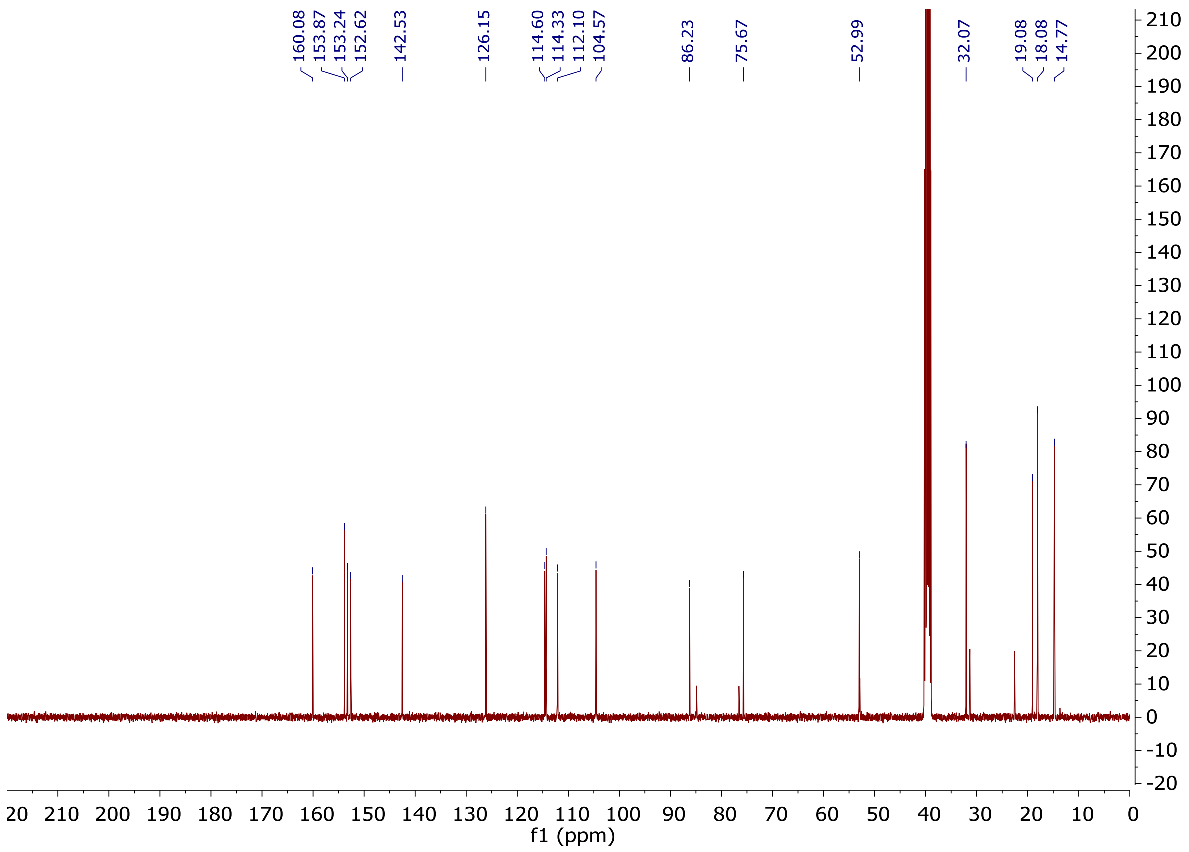
160.08
153.87
153.24
152.62
142.53
126.15
114.60
114.33
112.10
104.57
86.23
75.67
52.99
32.07
19.08
18.08
14.77
f1 (ppm)

But-3-yn-1-yl(trityl)sulfane (12a)

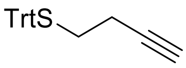

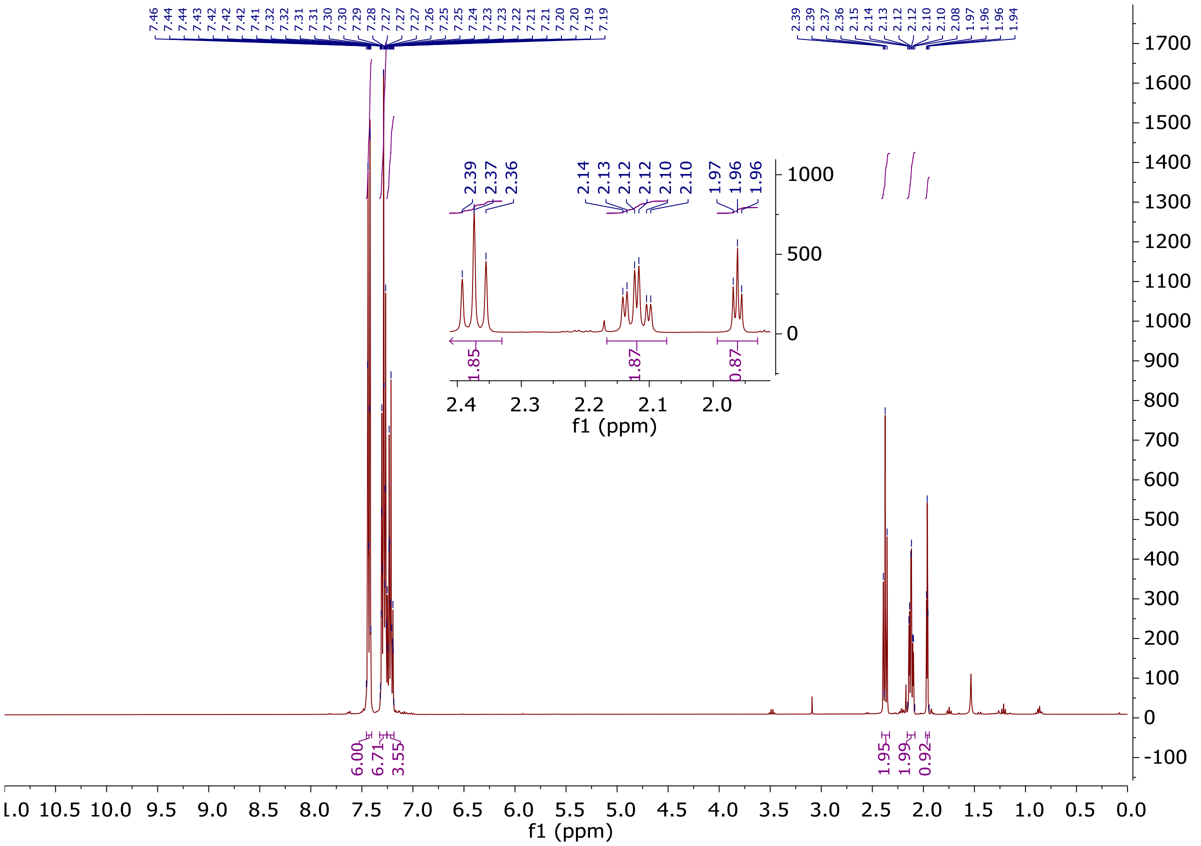

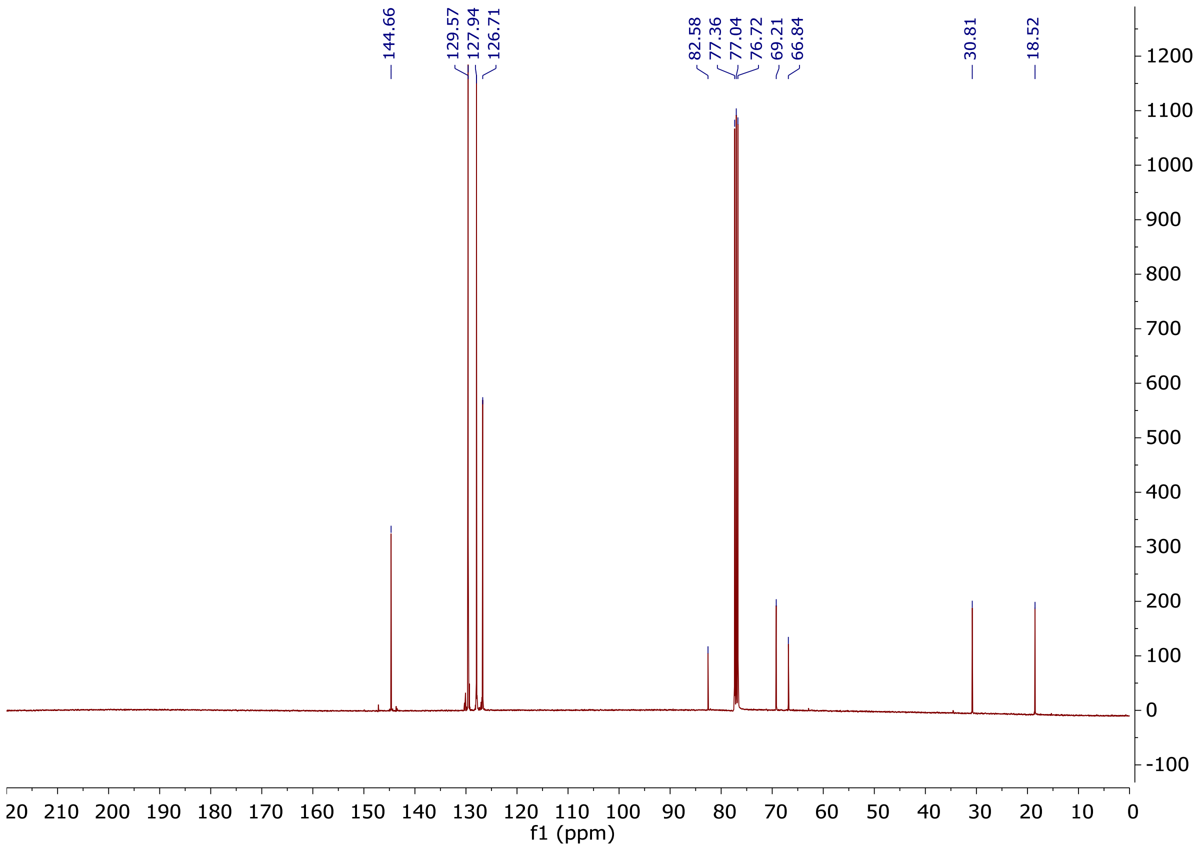
144.66
129.57
127.94
126.71
82.58
77.36
77.04
76.72
69.21
66.84
30.81
18.52
1200
1100
1000
900
800
700
600
500
400
300
200
100
0
-100
20 210 200 190 180 170 160 150 140 130 120 110 100 90 80 70 60 50 40 30 20 10 0
f1 (ppm)

5-(tritylthio)pent-2-yn-1-ol (12b)

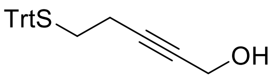

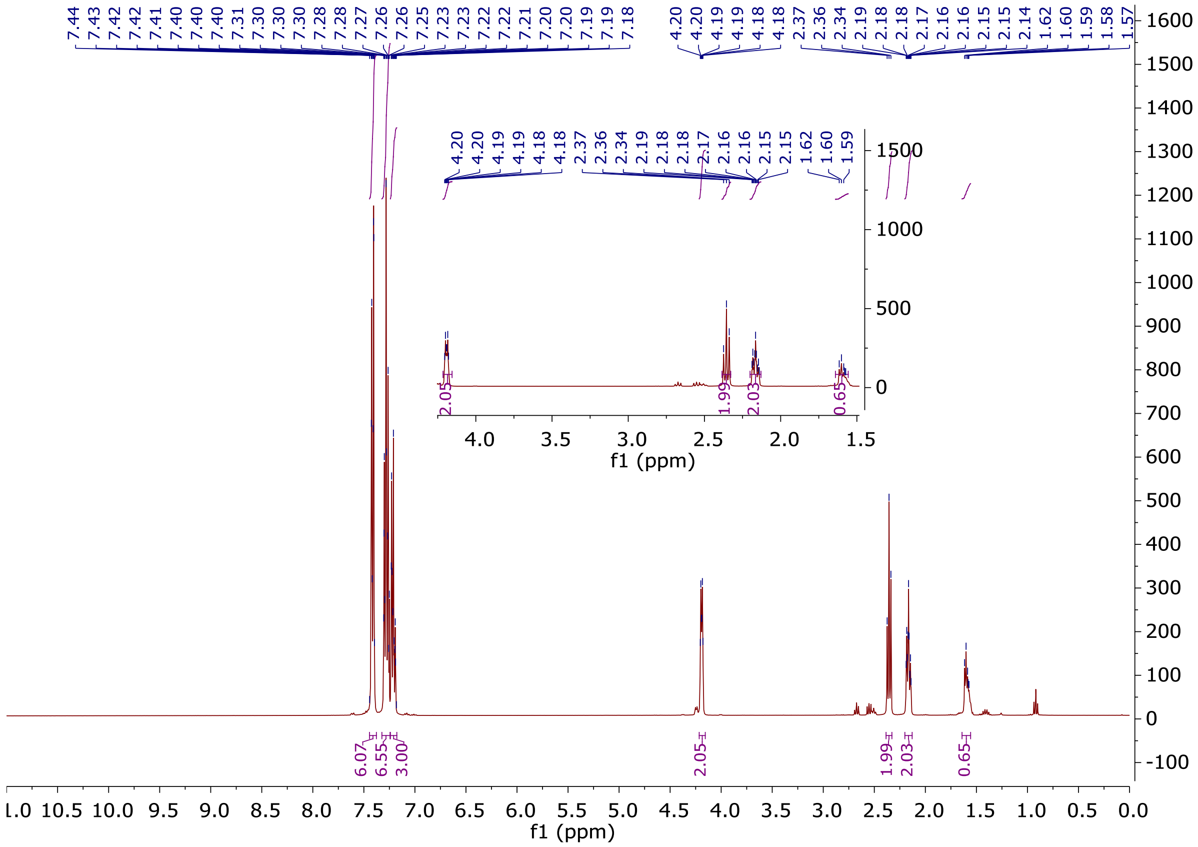

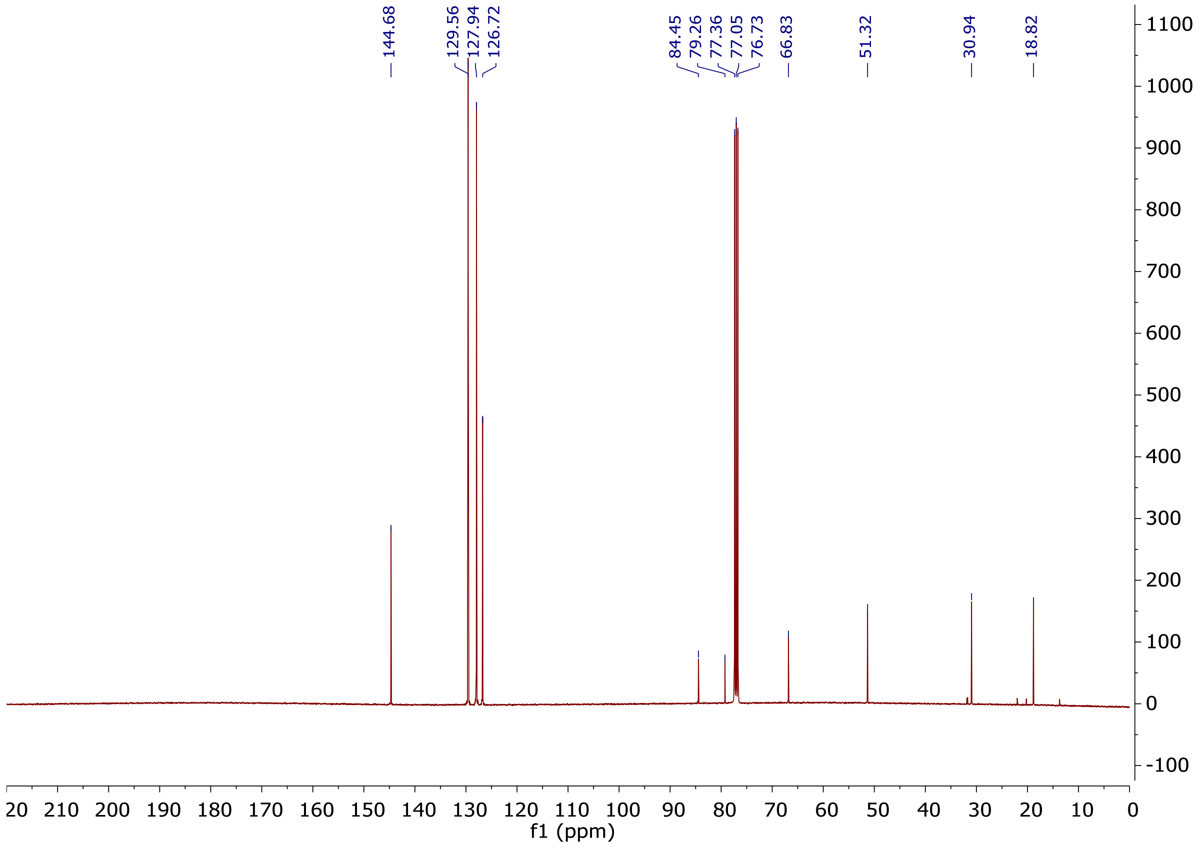

5-(tritylthio)pent-2-yn-1-yl (4-methylcoumarin)carbamate (12)

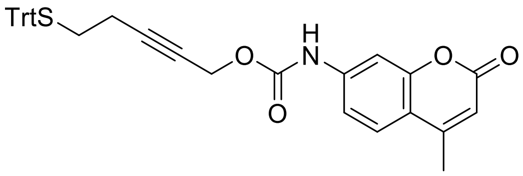

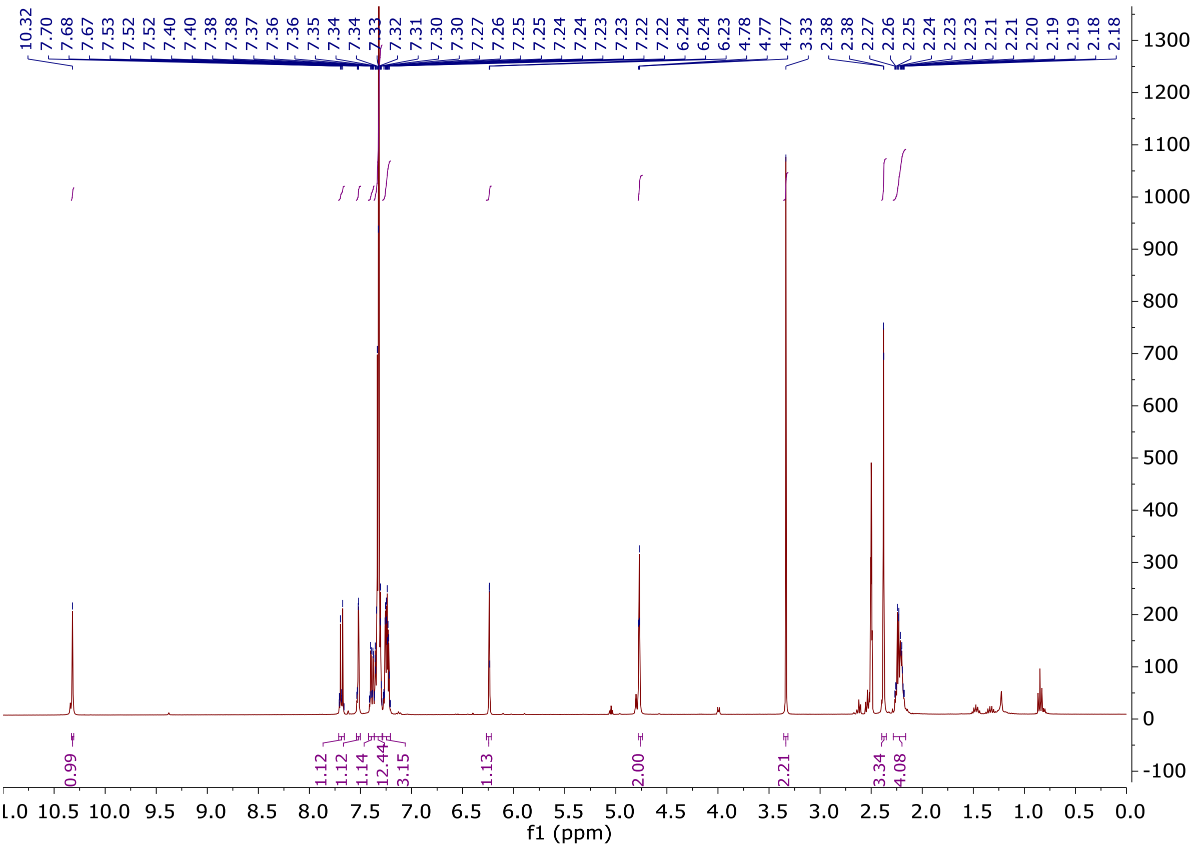

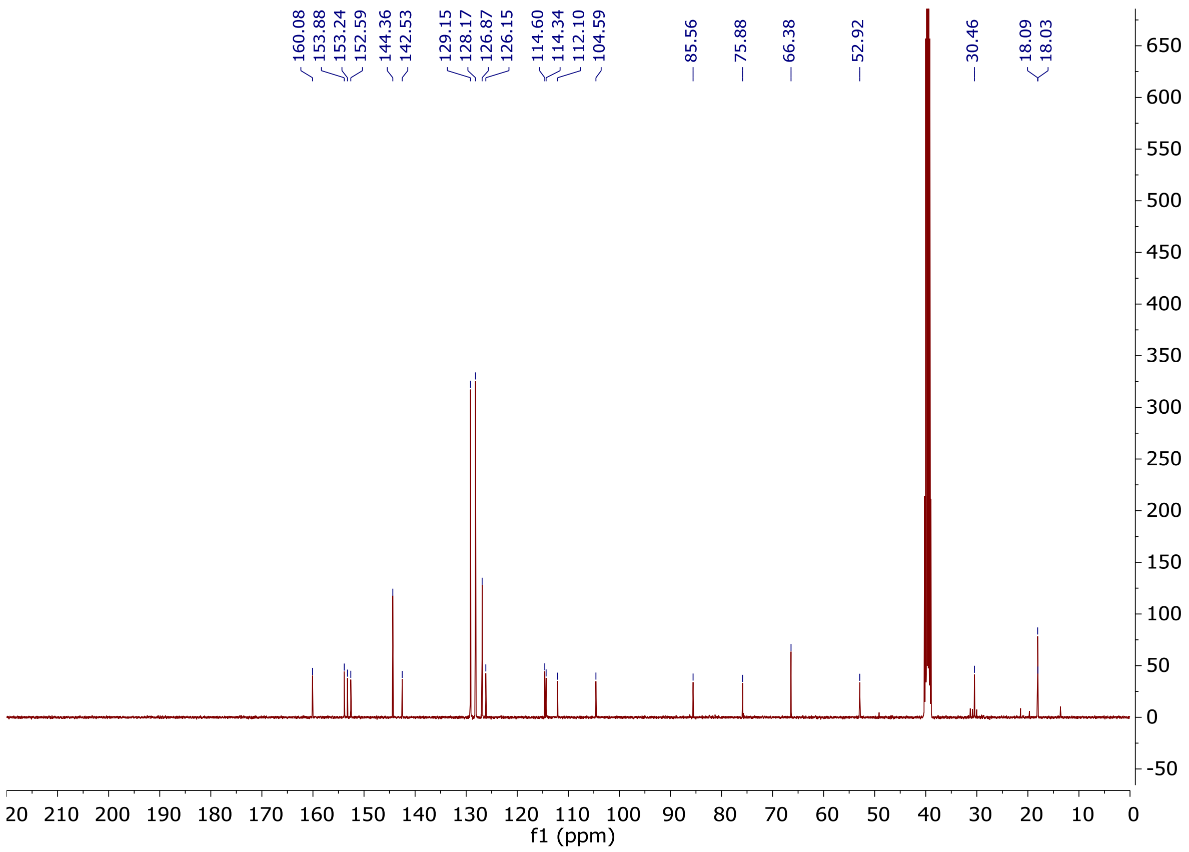
160.08
153.88
153.24
152.59
144.36
142.53
129.15
128.17
126.87
126.15
114.60
114.34
112.10
104.59
85.56
75.88
66.38
52.92
30.46
18.09
18.03
f1 (ppm)

3,6,9-trioxa-12-thiahexadec-14-yn-16-ol (13d)

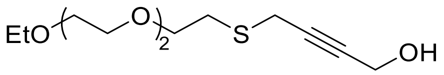

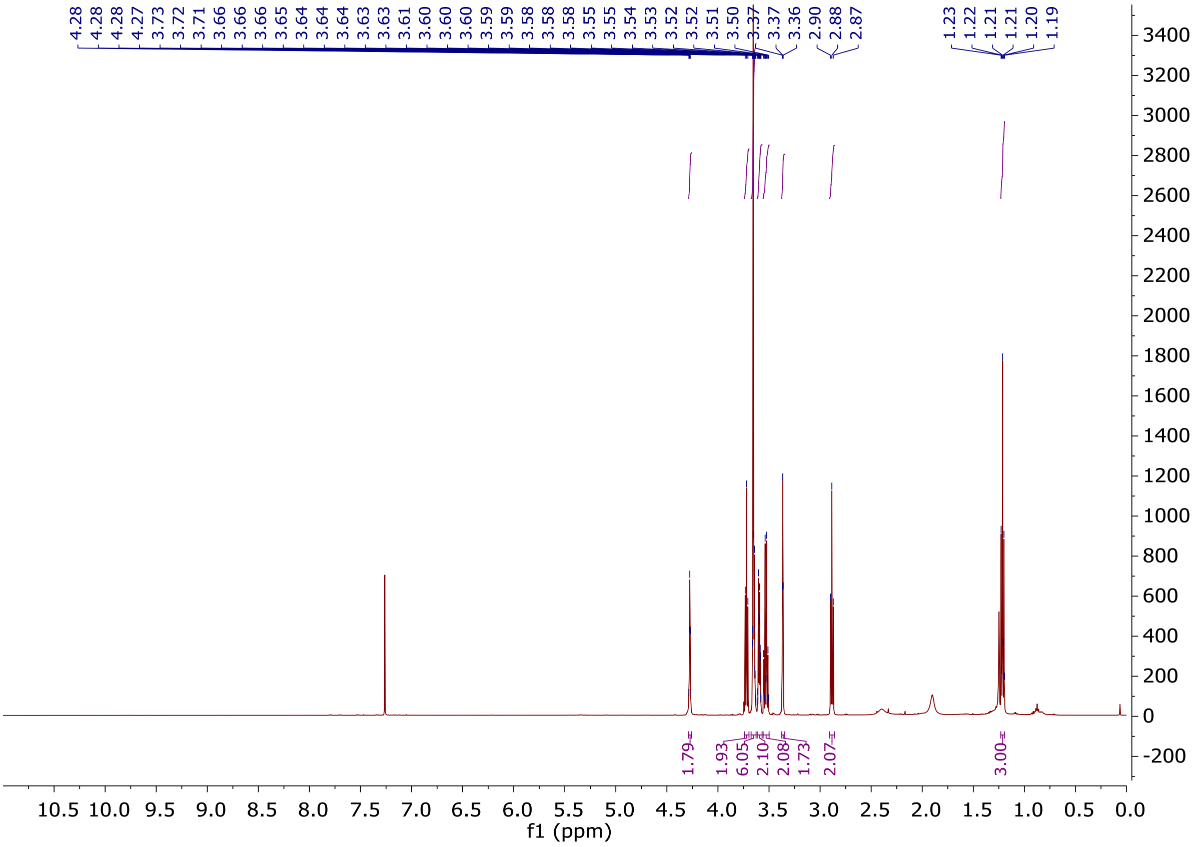

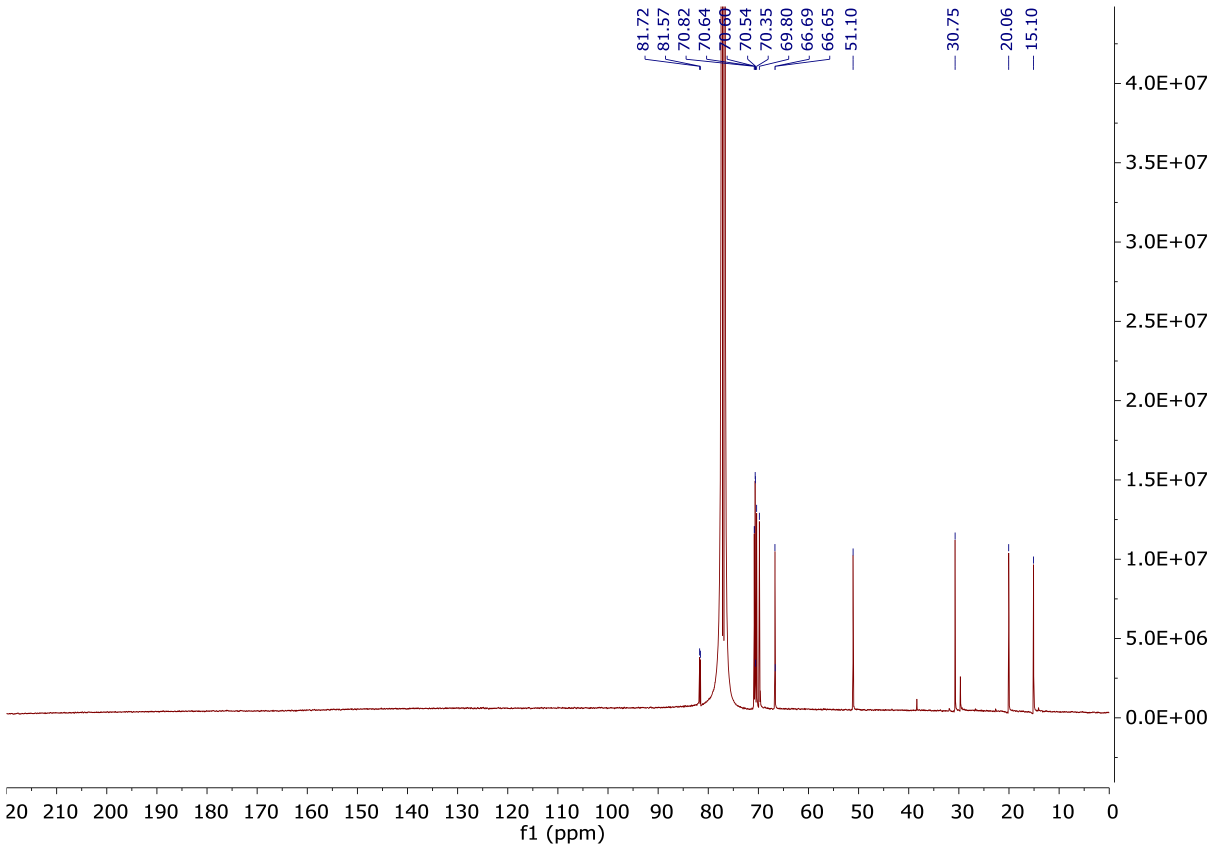
81.72
81.57
70.82
70.64
70.60
70.54
70.35
69.80
66.69
66.65
51.10
30.75
20.06
15.10
4.0E+07
3.5E+07
3.0E+07
2.5E+07
2.0E+07
1.5E+07
1.0E+07
5.0E+06
0.0E+00
20 210 200 190 180 170 160 150 140 130 120 110 100 90 80 70 60 50 40 30 20 10 0
f1 (ppm)

3,6,9-trioxa-12-thiahexadec-14-yn-16-yl (4-methylcoumarin)carbamate (13)

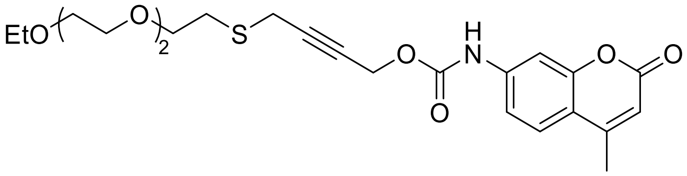

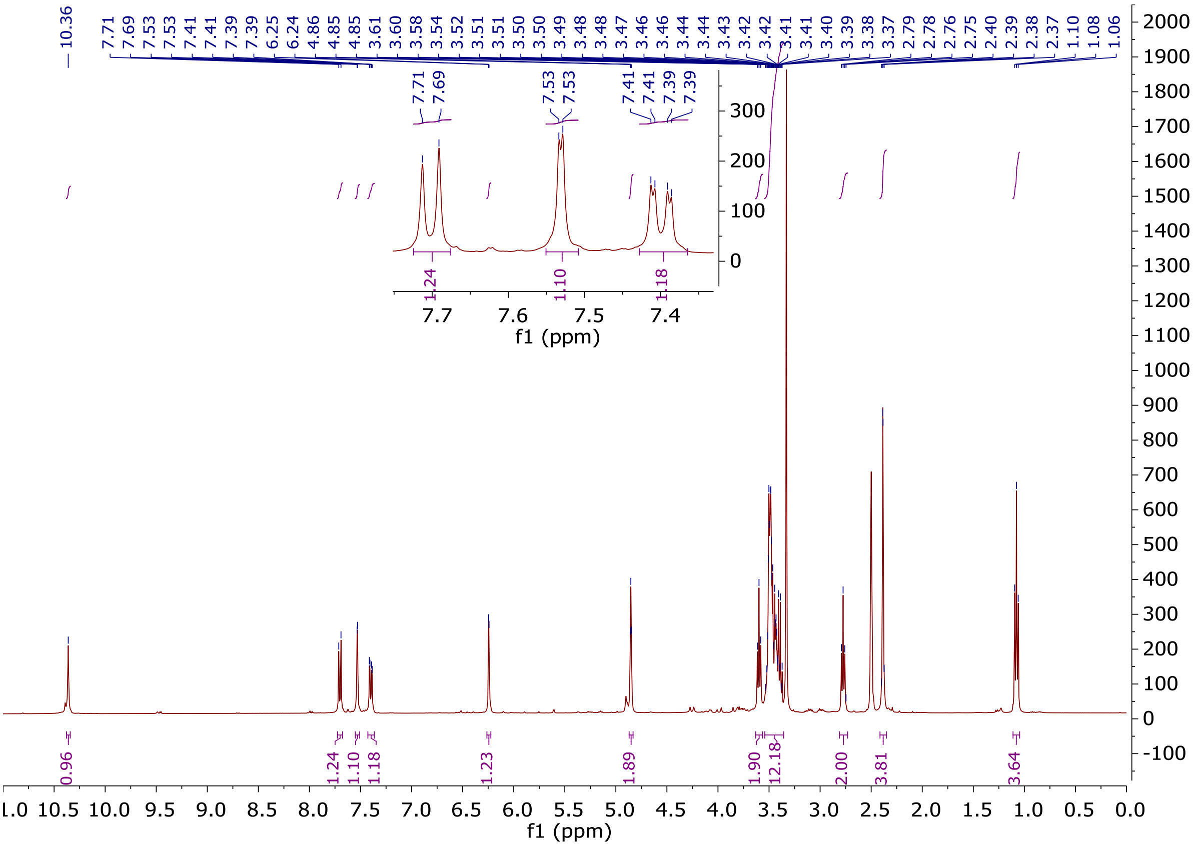

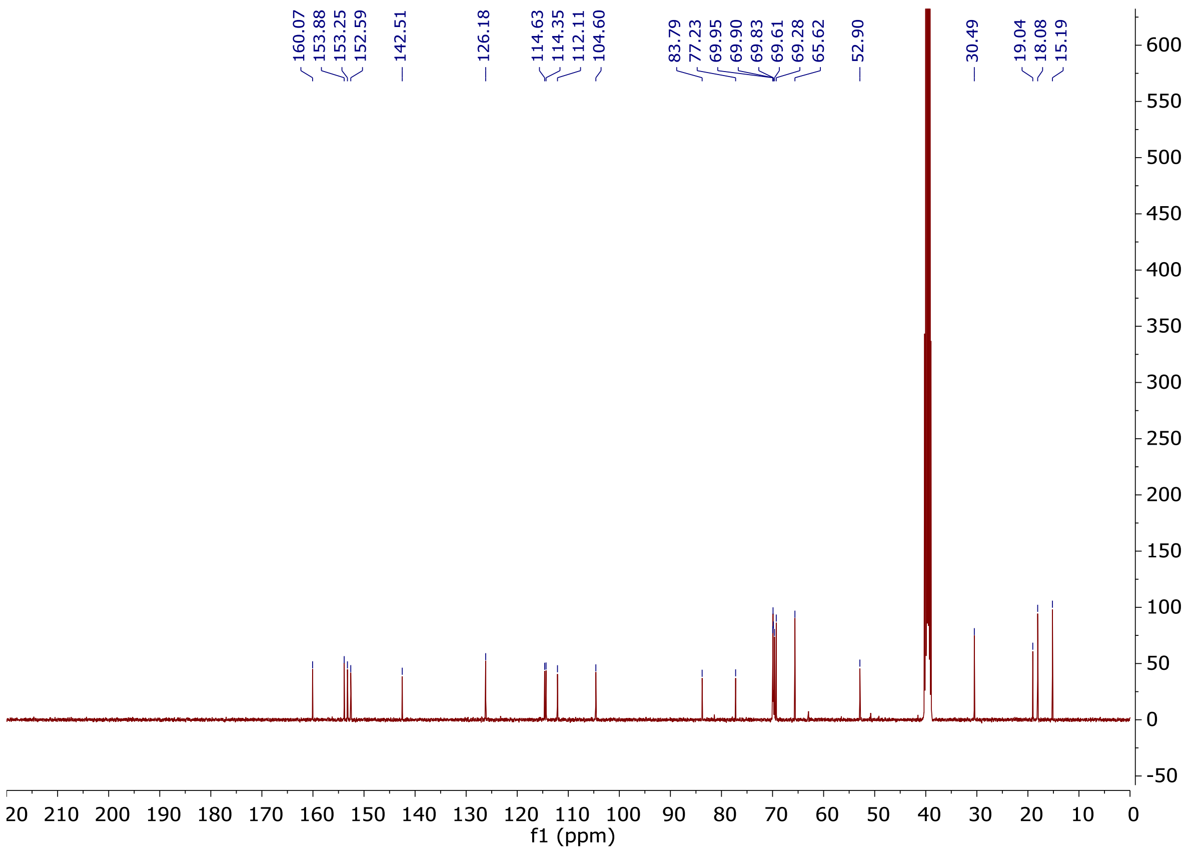
160.07
153.88
153.25
152.59
142.51
126.18
114.63
114.35
112.11
104.60
83.79
77.23
69.95
69.90
69.83
69.61
69.28
65.62
52.90
30.49
19.04
18.08
15.19
f1 (ppm)

3,6,9-trioxa-12-thiahexadec-14-yn-16-yl (4-nitrophenyl) carbonate (15a)

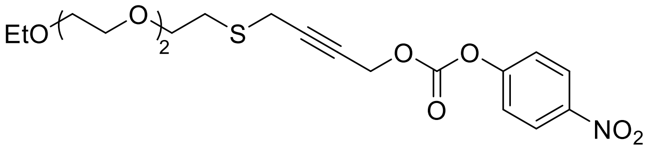

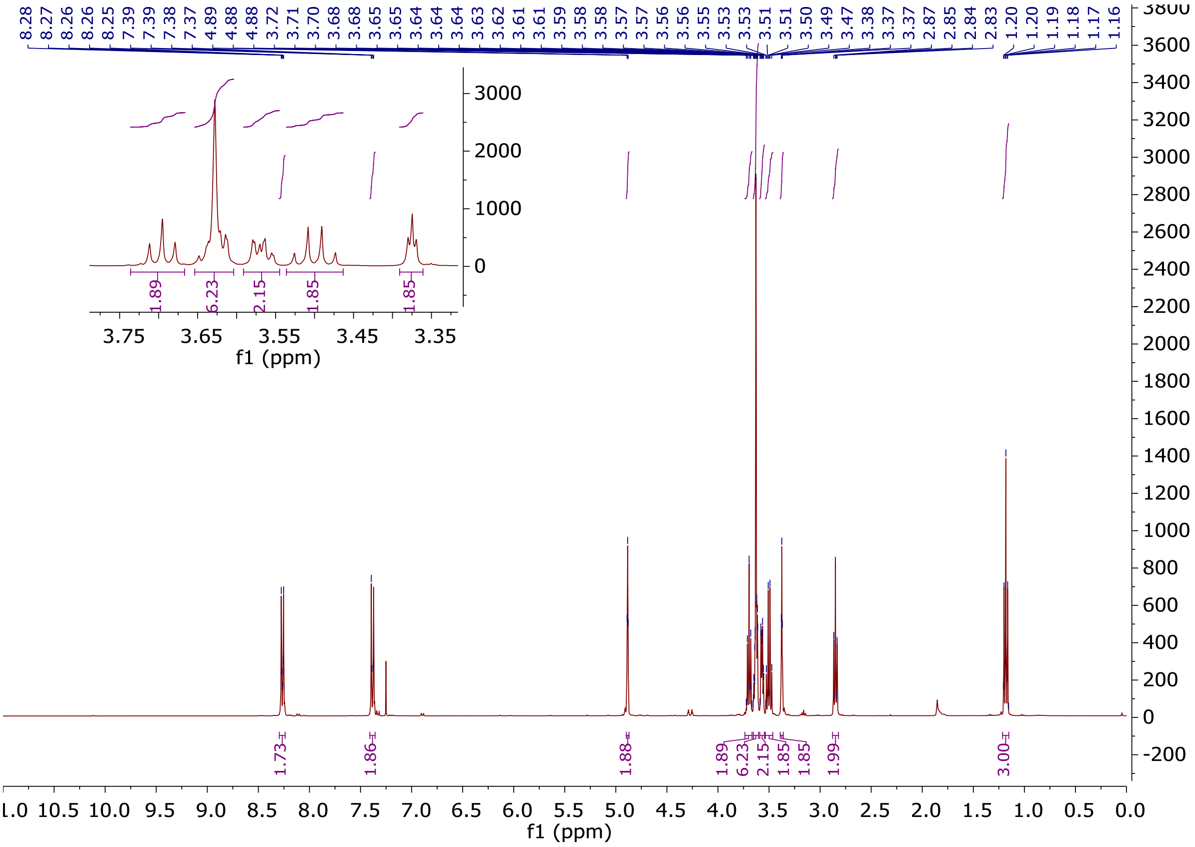

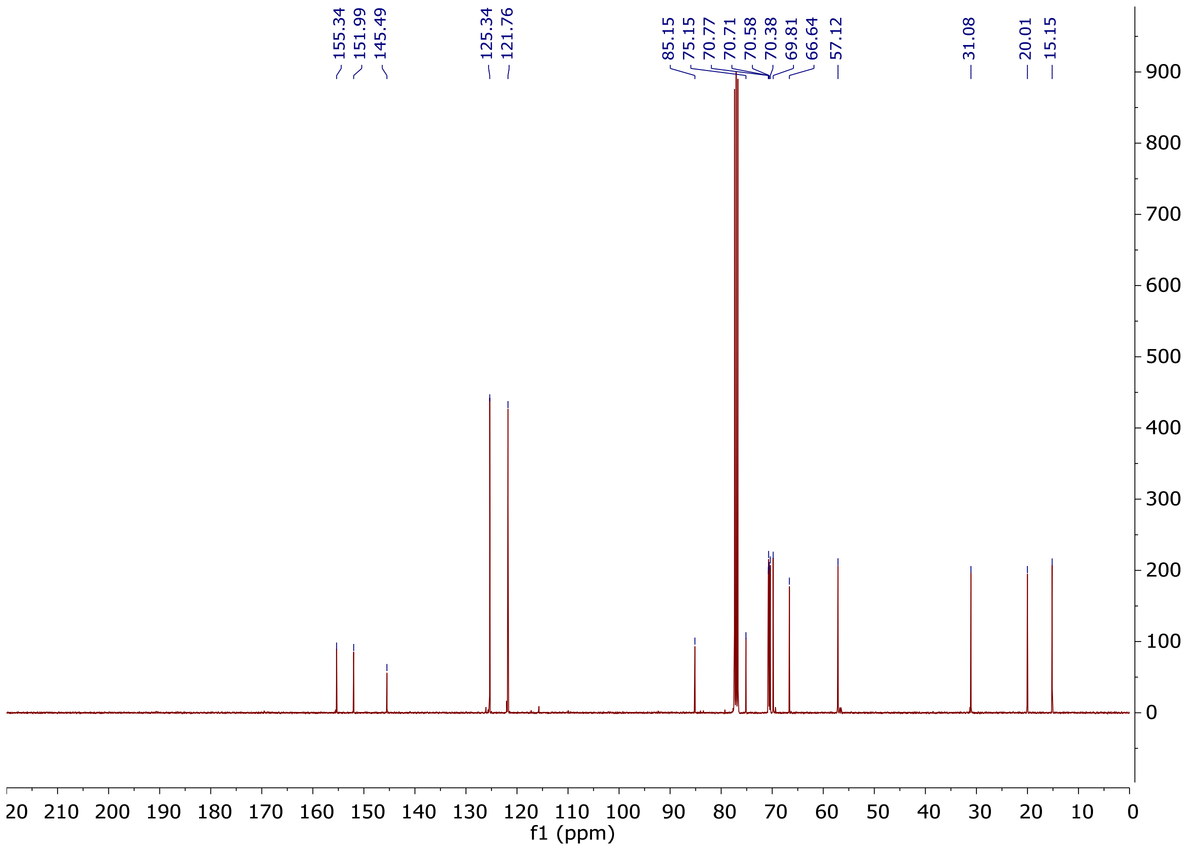
155.34
151.99
145.49
125.34
121.76
85.15
75.15
70.77
70.71
70.58
70.38
69.81
66.64
57.12
31.08
20.01
15.15
f1 (ppm)

cDox (15)

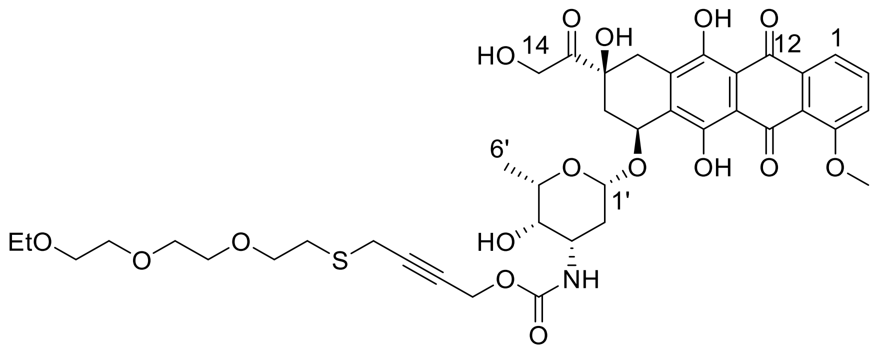

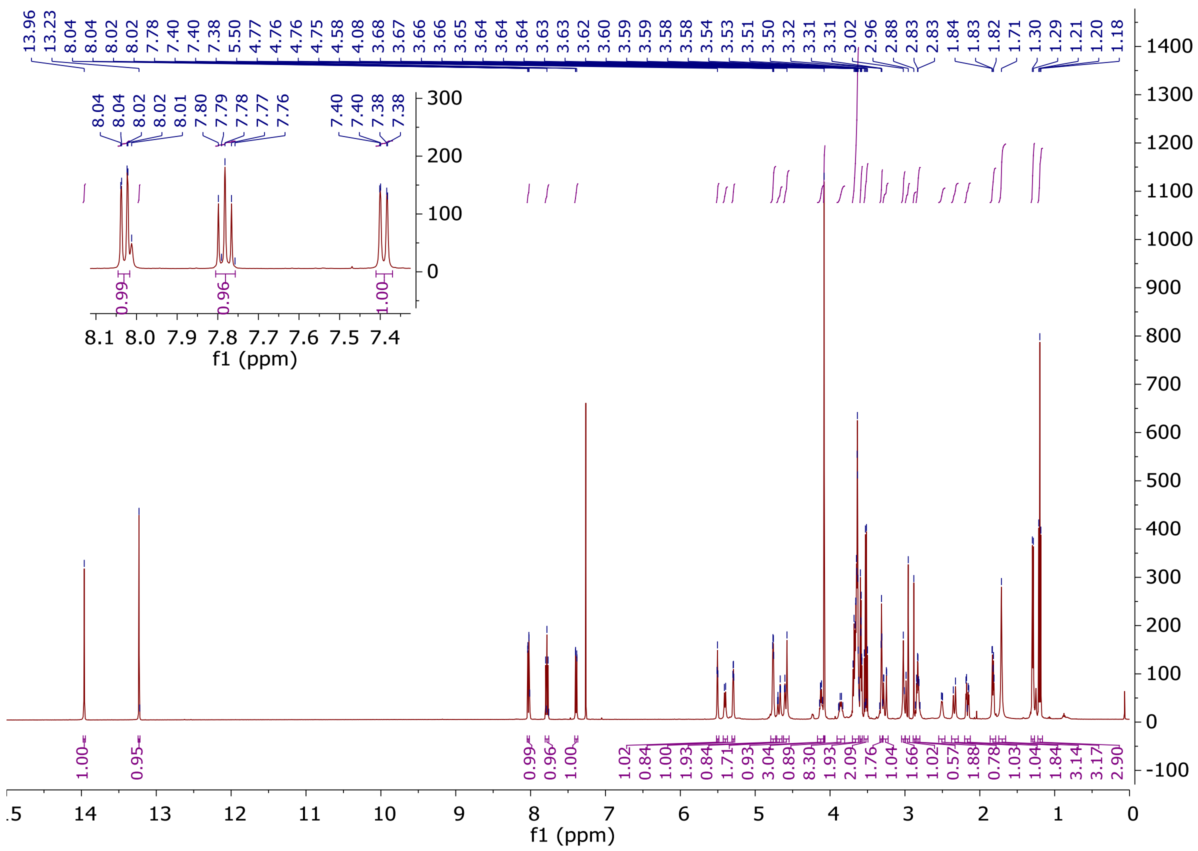

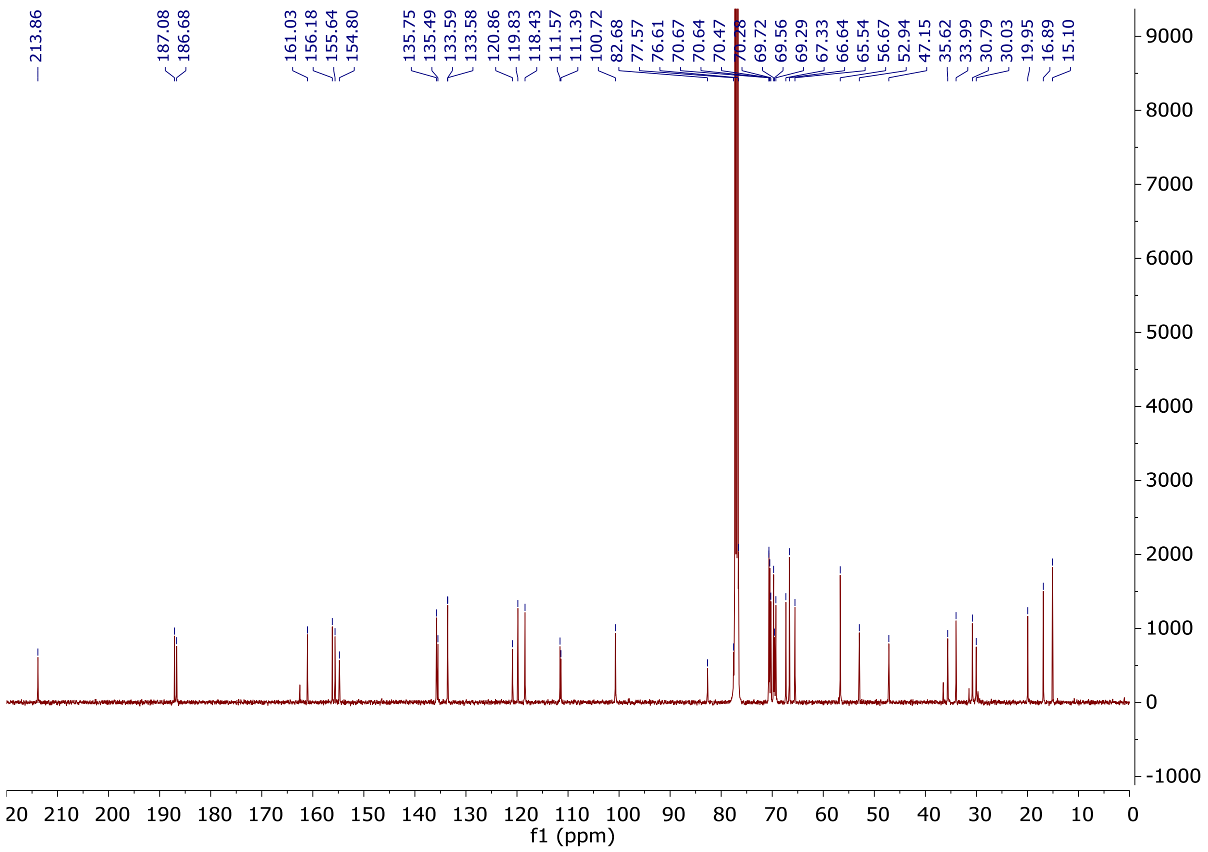
213.86
187.08
186.68
161.03
156.18
155.64
154.80
135.75
135.49
133.59
133.58
120.86
119.83
118.43
111.57
111.39
100.72
82.68
77.57
76.61
70.67
70.64
70.47
70.26
69.72
69.56
69.29
67.33
66.64
65.54
56.67
52.94
47.15
35.62
33.99
30.79
30.03
19.95
16.89
15.10
f1 (ppm)

tert-butyl (16-hydroxy-3,6,9-trioxa-12-thiahexadec-14-yn-1-yl)carbamate (16d)

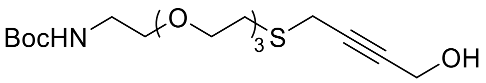

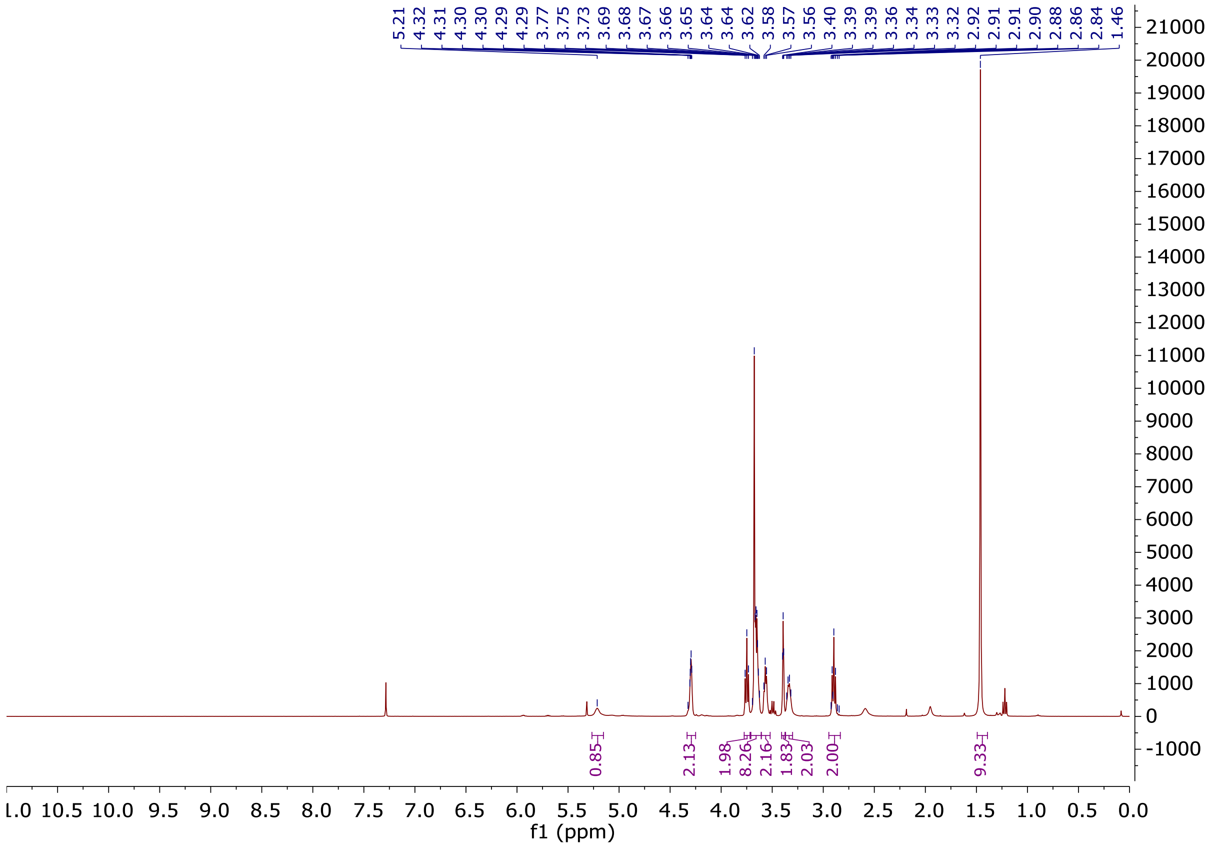

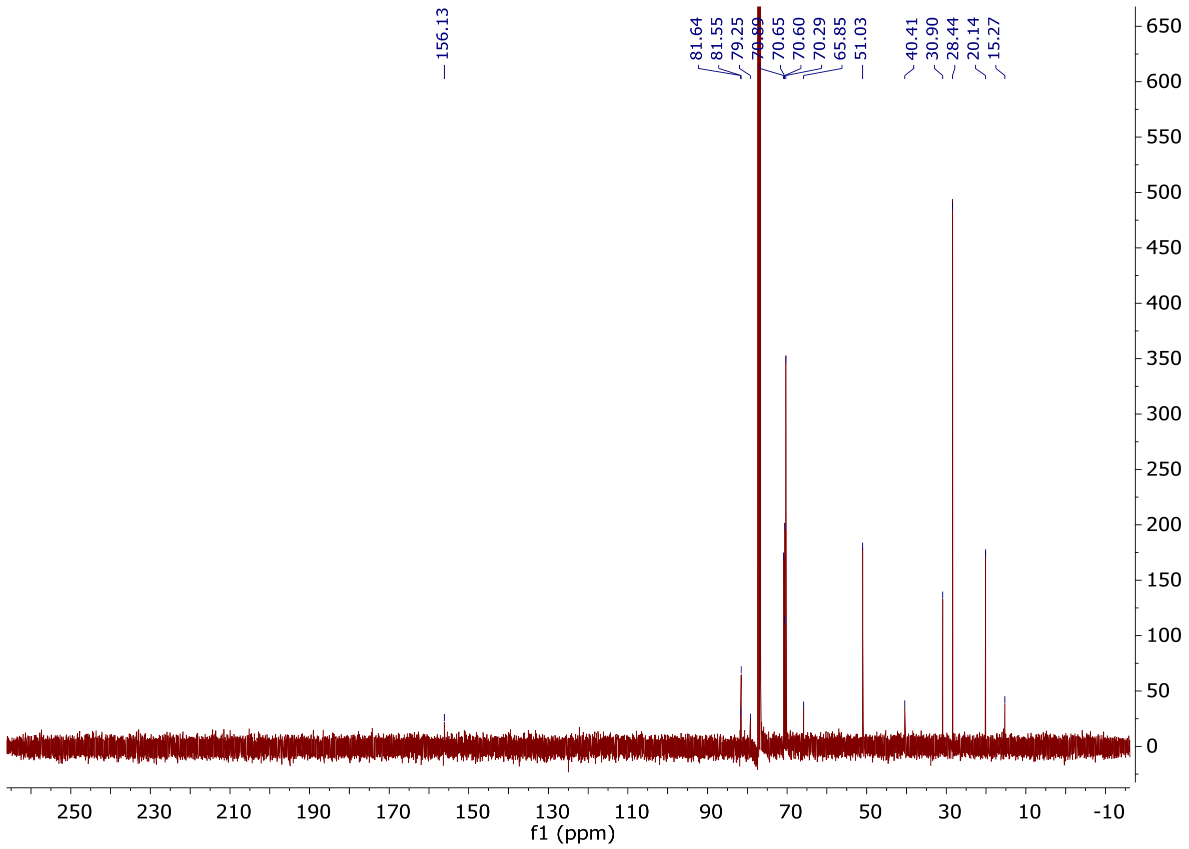

156.13
81.64
81.55
79.25
70.89
70.65
70.60
70.29
65.85
51.03
40.41
30.90
28.44
20.14
15.27
f1 (ppm)

(*E*)-*N*-(16-hydroxy-3,6,9-trioxa-12-thiahexadec-14-yn-1-yl)-4-oxo-4-phenylbut-2-enamide (16e)

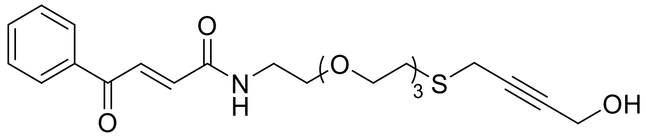

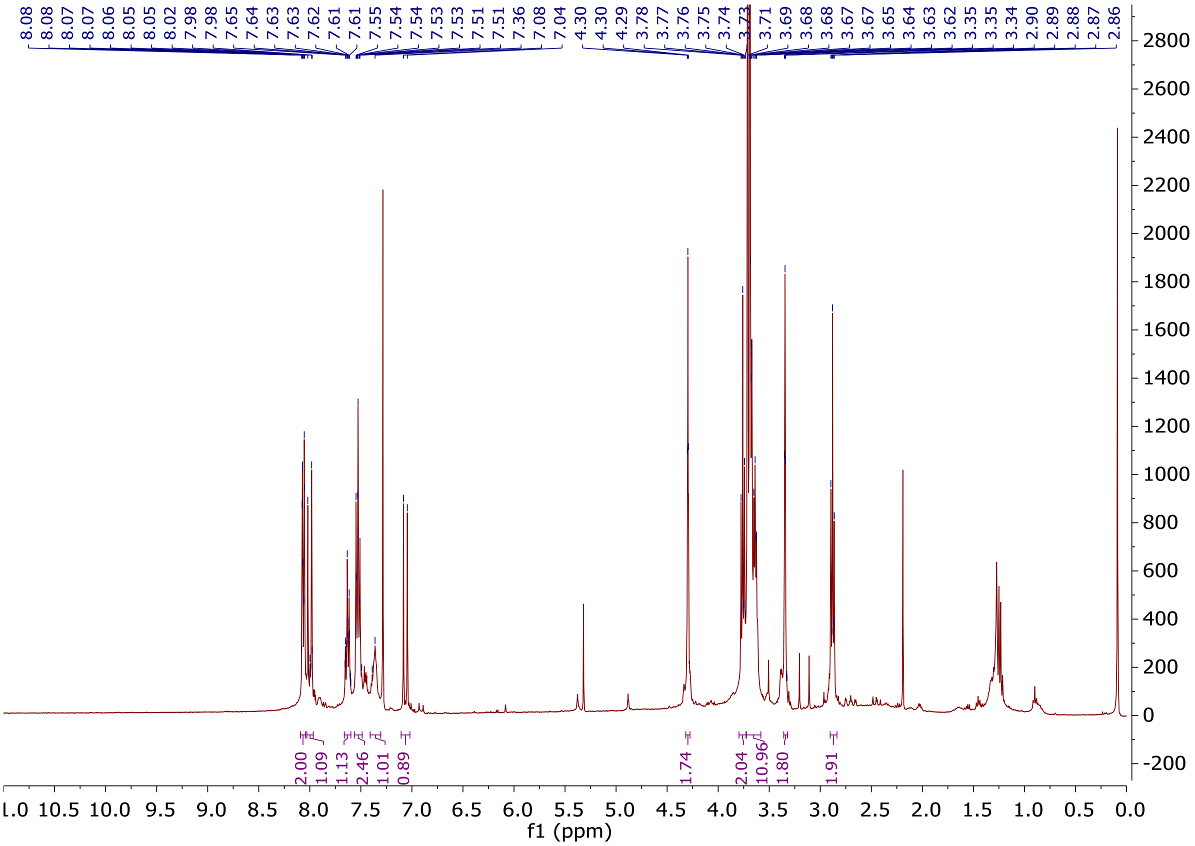

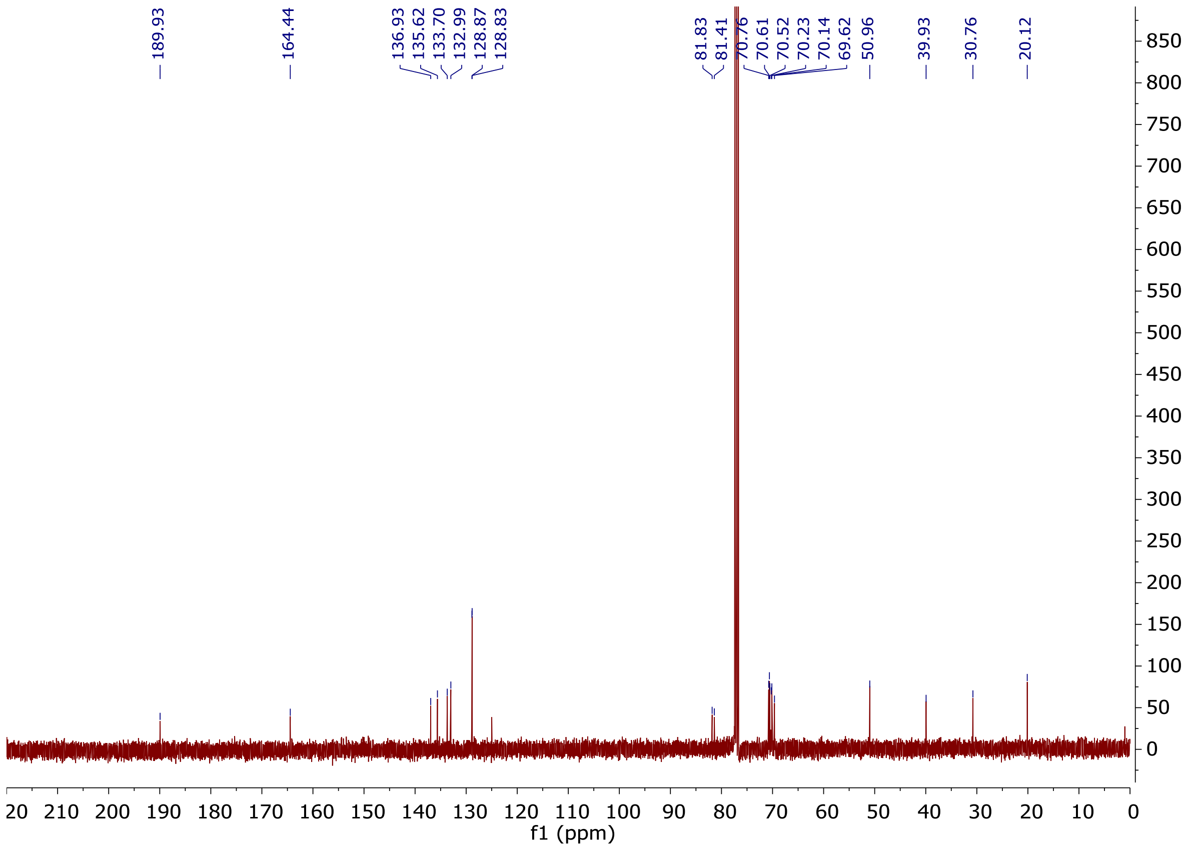
189.93
164.44
136.93
135.62
133.70
132.99
128.87
128.83
81.83
81.41
70.76
70.61
70.52
70.23
70.14
69.62
50.96
39.93
30.76
20.12
f1 (ppm)

(*E*)-*N*-(16-hydroxy-3,6,9-trioxa-12-thiahexadec-14-yn-1-yl)-4-oxo-4-phenylbut-2-enamide doxorubicin carbamate (16)

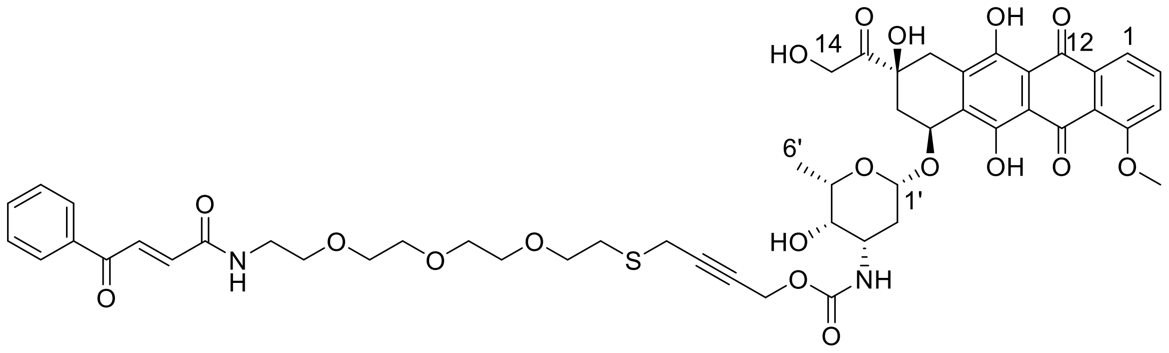

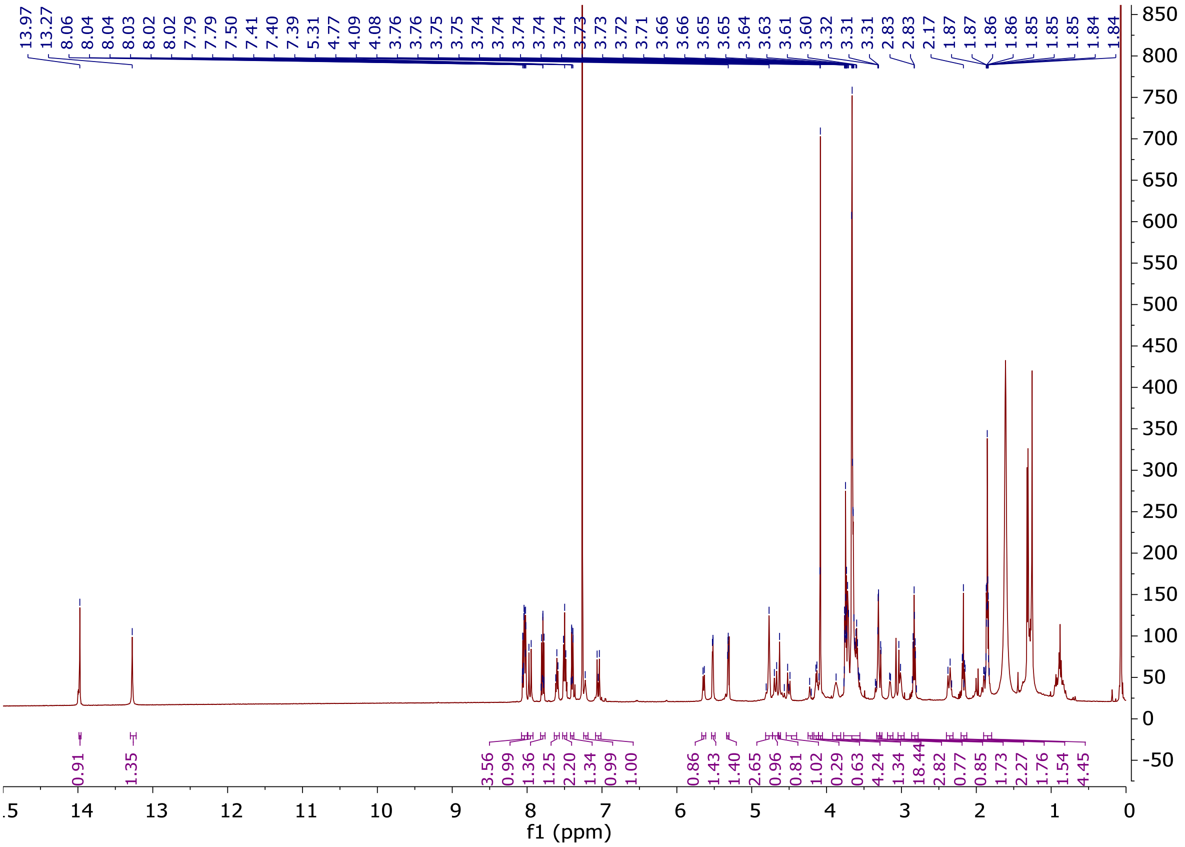

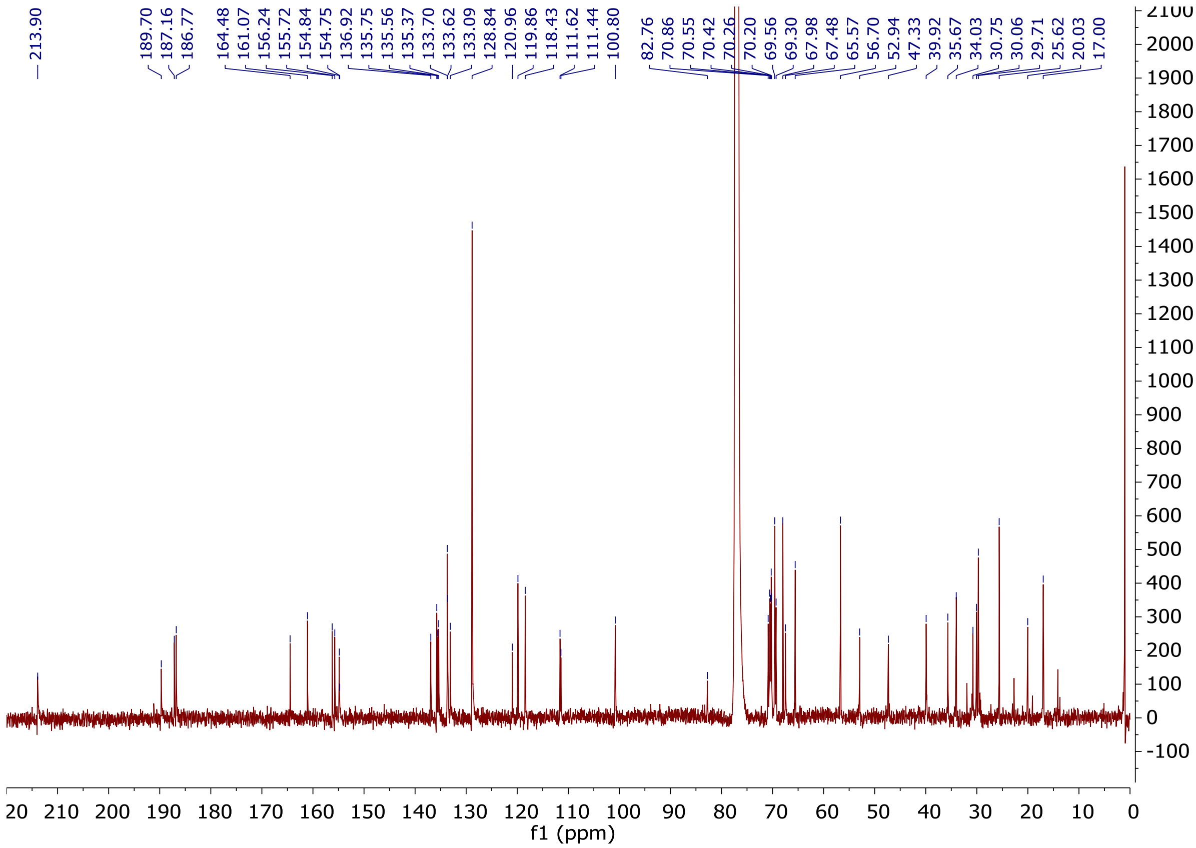
213.90
189.70
187.16
186.77
164.48
161.07
156.24
155.72
154.84
154.75
136.92
135.75
135.56
135.37
133.70
133.62
133.09
128.84
120.96
119.86
118.43
111.62
111.44
100.80
82.76
70.86
70.55
70.42
70.26
70.20
69.56
69.30
67.98
67.48
65.57
56.70
52.94
47.33
39.92
35.67
34.03
30.75
30.06
29.71
25.62
20.03
17.00
f1 (ppm)

4-(*tert*-butylthio)but-2-yn-1-yl (4-methylcoumarin)carbamate (17)

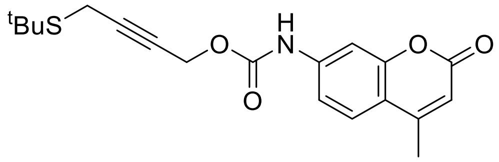

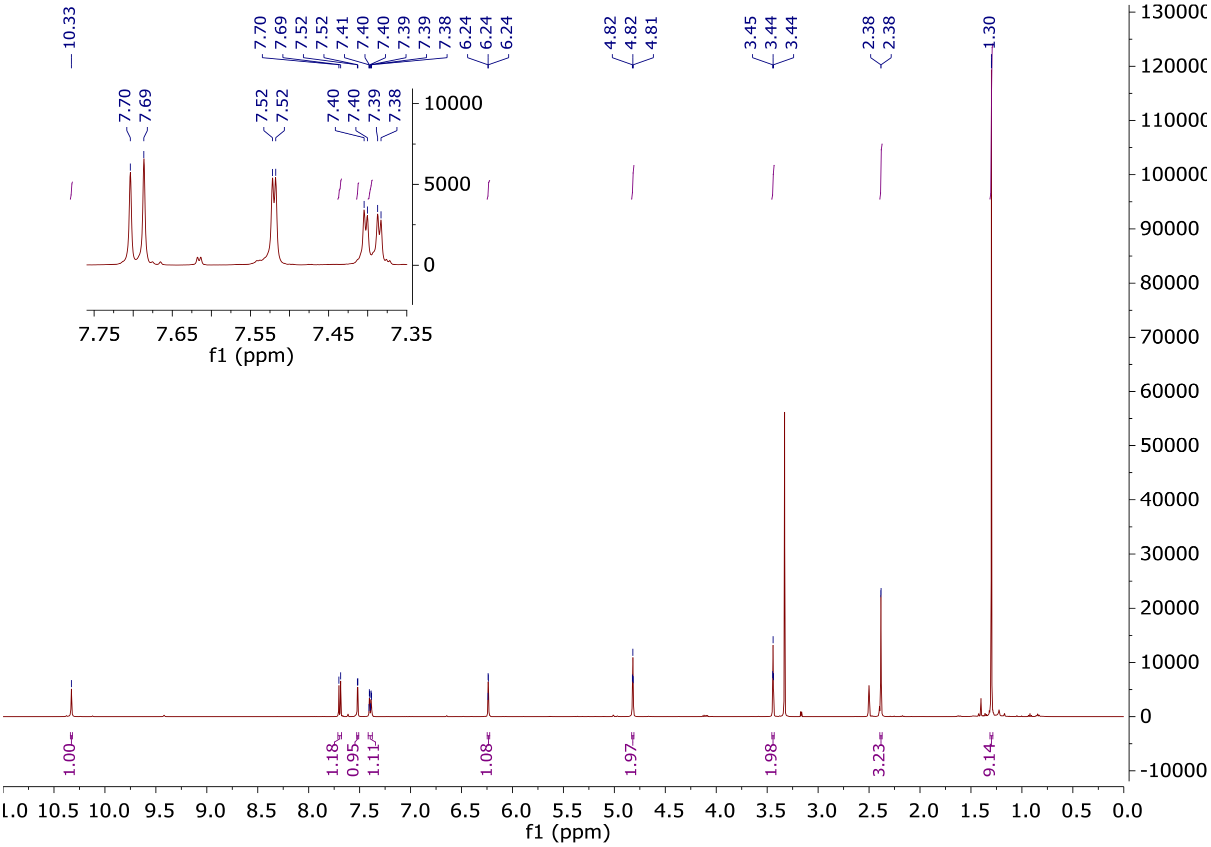

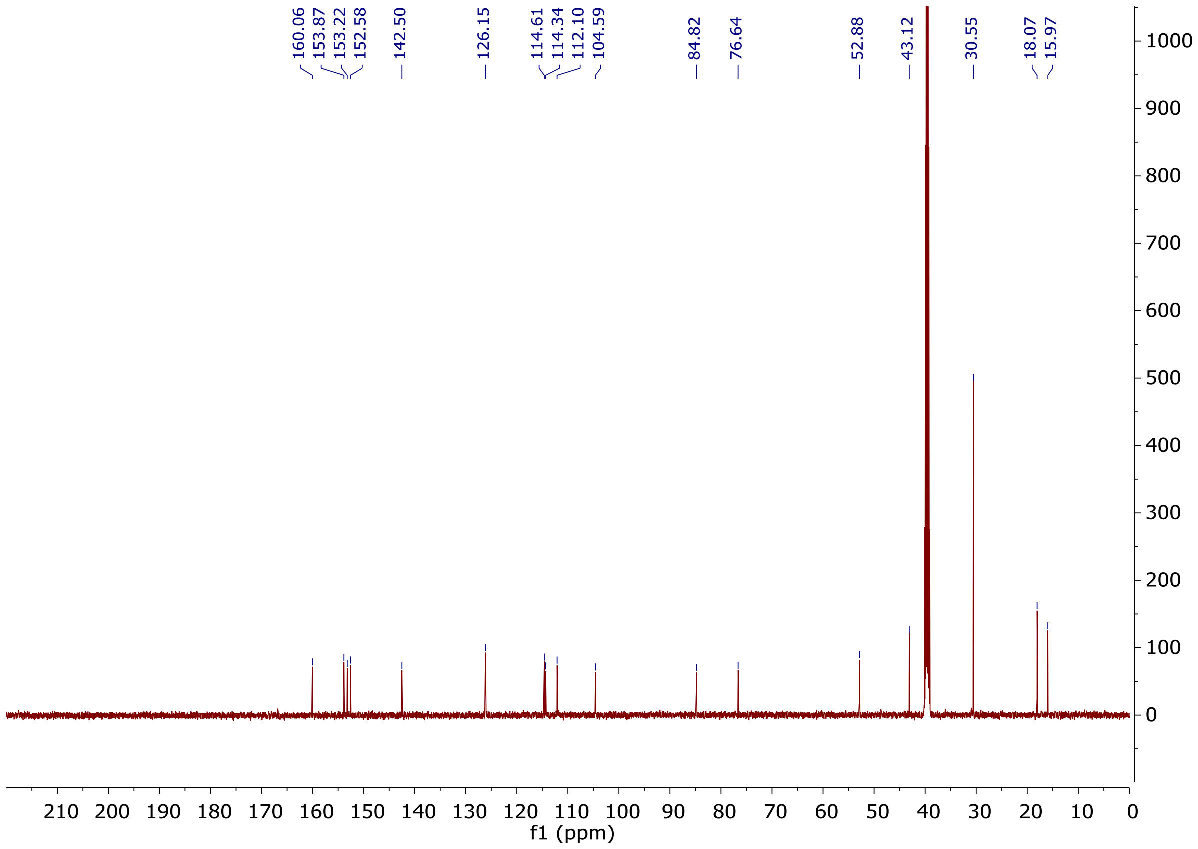
160.06
153.87
153.22
152.58
142.50
126.15
114.61
114.34
112.10
104.59
84.82
76.64
52.88
43.12
30.55
18.07
15.97
1000
900
800
700
600
500
400
300
200
100
0
210 200 190 180 170 160 150 140 130 120 110 100 90 80 70 60 50 40 30 20 10 0
f1 (ppm)

methyl *N*-(*tert*-butoxycarbonyl)-*S*-(4-hydroxybut-2-yn-1-yl)-*L*-cysteinate (18a)

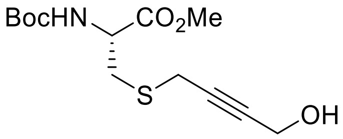

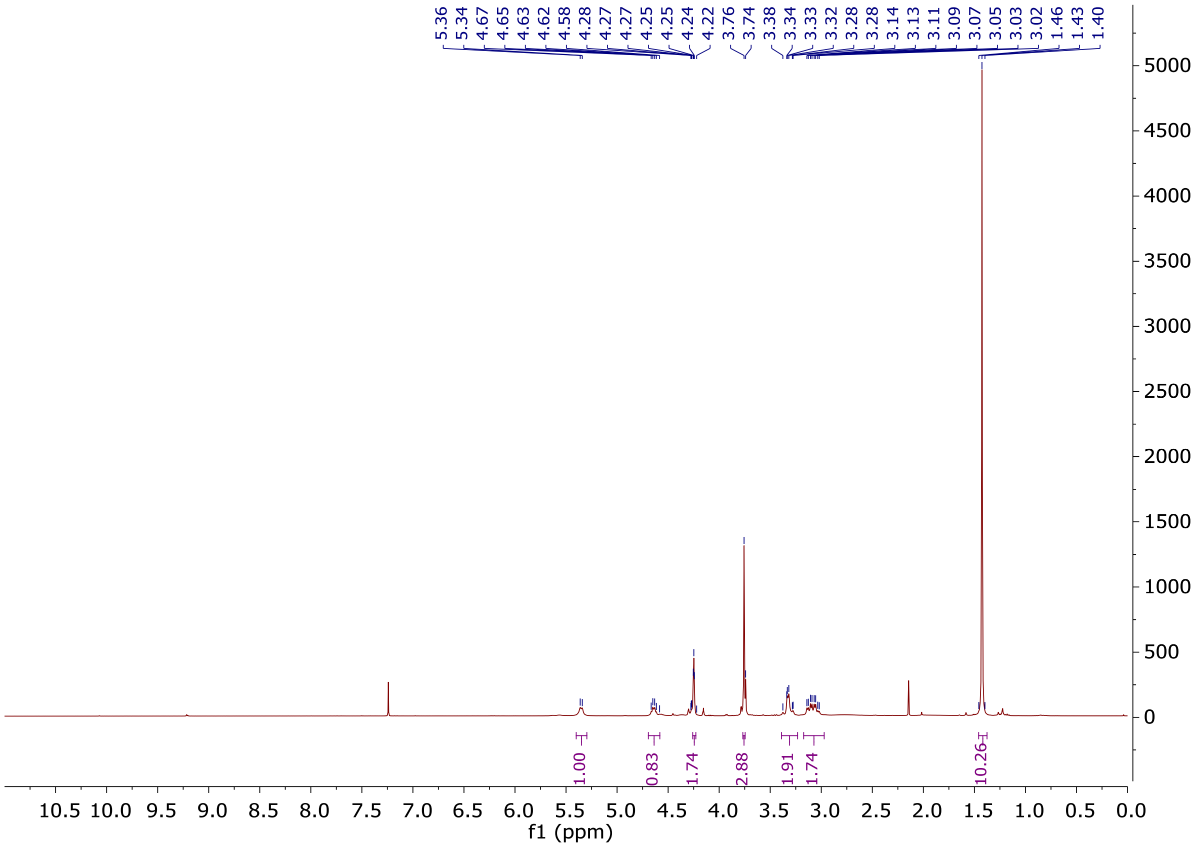

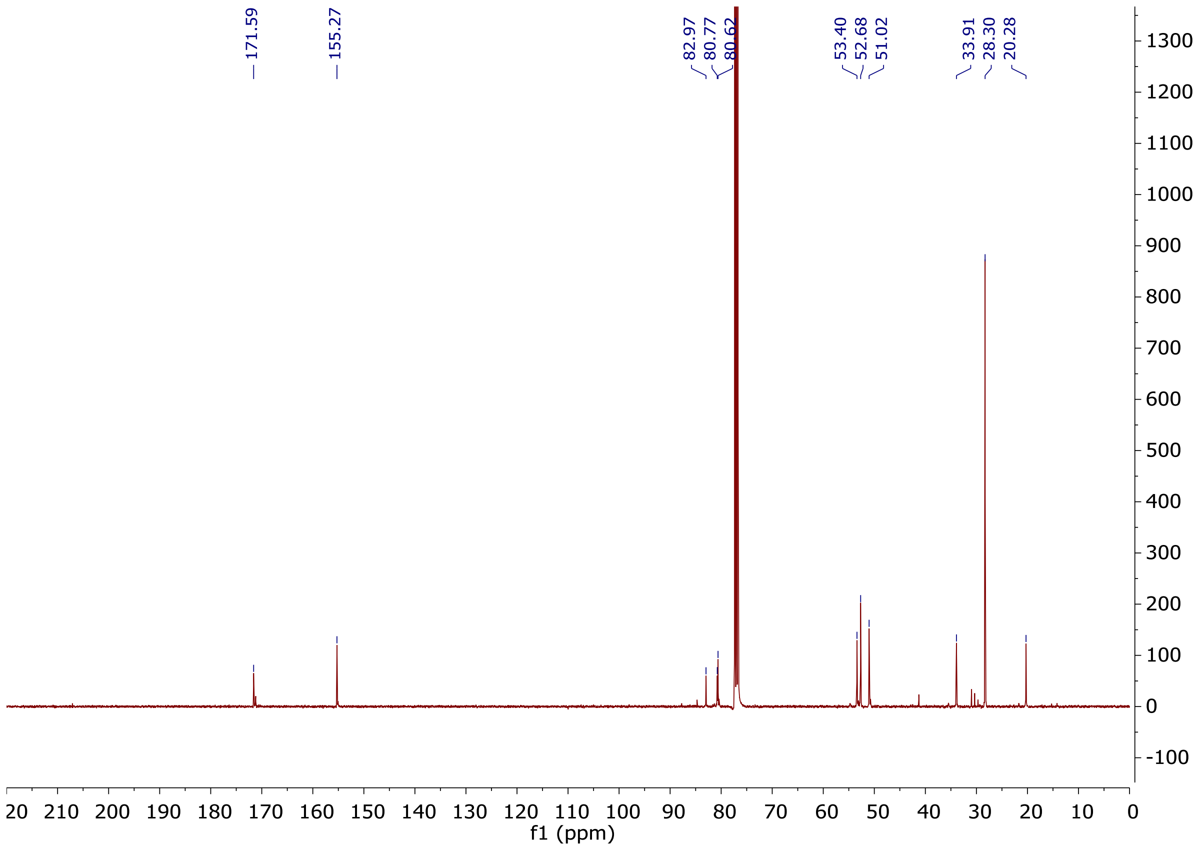
171.59
155.27
82.97
80.77
80.62
53.40
52.68
51.02
33.91
28.30
20.28
f1 (ppm)

Methyl *N*-(*tert*-butoxycarbonyl)-*S*-(4-(((4-methylcoumarin)carbamoyl)oxy)but-2-yn-1-yl)-*L*-cysteinate (18)

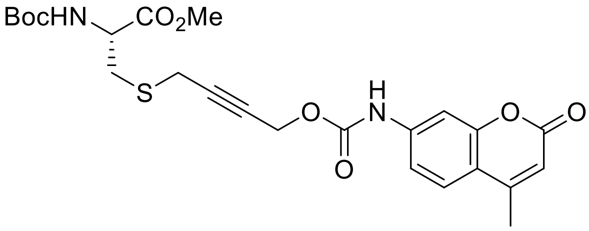

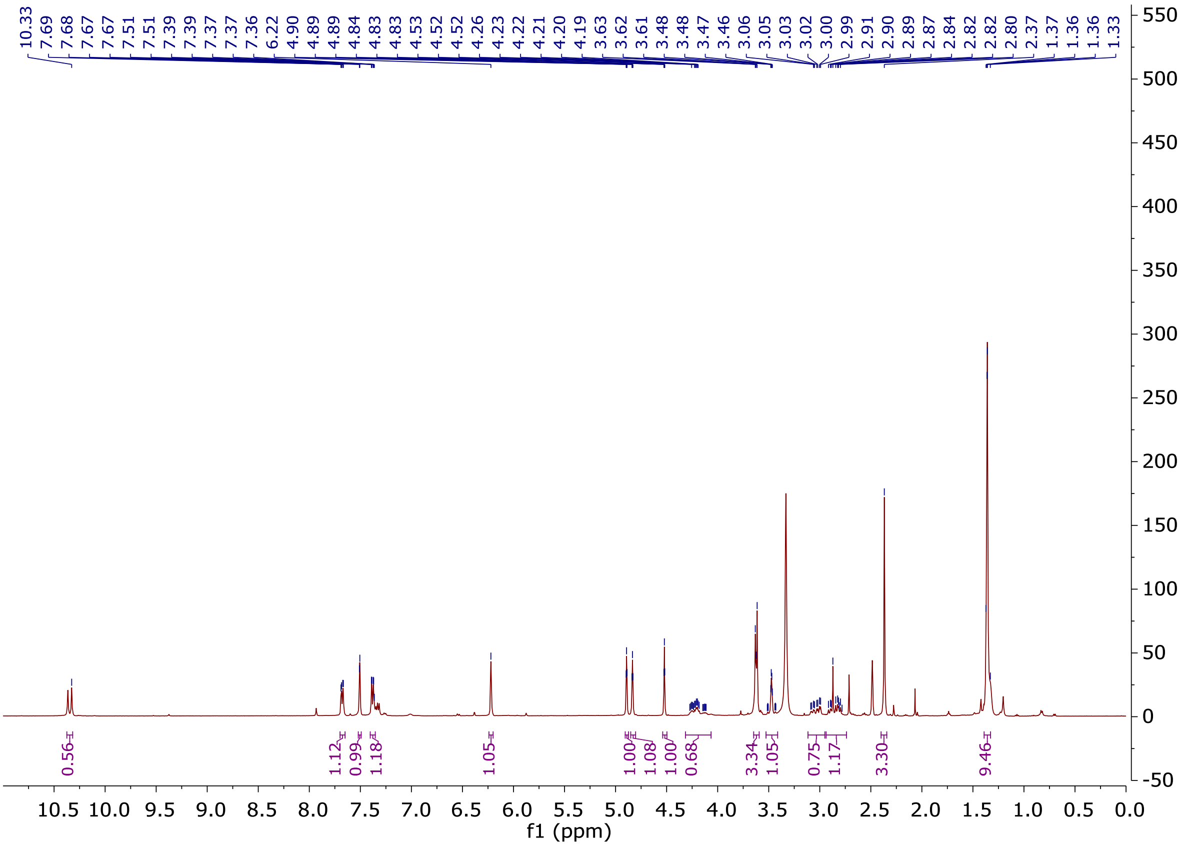

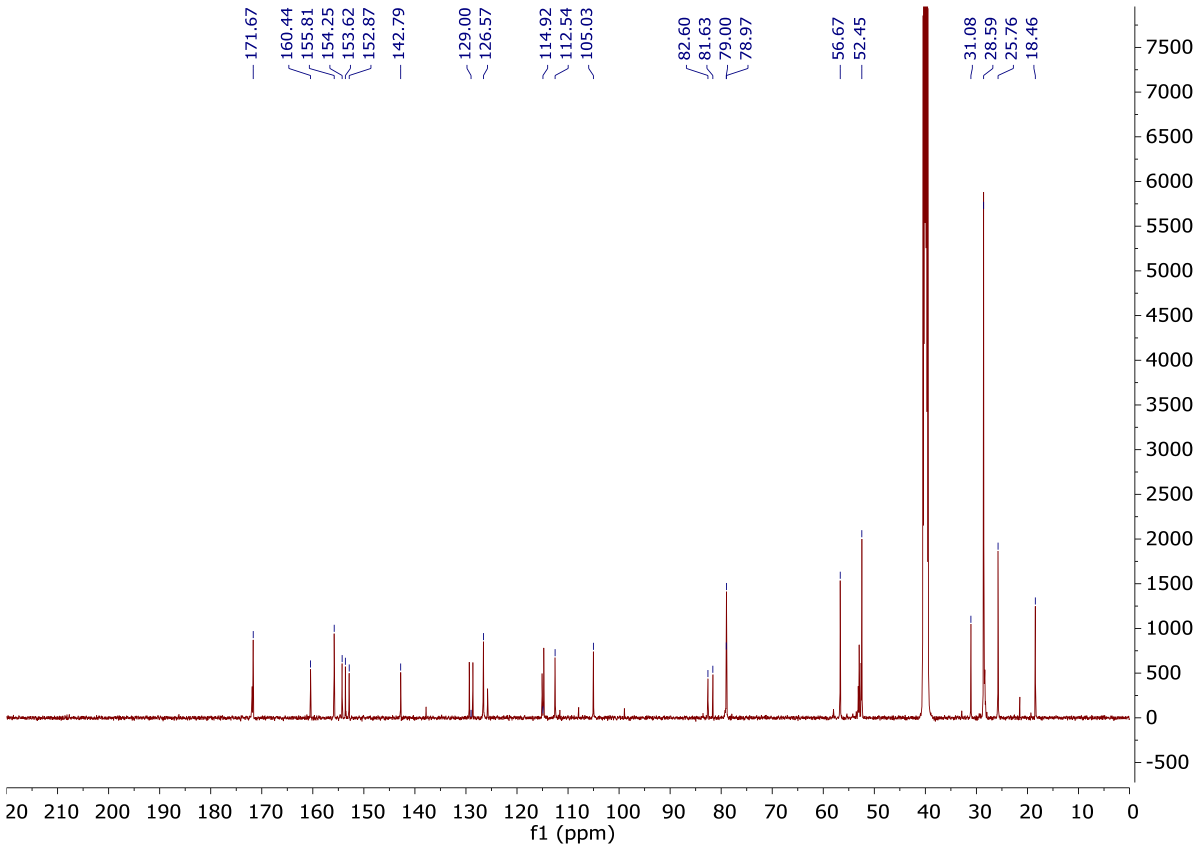
171.67
160.44
155.81
154.25
153.62
152.87
142.79
129.00
126.57
114.92
112.54
105.03
82.60
81.63
79.00
78.97
56.67
52.45
31.08
28.59
25.76
18.46
f1 (ppm)

Methyl *N*-acetyl-*S*-(4-hydroxybut-2-yn-1-yl)-*L*-cysteinate (19a)

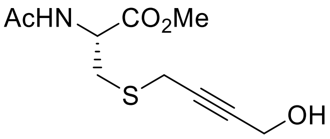

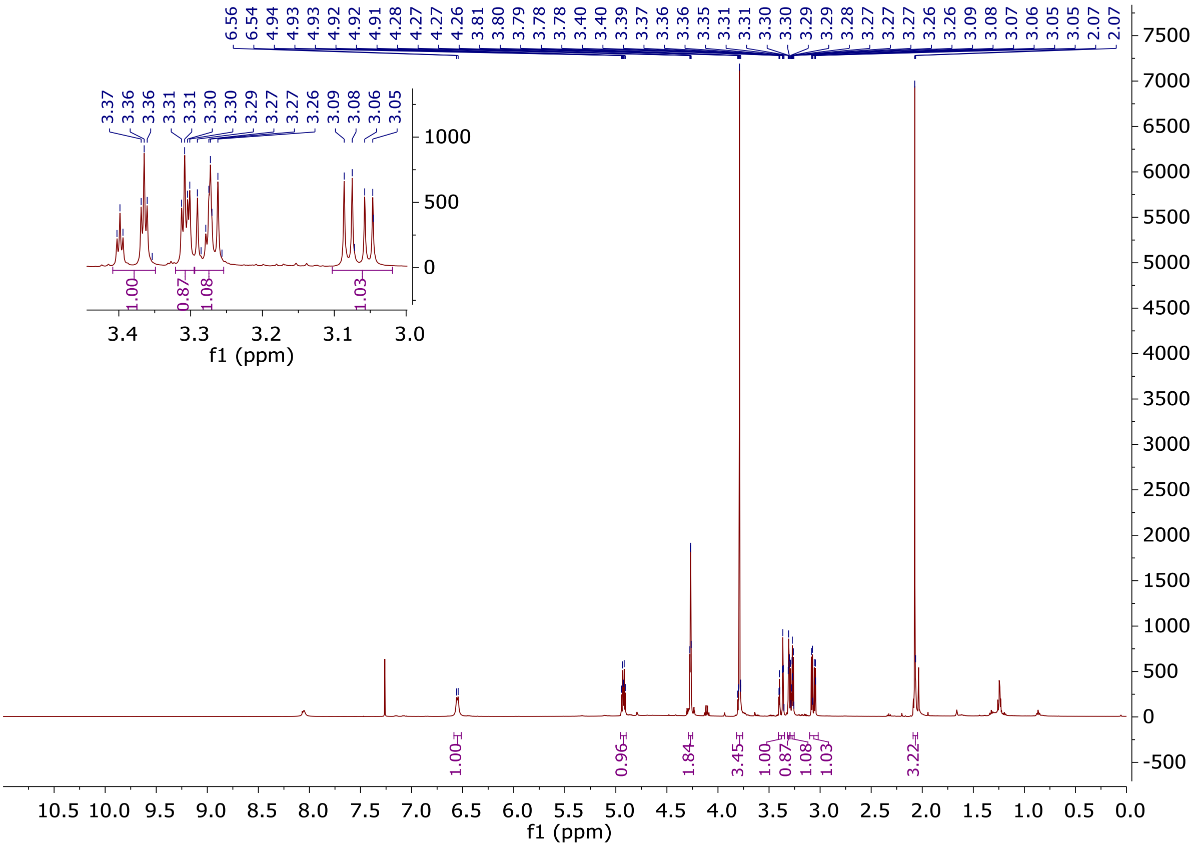

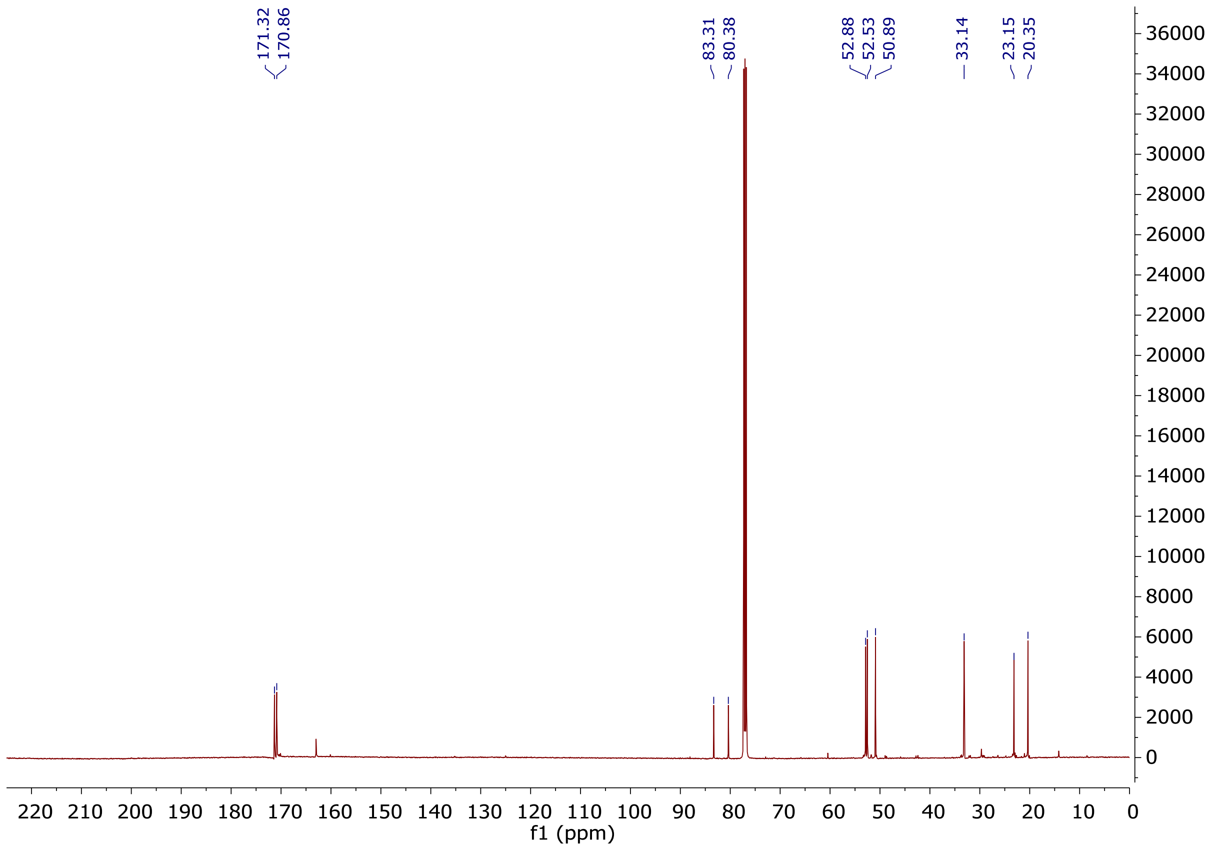
171.32
170.86
83.31
80.38
52.88
52.53
50.89
33.14
23.15
20.35
36000
34000
32000
30000
28000
26000
24000
22000
20000
18000
16000
14000
12000
10000
8000
6000
4000
2000
0
220 210 200 190 180 170 160 150 140 130 120 110 100 90 80 70 60 50 40 30 20 10 0
f1 (ppm)

Methyl *N*-acetyl-*S*-(4-(((4-methylcoumarin)carbamoyl)oxy)but-2-yn-1-yl)-*L*-cysteinate (19)

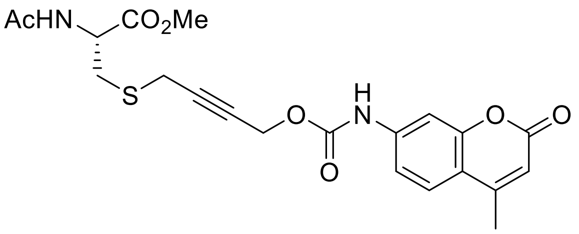

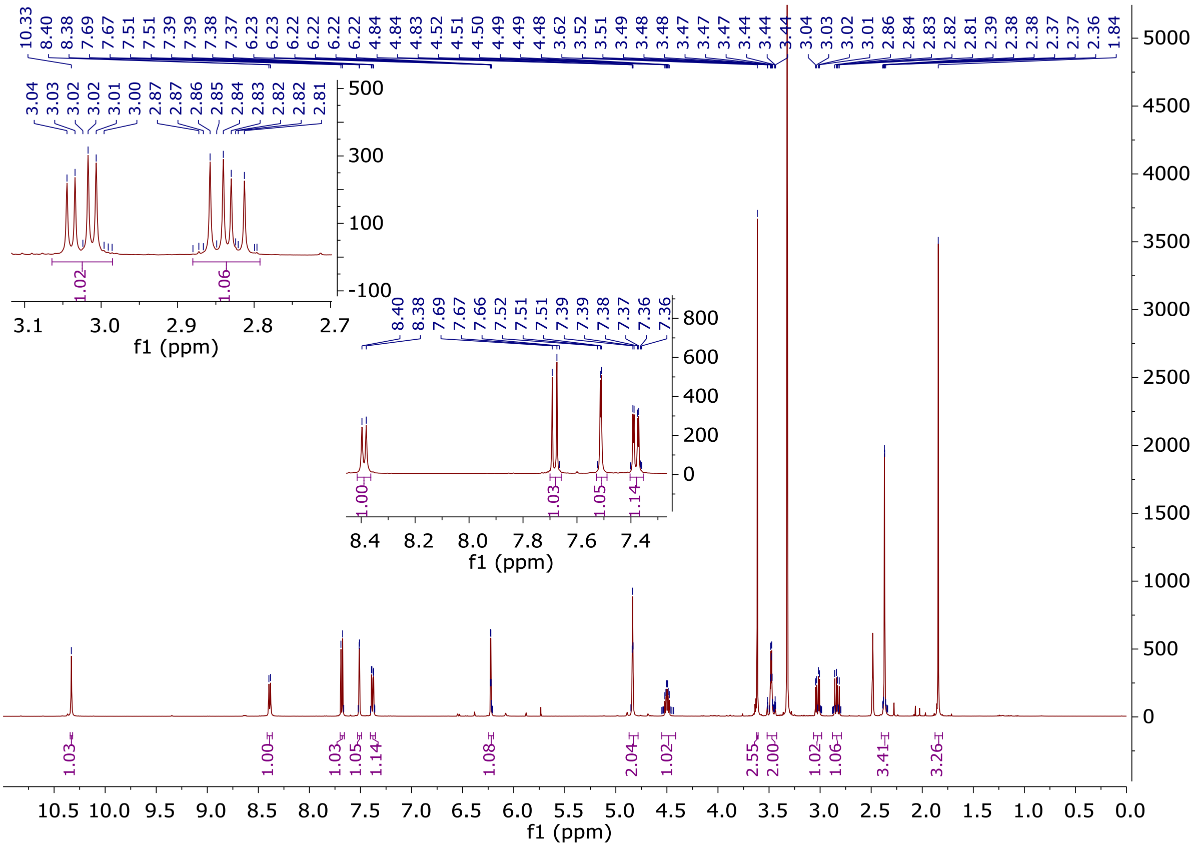

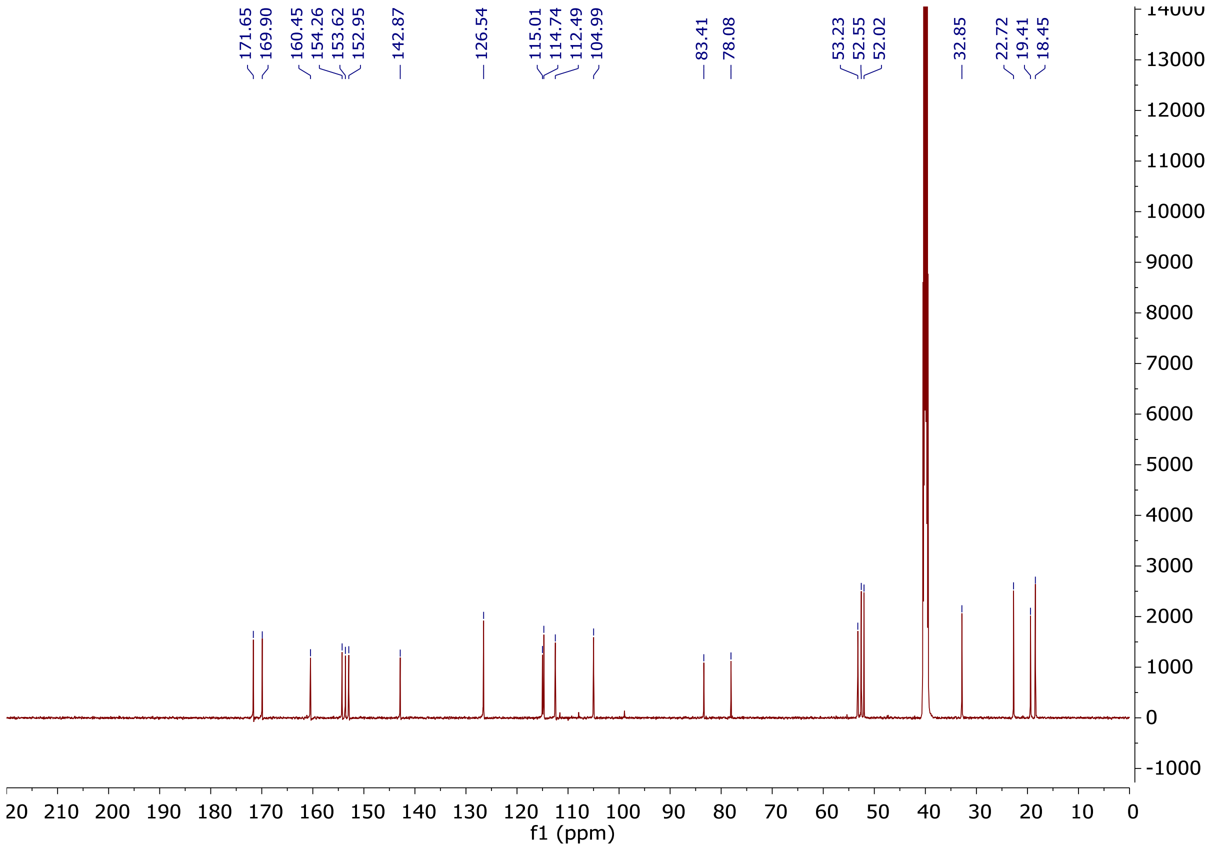
171.65
169.90
160.45
154.26
153.62
152.95
142.87
126.54
115.01
114.74
112.49
104.99
83.41
78.08
53.23
52.55
52.02
32.85
22.72
19.41
18.45
f1 (ppm)

Methyl *S*-(4-(((4-methylcoumarin)carbamoyl)oxy)but-2-yn-1-yl)-*L*-cysteinate (20)

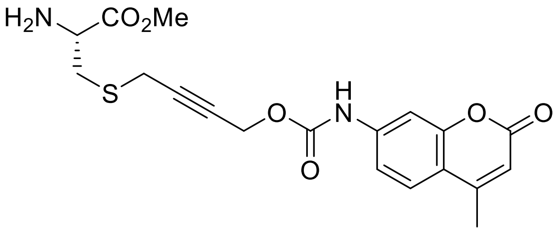

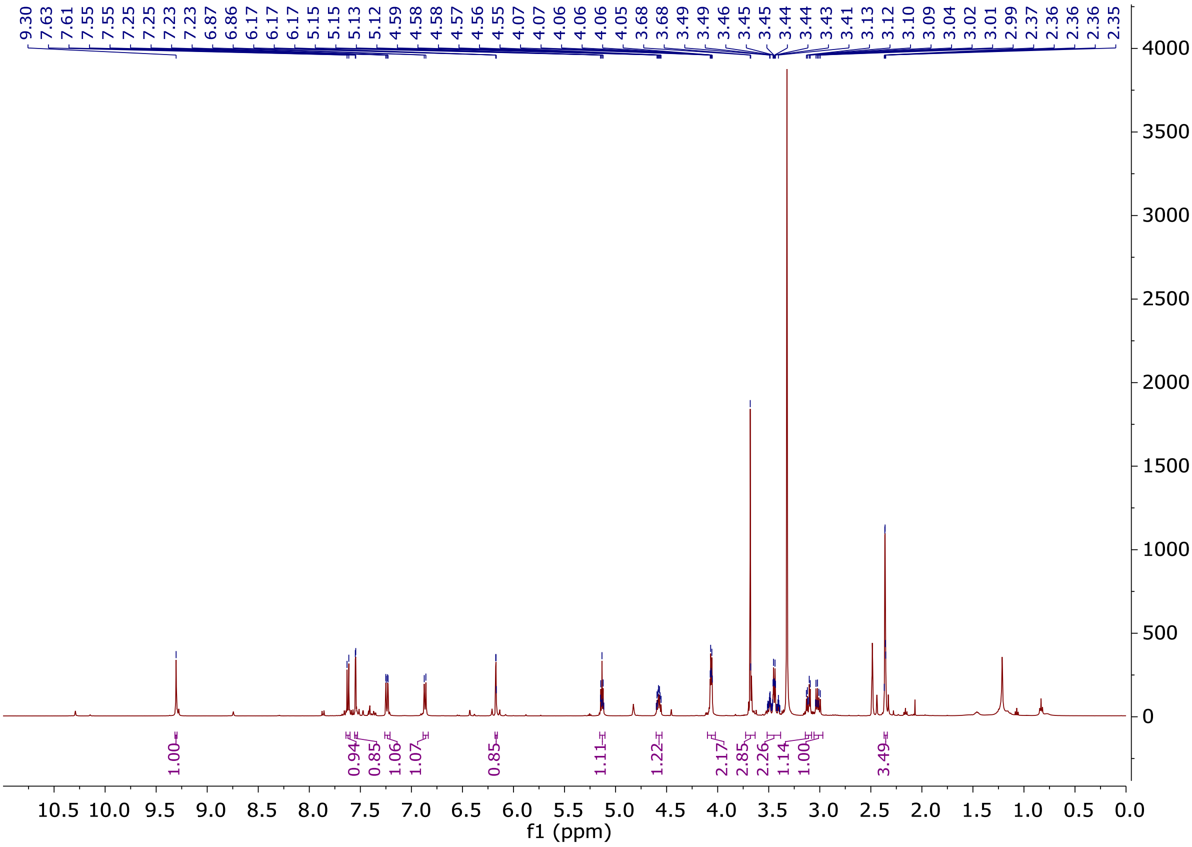

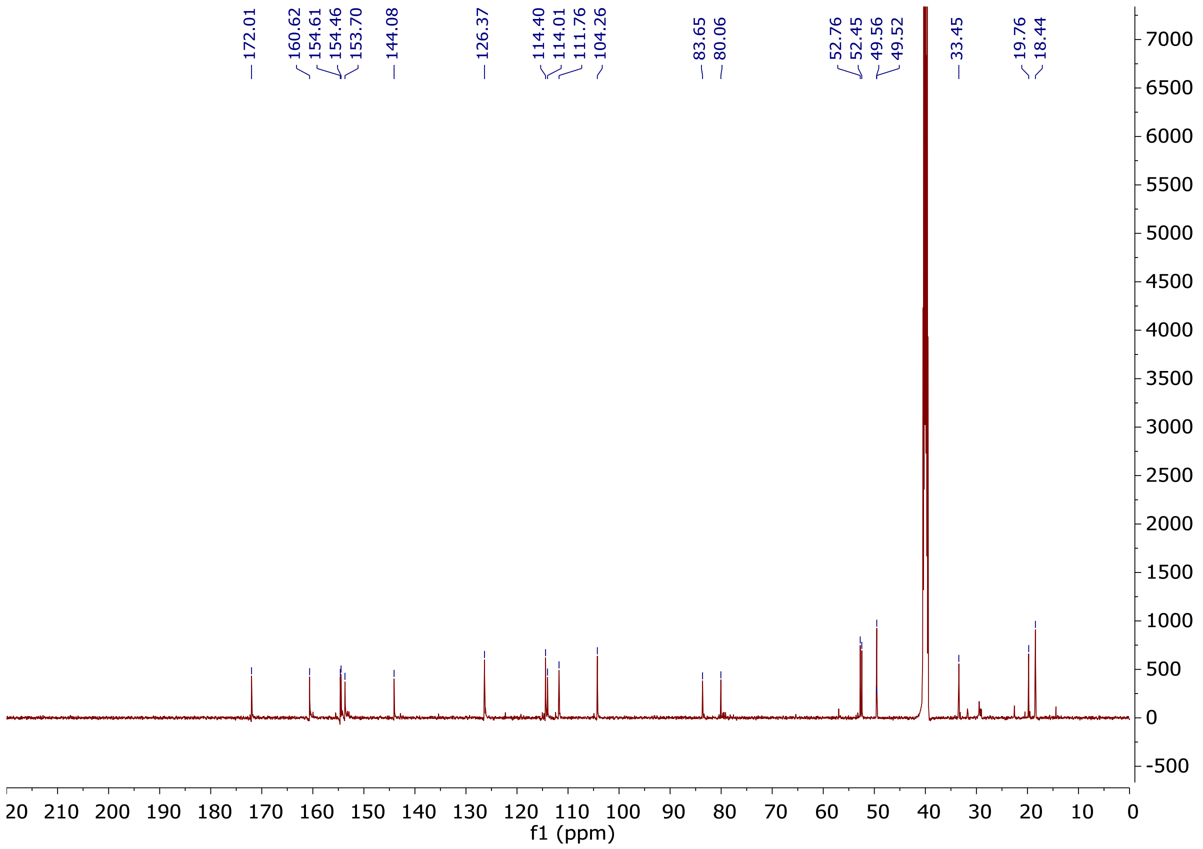
172.01
160.62
154.61
154.46
153.70
144.08
126.37
114.40
114.01
111.76
104.26
83.65
80.06
52.76
52.45
49.56
49.52
33.45
19.76
18.44
7000
6500
6000
5500
5000
4500
4000
3500
3000
2500
2000
1500
1000
500
0
-500
20 210 200 190 180 170 160 150 140 130 120 110 100 90 80 70 60 50 40 30 20 10 0
f1 (ppm)

Prop-2-yn-1-yl benzylcarbamate (22)

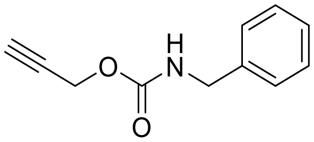

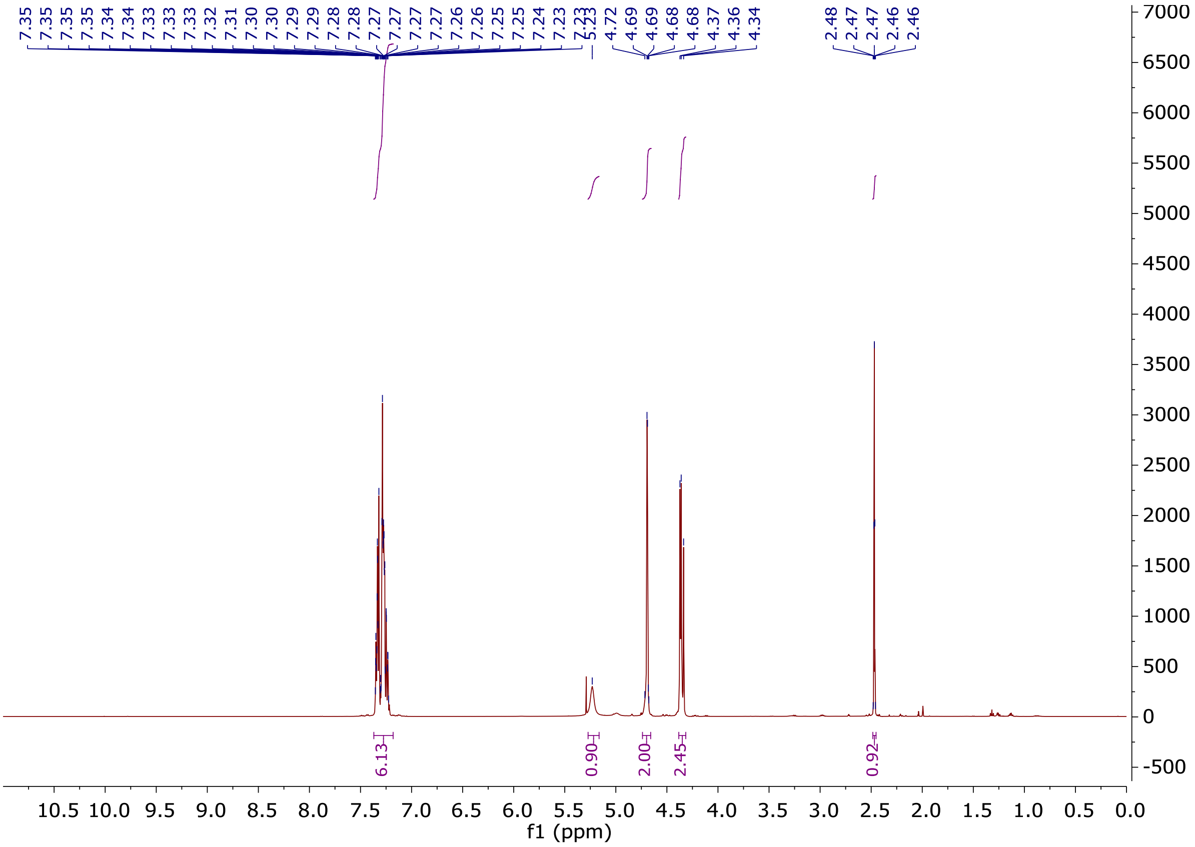

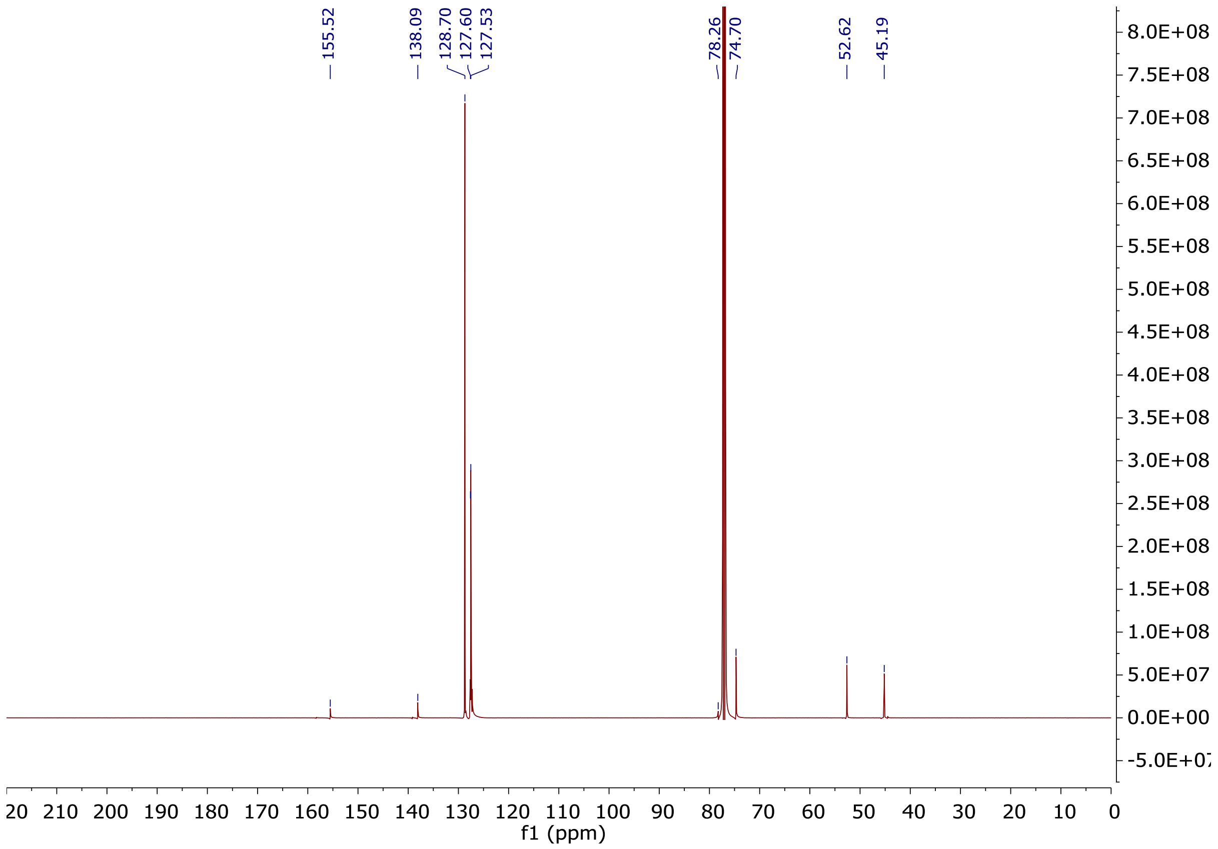
155.52
138.09
128.70
127.60
127.53
78.26
74.70
52.62
45.19
8.0E+08
7.5E+08
7.0E+08
6.5E+08
6.0E+08
5.5E+08
5.0E+08
4.5E+08
4.0E+08
3.5E+08
3.0E+08
2.5E+08
2.0E+08
1.5E+08
1.0E+08
5.0E+07
0.0E+00
20 210 200 190 180 170 160 150 140 130 120 110 100 90 80 70 60 50 40 30 20 10 0
f1 (ppm)

But-2-yn-1-yl benzylcarbamate (23)

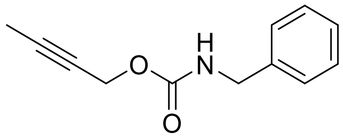

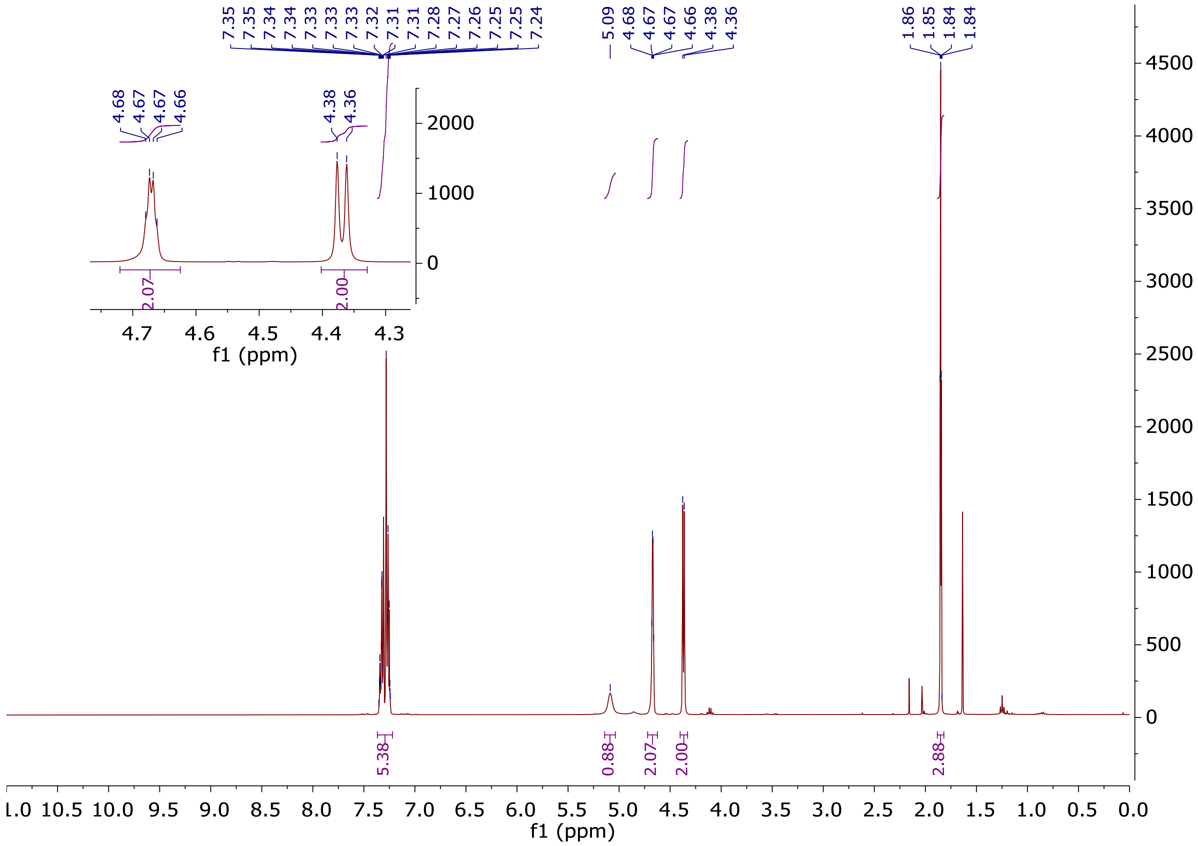

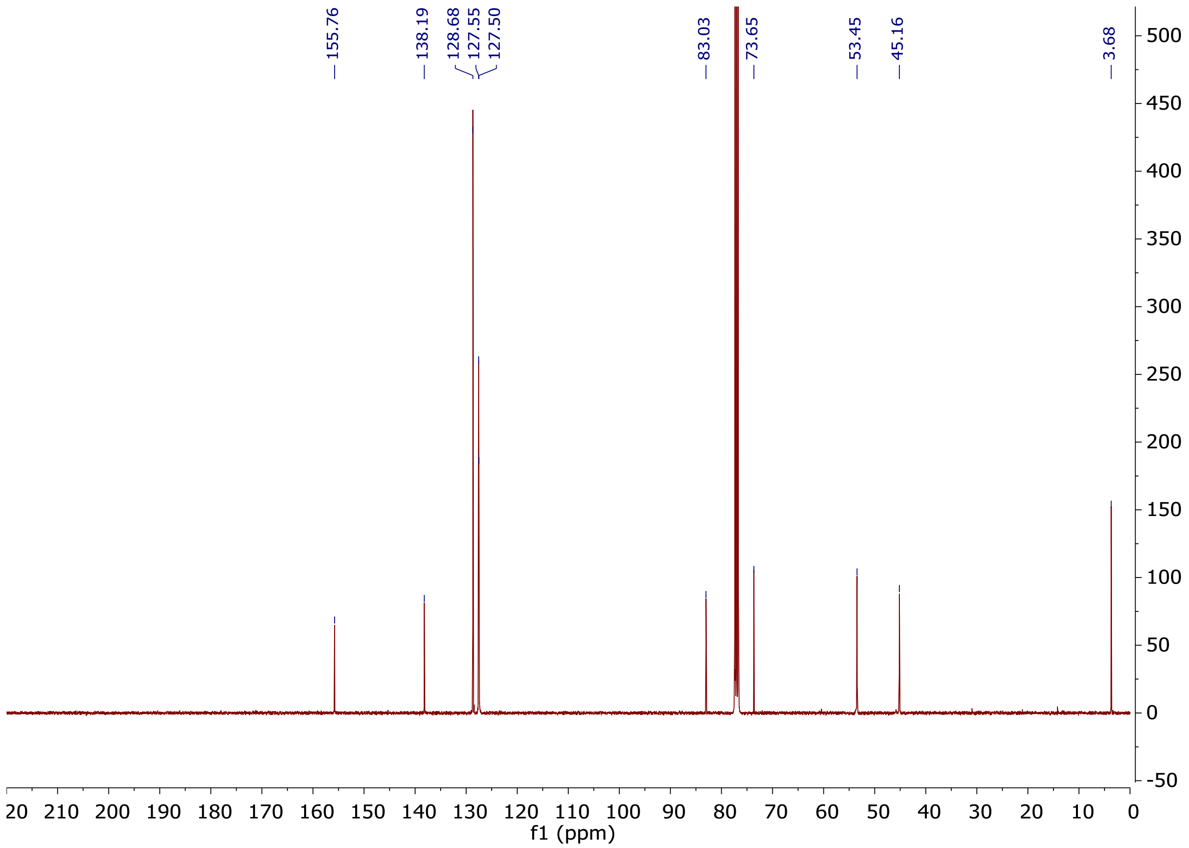
155.76
138.19
128.68
127.55
127.50
83.03
73.65
53.45
45.16
3.68
500
450
400
350
300
250
200
150
100
50
0
-50
20 210 200 190 180 170 160 150 140 130 120 110 100 90 80 70 60 50 40 30 20 10 0
f1 (ppm)

4-(tritylthio)but-2-yn-1-yl benzylcarbamate (24)

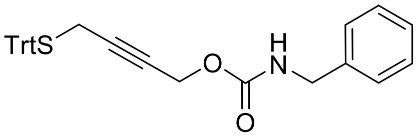

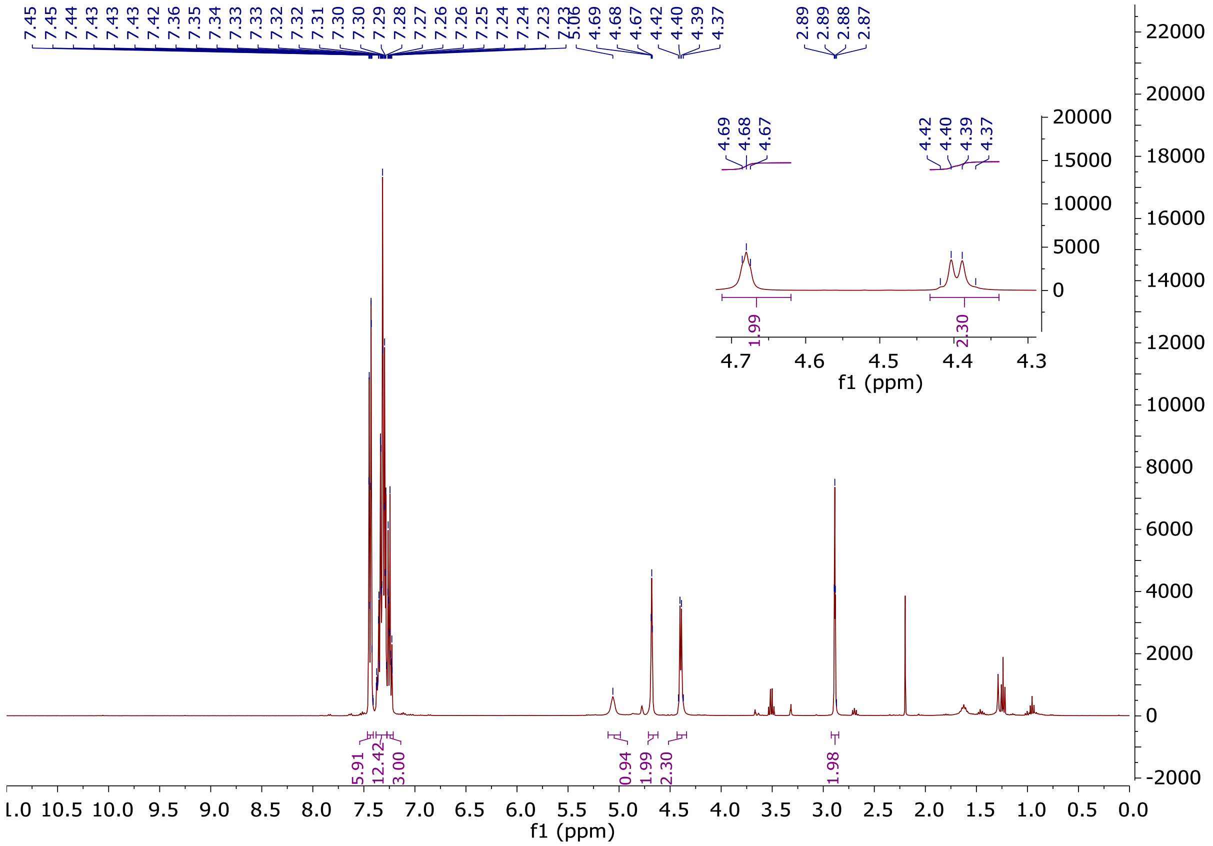

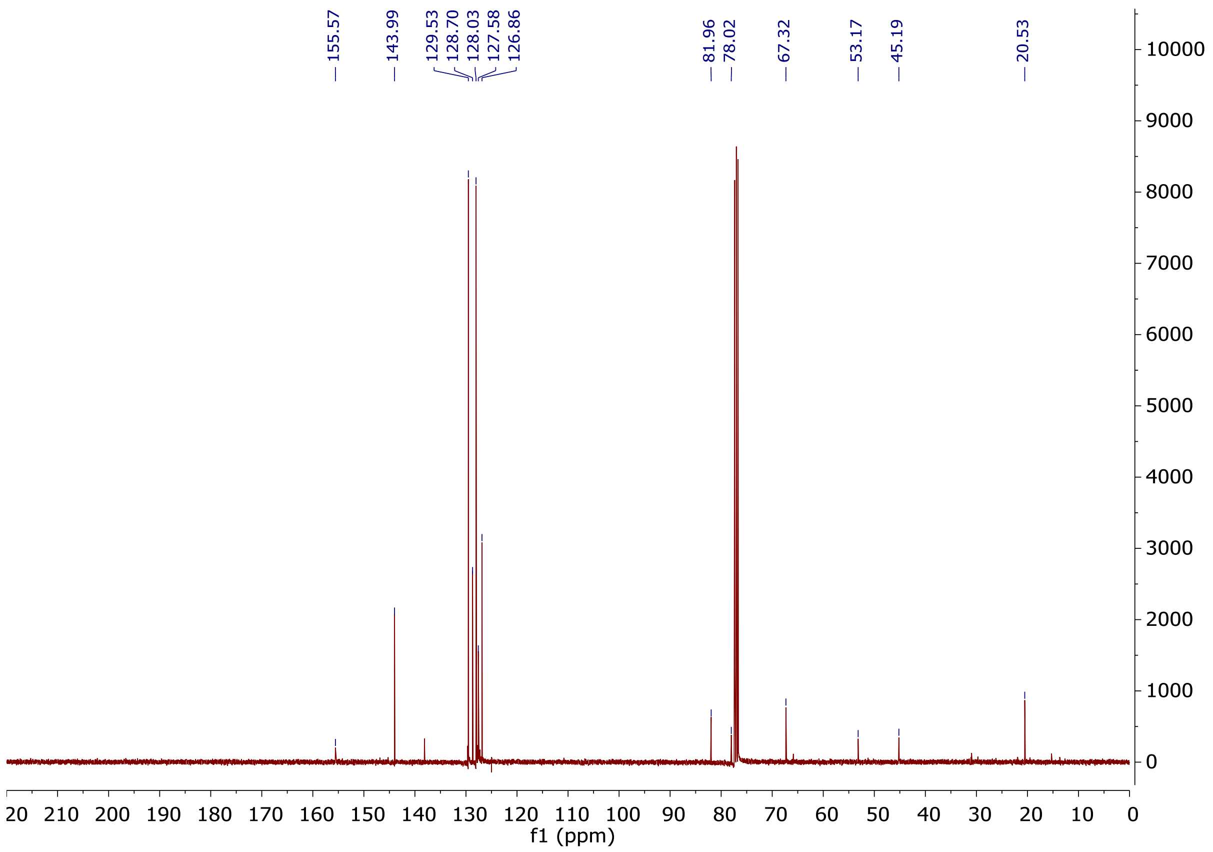
155.57
143.99
129.53
128.70
128.03
127.58
126.86
81.96
78.02
67.32
53.17
45.19
20.53
10000
9000
8000
7000
6000
5000
4000
3000
2000
1000
0
20 210 200 190 180 170 160 150 140 130 120 110 100 90 80 70 60 50 40 30 20 10 0
f1 (ppm)

7. References

(1) J. Wang, B. Cheng, J. Li, Z. Zhang, W. Hong, X. Chen and P. R. Chen, *Angew. Chem. Int. Ed.*, 2015, **54**, 5364.

(2) T. Calogeropoulou, P. Angelou, A. Detsi, I. Fragiadaki and E. Scoulica, *J. Med. Chem.*, 2008, **51**, 897.

(3) V. Druais, M. J. Hall, C. Corsi, S. V. Wendeborn, C. Meyer and J. Cossy, *Org. Lett.*, 2009, **11**, 935.

(4) A. -I. Hernández, J. Balzarini, A. Karlsson, M. -J. Camarasa and M. -J. Pérez-Pérez, *J. Med. Chem.*, 2002, **45**, 4254.

(5) A. Padwa, M. Meske and Z. Ni, *Tetrahedron*, 1995, **51**, 89.

(6) M. F. Rettig, R. M. Wing and G. R. Wiger *J. Am. Chem. Soc.*, 1981, **103**, 2980.

(7) D. Drew, J. R. Doyle and A. G. Shaver, *Inorg. Synth.*, 1990, **28**, 346.

(8) M. von Delius, F. Hauke and A. Hirsch, *Eur. J. Org. Chem.*, 2008, **24**, 4109.

(9) U. Streit, F. Birbaum, A. Quattropani and C. G. Bochet, *J. Org. Chem.*, 2013, **78**, 6890.

(10) C. H. Kaschula, R. Hunter, N. Stellenboom, M. R. Caira, S. Winks, T. Ogunleye, P. Richards, J. Cotton, K. Zilbeyaz, Y. Wang, V. Siyo, E. Ngarande and M. I. Parker, *Eur. J. Med. Chem.*, 2012, **50**, 236.

(11) S. B. Jones, B. Simmons and D. W. C. MacMillan, *J. Am. Chem. Soc.*, 2009, **131**, 13606.

(12) R. Madan, R. C. Anand and I. K. Varma, *J. Therm. Anal. Calorim.*, 2000, **59**, 531.

(13) H. Li, G. A. Grasa and T. J. Colacot, *Org. Lett.*, 2010, **12**, 3332.

(14) T. J. Colacot, G. A. Grasa and H. Li, Preparation of a metal complex. WO2010128316 A1, 2010.
